# Structure and Function of Canine SP-C Mimic Proteins in Synthetic Surfactant Lipid Dispersions

**DOI:** 10.3390/biomedicines12010163

**Published:** 2024-01-12

**Authors:** Frans J. Walther, Alan J. Waring

**Affiliations:** 1 Lundquist Institute for Biomedical Innovation at Harbor-UCLA Medical Center, Torrance, CA 90502, USA; 2 Department of Pediatrics, David Geffen School of Medicine, University of California Los Angeles, Los Angeles, CA 90095, USA; 3 Department of Medicine, David Geffen School of Medicine, University of California Los Angeles, Los Angeles, CA 90095, USA

**Keywords:** surfactant protein B (SP-B), surfactant protein C (SP-C), surfactant protein peptide mimics, synthetic lung surfactant, AI modeling, molecular dynamics (MD), circular dichroism (CD), Fourier Transform Infrared (FTIR) spectroscopy, electron spin resonance (ESR), captive bubble surfactometry (CBS)

## Abstract

Lung surfactant is a mixture of lipids and proteins and is essential for air breathing in mammals. The hydrophobic surfactant proteins B and C (SP-B and SP-C) assist in reducing surface tension in the lung alveoli by organizing the surfactant lipids. SP-B deficiency is life-threatening, and a lack of SP-C can lead to progressive interstitial lung disease. B-YL (41 amino acids) is a highly surface-active, sulfur-free peptide mimic of SP-B (79 amino acids) in which the four cysteine residues are replaced by tyrosine. Mammalian SP-C (35 amino acids) contains two cysteine-linked palmitoyl groups at positions 5 and 6 in the N-terminal region that override the *β*-sheet propensities of the native sequence. Canine SP-C (34 amino acids) is exceptional because it has only one palmitoylated cysteine residue at position 4 and a phenylalanine at position 5. We developed canine SP-C constructs in which the palmitoylated cysteine residue at position 4 is replaced by phenylalanine (SP-Cff) or serine (SP-Csf) and a glutamic acid-lysine ion-lock was placed at sequence positions 20–24 of the hydrophobic helical domain to enhance its alpha helical propensity. AI modeling, molecular dynamics, circular dichroism spectroscopy, Fourier Transform InfraRed spectroscopy, and electron spin resonance studies showed that the secondary structure of canine SP-Cff ion-lock peptide was like that of native SP-C, suggesting that substitution of phenylalanine for cysteine has no apparent effect on the secondary structure of the peptide. Captive bubble surfactometry demonstrated higher surface activity for canine SP-Cff ion-lock peptide in combination with B-YL in surfactant lipids than with canine SP-Csf ion-lock peptide. These studies demonstrate the potential of canine SP-Cff ion-lock peptide to enhance the functionality of the SP-B peptide mimic B-YL in synthetic surfactant lipids.

## 1. Introduction

Lung surfactant is a mixture of lipids and proteins synthesized by alveolar type 2 cells and secreted into the lung alveoli where it reduces surface tension at the air–liquid interface [1,2]. Mammalian lung surfactant contains about 80% phospholipids, 10% neutral lipids, and 10% protein. Major components are the hydrophobic surfactant proteins B and C (SP-B and SP-C) and the phospholipids dipalmitoyl phosphatidylcholine (DPPC) and phosphatidylglycerol (PG). SP-B and SP-C enhance the adsorption and spreading of the phospholipids at the air–liquid interface, thereby promoting the surface tension-lowering properties of surfactant [3].

Respiratory failure (respiratory distress syndrome, RDS) is one of the major health problems in premature infants born at less than 32 weeks of gestation and is caused by surfactant deficiency due to lung immaturity [4]. Surfactant deficiency leads to alveolar collapse and atelectasis that can be reversed with exogenous surfactant and respiratory support. In the early 1990s, the application of animal-derived lung surfactant drastically reduced mortality and morbidity in premature infants [5,6], but limited supplies and high costs have led to the current search for an advanced synthetic lung surfactant formulation consisting of a mixture of synthetic surfactant phospholipids similar to native surfactant and highly surface-active peptide mimics of the hydrophobic surfactant proteins B and C (SP-B and SP-C). Our group has developed several highly functional SP-B peptide mimics, such as Super Mini-B [7] and B-YL [8,9], and we are interested in the addition of a stable SP-C peptide mimic [10].

SP-B (79 amino acids) belongs to the saposin protein superfamily and plays a pivotal role in lung surfactant as SP-B deficiency is life-threatening [11]. B-YL (41 amino acids) is a highly surface-active, sulfur-free peptide mimic of SP-B based on the N- and C-domains of SP-B, covalently joined with a turn, and folding as an α-helix hairpin mimicking the properties of these domains in SP-B [8]. Like SP-B, B-YL is involved in organizing the lipid bilayer and occupies a peripheral bilayer position.

**Figure 1:**
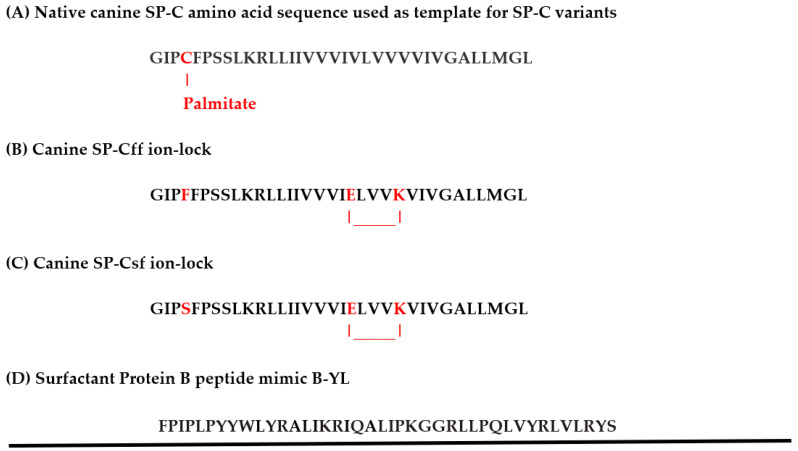
Amino acid sequences for canine SP-C protein, SP-Cff ion-lock designer protein, and B-YL SP-B peptide mimic. (**A**) Native canine SP-C amino acid sequence with cysteine thioester-linked palmitic acid highlighted in red [17]. (**B**) Canine SP-Cff ion-lock amino acid sequence with phenylalanine substituted for Cys-Palmitate in red at residue 4 and ion-lock pair E20/K24 in red highlight at residues 20–24 in the hydrophobic polyvaline amino acid sequence. (**C**) Canine SP-Csf ion-lock amino acid sequence with serine substituted for Cys-Palmitate in red at residue 4 and ion-lock pair E20/K24 in red highlight at residues 20–24 in the hydrophobic polyvaline amino acid sequence. (**D**) Amino acid sequence of the SP-B peptide mimic B-YL.

SP-C (35 amino acids) is a highly hydrophobic surfactant protein with a valine-rich alpha helix that spans the lipid bilayer (PDB Accession Code: 1SPF) [12,13]. It has a monomer molecular weight of 4.2 kDa, and includes cysteine-linked palmitoyl groups at positions 5 and 6 in mammals, except for canines. SP-C plays a secondary role in surfactant function, but a lack of SP-C may lead to progressive interstitial lung disease in children [14]. In vitro studies suggest that SP-C stabilizes bilayer ensembles that remain attached to the monolayer at the air–liquid interface and may function as a surfactant lipid reservoir that is important to stabilize the monolayer [15,16]. Canine SP-C (34 amino acids) varies from other mammalian SP-Cs due to the presence of only one cysteine-linked palmitoyl group in the N-terminal segment at position 4 and phenylalanine at position 5 [17,18] (Figure 1). To test the structure and function of a sulfur-free SP-C peptide mimic, we developed canine SP-C constructs in which the palmitoylated cysteine residue at position 4 is replaced by phenylalanine (SP-Cff) or serine (SP-Csf) in combination with a glutamic acid-lysine ion-lock at sequence positions 20–24 of the hydrophobic helical domain to enhance its alpha helical propensity [10].

The synthetic surfactant lipid composition used in these studies was selected to mimic that observed in native lung surfactant regarding the molecular species. These include approximately 50% dipalmitoyl phosphatidylcholine (DPPC), while the remaining lipids have at least one unsaturated acyl chain with polar head groups, such as zwitterionic phosphatidylcholine and anionic phosphatidylglycerol [19,20,21,22,23].

## 2. Materials and Methods

### 2.1. Materials

Peptide synthesis reagents were purchased from AnaSpec (Fremont, CA, USA), high performance liquid chromatography (HPLC) solvents from Fisher Scientific (Pittsburgh, PA, USA), and all other chemicals from MilliporeSigma (St. Louis, MO, USA). Dipalmitoyl phosphatidylcholine (DPPC), 1-palmitoyl-2-oleoyl-sn-glycero-3-phosphocholine (POPC), and palmitoyl-oleoyl-phosphatidylglycerol (POPG) were purchased from Avanti Polar Lipids (Alabaster, AL, USA). The clinical porcine surfactant Poractant Alfa (Curosurf^®^) was obtained from Chiesi Farmaceutici, Parma, Italy.

### 2.2. Canine SP-Cff and SP-Csf Ion-Lock and B-YL Peptide Synthesis

B-YL and canine SP-Cff and SP-Csf ion-lock peptides (Figure 1) were synthesized using standard Fmoc procedures with HMPB NovaPEG resins (Millipore-Sigma, St. Louis, MO, USA) using a Liberty Microwave Peptide (CEM Corp, Matthews, NC, USA) synthesizer. SP-Cff and SP-Csf ion-lock peptides were synthesized via triple coupling at each residue to optimize produce yield. The SP-B mimic peptide B-YL was synthesized and purified as detailed previously [9]. SP-C peptides were purified using reverse phase high-performance liquid chromatography like other SP-C ion-lock peptides [10]. TFA (Trifluroacetate) counter-ions that pair with peptide cationic residues and interfere with protein spectra Amide I conformational bands were eliminated by dissolving the sample in HCl and freeze drying [24]. The molecular masses of all peptides were confirmed via Maldi TOF mass spectrometry.

### 2.3. Surfactant Preparations

The surfactant peptides SP-Cff ion-lock, SP-Csf ion-lock, and B-YL alone or in combinations were formulated with synthetic surfactant lipids (35 mg/mL) that consisted of 50% DPPC, 30% POPC, and 20% POPG (5:3:2) by weight. Synthetic surfactant lipids were dissolved in chloroform and the desired concentration of synthetic surfactant peptides was dissolved in trifluoroethanol (TFE) and co-solvated with the lipids to optimize the peptide structure. The lipid–peptide mixture was dried under nitrogen gas to remove the chloroform-TFE solvents, followed by freeze drying of the resulting lipid–peptide film to remove residual solvent. The freeze-dried lipid–peptide film was then dispersed with phosphate buffered saline at 60 °C by rotating the dispersion at 200 rpm for one hour. This protocol resulted in a multilamellar lipid–peptide dispersion that was used for in vitro and in vivo testing after storage for at least 12 h at 5 °C. The clinical porcine surfactant Curosurf that contains both native SP-B and SP-C was used as a positive control.

### 2.4. Structure Prediction—Alpha Fold (AI Modeling)

Initial structures for canine SP-C peptides were determined using artificial intelligence-based predictive modeling that uses a neural network-based protein structure prediction with machine learning by incorporating multi-amino acid sequence alignments into the design of the deep learning prediction algorithm [25]. The three-dimensional (3D) structural models for canine SP-C and related SP-Cff and SP-Csf ion-lock variants were determined using AlphaFold artificial intelligence (AI) software. The AlphaFold program was run through the Chimera X (version 1.6.1) molecular modeling environment at https://www.cgl.ucsf.edu/chimera/docs/relnotes.html (accessed on 26 June 2023).

These initial structural models for the peptides were then further refined via molecular dynamics in relevant surfactant lipid multilayer environments to better characterize possible structure–activity correlations of peptide-mediated bilayer–bilayer interactions. A preliminary model of the peptide was oriented in the surfactant lipid synthetic bilayer construct by using OPM data base-PPM web server utility [26]. This initial peptide orientation was then ported to the Charmm Membrane Builder (http://www.charmm-gui.org/?doc=input/membrane.bilayer, accessed on 26 June 2023) and incorporated into a surfactant lipid bilayer consisting of a total of 292 lipids followed by adding potassium and chloride ions to achieve an electrically neutral system in a rectangular simulation box [27]. The simulation system was hydrated with TIP3 water and downloaded from the Charmm GUI internet site by selecting the Gromacs molecular simulation option prior to molecular dynamics refinement of the peptide–lipid ensemble.

Molecular Dynamics simulation runs were made using the Charmm 36 m all atom force field (Version 2021.5) available at (http://www.gromacs.org, accessed on 26 June 2023). Typical protocol for this simulation includes system minimization followed by a six-step equilibration process at 311° K. Parameters used included both NVT (constant number, volume, temperature) and NPT (constant number, pressure, temperature), thereby permitting bilayer lipids to optimize their orientation around the inserted peptide. Detailed equilibration protocols for other system molecular interactions such as Berendsen strategies for pressure coupling and post equilibration dynamics production are available from the Charmm-GUI website (http://www.charmm-gui.org, accessed on 26 June 2023). Simulations were analyzed using Gromacs analysis software (http://www.gromacs.org, accessed on 26 June 2023).

Molecular graphics were rendered using Pymol Version 2.2.3. Coordinates for the SP-C variants are available in Supplementary Files S1–S3.

### 2.5. Circular Dichroic Characterization of SP-Cff ion-Lock Peptide in Membrane Mimic Micelles

To gain insights into the unique secondary structure of the canine SP-Cff ion-lock peptide, the overall secondary structure of the peptide was compared to the membrane mimic suspension sodium dodecyl sulfate (SDS) anionic detergent micelles that minimize the effects of light scattering in this type of dispersion. CD spectra of the canine SP-Cff ion-lock peptide in the SDS–buffer structure-promoting environment was measured with a Chirascan V100 spectropolarimeter (Applied Photophysics, Surrey, UK). This instrument was routinely calibrated for wavelength and optical rotation using 10-camphorsulphonic acid [28]. The sample solutions of the anionic detergent SDS were scanned using 0.01 cm pathlength cells at a rate of 50 nm per minute, a sample interval of 1 nm, and a temperature of 37 °C. Peptide concentration was 100 µM in a sample solution of SDS:PBS buffer (20 mM detergent:10 mM buffer, pH 7.5) scanning at a wavelength range of 190 to 260 nm. Sample spectra were baseline corrected by subtracting spectra of peptide-free solution from that of the peptide containing solution and expressed as the Mean Residue Ellipticity [θ]_MRE_ as shown in Equation (1):

[θ]_MRE_ = ([θ] × 100)/(l × c × N) (1)

The symbol θ is the measured ellipticity in millidegrees, l is the pathlength in cm, N is the number of residues in the peptide, and c is the concentration of the peptide in mM. Analysis of the CD spectra to determine the approximate contributions of the secondary conformations in the membrane mimic SDS micellar environment was done with SELCON 3 [28], using the option 7 basis set for membrane proteins implemented from the DichroWeb suite of programs [29,30,31] (http://dichroweb.cryst.bbk.ac.uk (accessed on 12 December 2023).

### 2.6. Secondary Structure Analysis and Orientation of Canine SP-C Ion-Lock Peptide Mimics in Synthetic Surfactant Lipids with FTIR Spectroscopy

Unlike CD spectroscopy of MLV (Multi-Lamellar Vesicles) dispersions that are subject to excessive light-scattering artifacts, we used FTIR spectroscopy coupled with ATR (Attenuated Total Refection) surface sampling for the determination of the secondary conformation and orientation of peptides and proteins in membrane-like lipid multilayer systems [32]. Infrared spectra were measured at 37 °C using a JASCO FTIR 4600LE spectrometer (Easton, MD, USA) with a deuterium triglyceride sulfate (DTGS) detector, and an ATR-pro sample apparatus with a monolithic diamond attenuated total reflectance sample system. Spectra were averaged over 64 scans at a gain of 4 and a resolution of 4 cm^−1^ [33]. The FTIR measurements of SP-C peptides in synthetic surfactant lipids were accomplished using co-solvation of peptide–lipid samples (mole ratio of lipid to peptide 10:1) in HFIP with the surfactant lipid molecular species of DPPC:POPC:POPG (weight ratio, 5:3:2) spread onto the ATR crystal surface. The sample solvent was then evaporated using dry nitrogen gas that resulted in the production of a dry multilayer lipid peptide film. This dry film was then hydrated to at least 35% with deuterium vapor flowed in nitrogen gas for 1 h prior to acquiring spectra. The spectra for peptide per se in lipid films were then obtained by subtracting the lipid spectrum with D_2_O from that of peptide in lipid with D_2_O hydration. Infrared spectra were subsequently recorded for the lipid–peptide films at 37 °C. The contributions of the conformations of the peptide amide I to the absorption envelope between 1700 and 1600 cm^−1^ was determined using Fourier self-deconvolution curve-filling software (GRAMS/AI8, version 8.0, Themo Electron Corporation, Waltham, MA, USA). FTIR frequency limits were: α-helix (1662–1645 cm^−1^), β-sheet (1637–1613 cm^−1^ and 1710–1682 cm^−1^), turn/bend (1682–1662 cm^−1^), and disordered or random (1650–1637 cm^−1^) [34].

The orientation of the helical component of the peptides in surfactant lipid multilayers was determined via polarization of the IR beam using gold wire polarizers (Perkin Elmer, Waltham, MA, USA). By rotation of the polarizer in the light beam from 0° to 90° with respect to the peptide–lipid film, the insertion (or tilt) angle for the peptide helical axis with respect to the normal of the multilayer surface could be calculated from the dichroic ratio R (where R = All/Al = parallel Absorption/perpendicular Absorption) [35]. These determinations are based on the assumption that one is measuring a thick film with the components of the electric field of the evanescent wave assumed to be *E*_x_ = 1.398, *E*_y_ = 1.516, *E*_z_ = 1.625, and an angle α = 39° for the vibrational dipole relative to the molecular axis of the helix and enables an order parameter *S* to be approximated. Using this formalism, the experimental tilt angle, Θ, can then be calculated from this order parameter [36]. This calculated tilt angle should be considered as a maximum estimation since the actual tilt angle might likely be lower.

The orientation of the phospholipid acyl chain axis of the surfactant lipid matrix was confirmed by measuring the average tilt angle deduced from the molecular order parameter (Supplementary File S4). The lipid acyl chain orientation relative to the multilayer surface normal was experimentally estimated using polarized absorption from the contributions of the antisymmetric and symmetric CH_2_ vibrations at 2918 cm^−1^ and 2850 cm^−1^ [37,38] (Supplementary File S4).

The transmembrane topography of canine SP-C peptide α-helical structures was estimated via hydrogen/deuterium (H/D) exchange of the amide II protons monitored using FTIR spectroscopy. The peptide–lipid film spread on the ATR crystal surface was flushed with D_2_O vapor in nitrogen gas. The decay of the amide II band area (1525–1565 cm^−1^) was measured as a function of time (0–6 h) to determine the accessibility of the helical domain in the lipid multilayer [10].

### 2.7. Electron Spin Resonance (ESR) Measurements of Lipid Molecular Order Mediated by Synthetic Surfactant Peptides

Synthetic surfactant dispersions were labeled with 5, 12, and 16 doxyl stearic acid spin probes (Avanti Polar Lipids, Alabaster, AL, USA) by addition of the probe in a minimal volume of ethanol (1 µL stock solution). The ratio of probe to phospholipids was at a mole ratio of 1 doxyl stearic probe to 200 phospholipids to minimize potential probe–probe interactions. Measurements were made with a Bruker EMXPlus X-band ESR spectrometer (Bruker, Billerica, MA, USA) operating at 9.76 GHz. Typical acquisition settings were a scan width of 100 G (Gauss or 10 Telsa) with a midfield line near 3250 G, a modulation amplitude of 0.63 G, and a microwave power of 0.67 Watt. The samples were contained in 50 µL capillaries and the temperature maintained at 37 °C by flowing temperature-regulated nitrogen gas over the sample in the ESR cavity. ESR spectra were accumulated using Bruker Xenon software (https://www.bruker.com/de/products-and-solutions/mr/epr-instruments/epr-software/xenon.html accessed on 12 December 2023).

The ESR spectral data analysis for the molecular order parameter S that is a measure of membrane “fluidity” or microviscosity was based on the formalism of Gaffney [39].

### 2.8. Captive Bubble Surfactometry

Adsorption and surface tension-lowering ability of six liquid surfactant formulations (SP-Cff ion-lock ± B-YL, SP-Csf ion-lock ± B-YL, B-YL and Curosurf) were performed on a captive bubble surfactometer described by Schurch and co-workers [40,41,42]. The leak-proof bubble chamber is filled with Goerke’s buffer with 10% sucrose, followed by insertion of 2 µL (1 µL for Curosurf as it contains 80 mg/mL of lipids) of surfactant that floats against an 1% agarose gel plug and an air bubble. Initial adsorption of the surfactant to the bubble’s air–liquid interface is measured, whereafter the bubble chamber is sealed, the air bubble is expanded, and post-expansion adsorption measured.

Quasi-static compression and expansion of the air bubble involves a series of 5% alterations in bubble volume at 10 s intervals, when the surface film can relax, during 4 cycles. Quasi-static cycling is followed by dynamic compression and expansion with cycling between volumes of 10% and 110% of the original bubble area at a rate of 20 cycles/min that mimics physiologic breathing. In surface-active surfactant formulations, quasi-static and dynamic cycling both result in significant flattening of the air bubble [43]. Continuous video recording of the bubble shape allows for analysis of surface tension with custom-designed software [44,45]. Optimal surfactant formulations reach a minimum surface tension of <5 mN/m. All measurements were performed in at least quadruplicate.

## 3. Results

### 3.1. Secondary Structure Predictions of Canine SP-C Peptide Mimics

**Figure 2:**
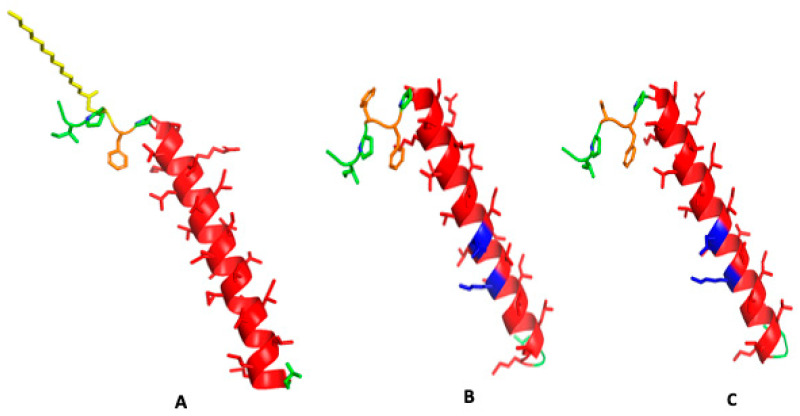
Secondary structure predictions for canine SP-C protein variant amino acid sequences. (**A**) Native canine SP-C protein with cysteine thioester-linked palmitic acid at residue 4 highlighted in yellow with phenylalanine in orange at position 4, disordered N-terminus in green and hydrophobic alpha helical transmembrane sequence in red (Supplementary File S1). (**B**) Canine SP-Cff ion-lock peptide with phenylalanine substituted for Cys-palmitic acid at amino acid position 4 in orange and ion-lock residue ion-lock pair E20/K24 in blue highlight at residues 20–24 in the hydrophobic polyvaline alpha helical amino acid sequence in red (Supplementary File S2). (**C**) Canine SP-Csf ion-lock peptide with serine substituted for Cys-palmitic acid at amino acid position 4 in orange, and ion-lock residue ion-lock pair E20/K24 in blue highlight at residues 20–24 in the hydrophobic polyvaline alpha helical amino acid sequence in red (Supplementary File S3).

The known sequence of canine SP-C (Figure 1) was used as a template for the design of SP-C variants for better understanding the structure and function of the various domains of the protein in surfactant lipids. AI-based secondary structural prediction of the native protein amino acid sequence (Figure 2A) indicates that the N-terminal domain has more of an unstructured turn conformation with the cystine thioester-linked palmitic acid projecting away from the protein backbone, whereas the vicinal phenylalanine has the opposite orientation and lies parallel to mid and C-terminal backbone structure. The hydrophobic mid and C-terminal sequences are predicted as having an alpha helical secondary conformation and closely resemble the bovine NMR structure (PDB accession code: 1SPF) [12].

The canine SP-Cff ion-lock peptide (Figure 1B) was designed to test the importance of residue four in the N-terminal sequence of the protein for optimal surface activity. In this version of the canine SP-C, phenylalanine was used as a surrogate cysteine-palmitate in the native sequence and a glutamic acid-lysine ion-lock was placed at sequence positions 20–24 of the hydrophobic helical domain to minimize possible beta sheet amyloid propensity [10]. The predicted secondary conformation of this variant (Figure 2B) is very similar to that of the native peptide structure shown in Figure 2A.

A second canine SP-C peptide variant (SP-Csf ion-lock) was synthesized to better characterize the function of the N-terminal vicinal residues at amino acid positions 4 and 5. In this variant (Figure 1C) the cysteine-palmitate thioester at position 4 was replaced with the non-sulfur surrogate amino acid residue serine, thereby eliminating any potential amino acid side chain hydrophobic interactions with surfactant lipids. The peptide also incorporated the glutamic acid-lysine ion-lock in the hydrophobic mid and C-terminal segments to attenuate amyloid formation. AI-based secondary structure predictions for this SP-C variant (Figure 2C) were like those for both the canine SP-C native and SP-Cff ion-lock peptides, further suggesting that substitution of serine, a polar non-ionic amino acid, for amino acids with hydrophobic side chains had no apparent effect on the secondary structure of the peptide.

While the above AI-based protein structural predictions have enhanced the theoretical estimates of protein secondary and tertiary structure, they have yet to replace experimental methodology with residue-specific protein structure determination such as x-ray crystallography, NMR, and cryo-EM [46,47]. In particular, some problems include that the rotations of the axes for transmembrane helices are not optimized and present AI is limited in describing the active sites where ligands bind [48]. However, the AI structural prediction programs now available certainly have accurate enough structure prediction that can serve as initial starting structures for refinement in relevant simulated molecular environments using molecular dynamics to give a reasonable approximation of the secondary and tertiary conformations of the protein in question.

**Figure 3:**
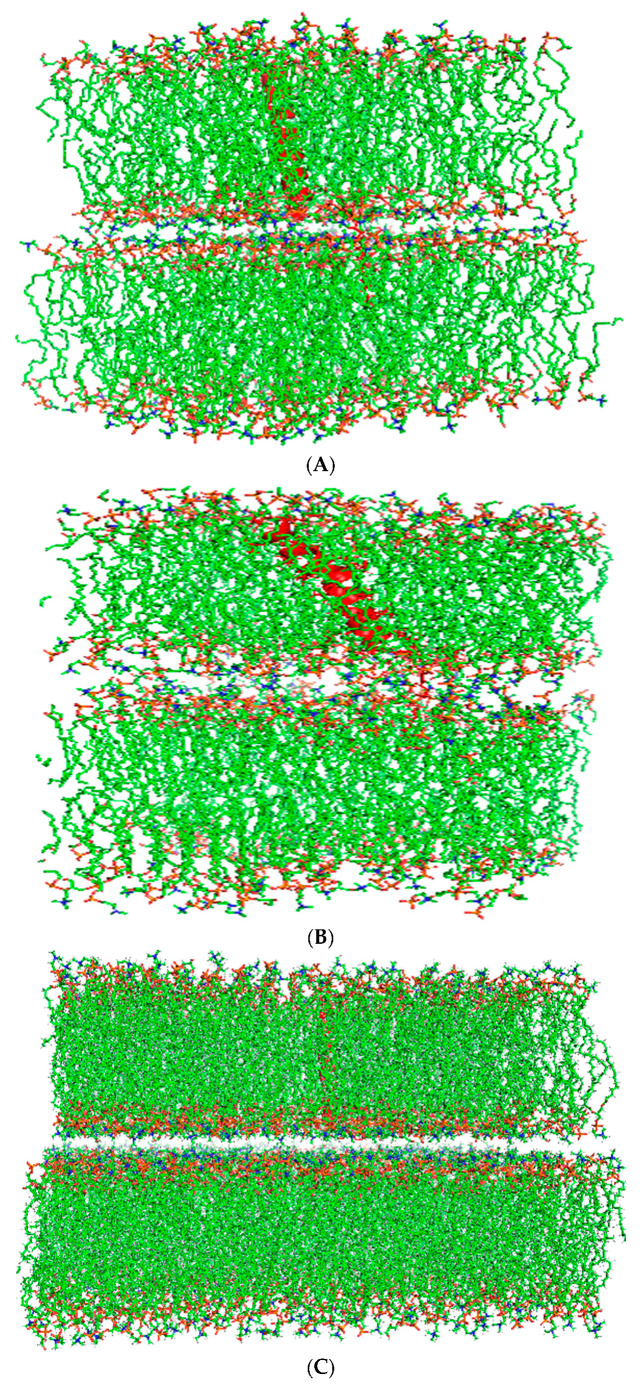

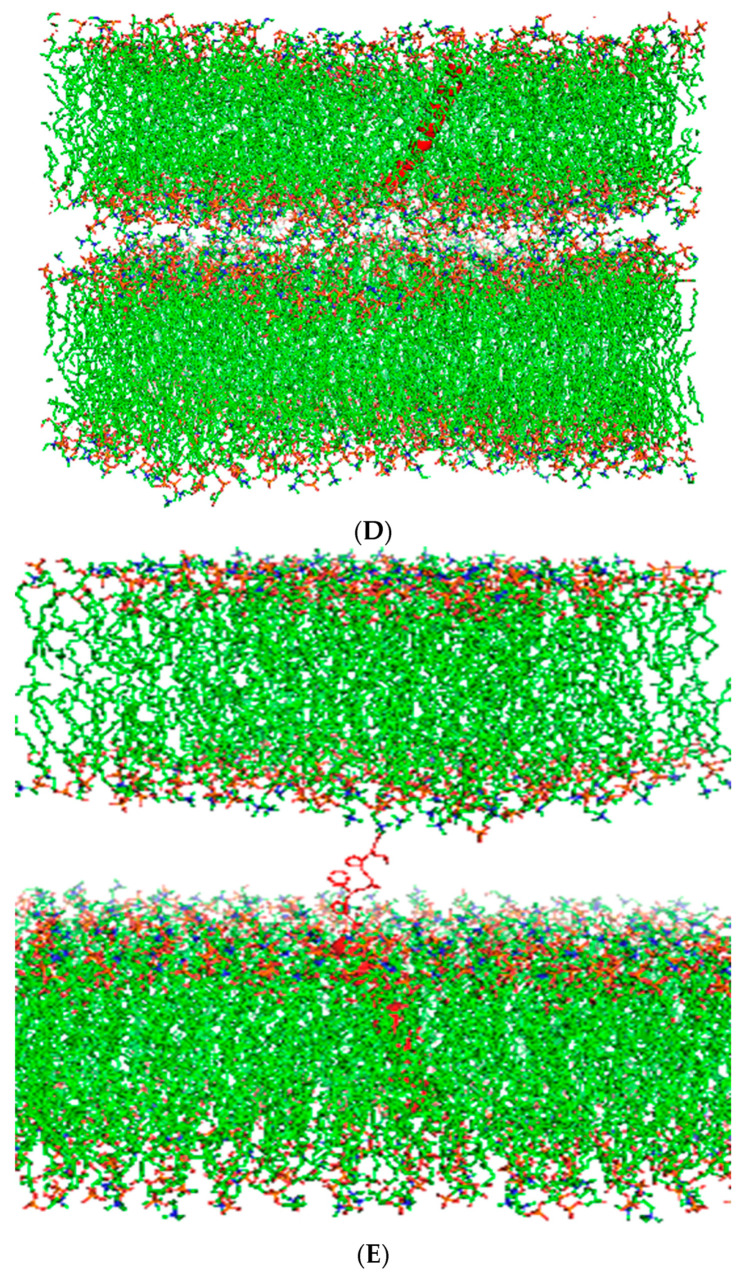

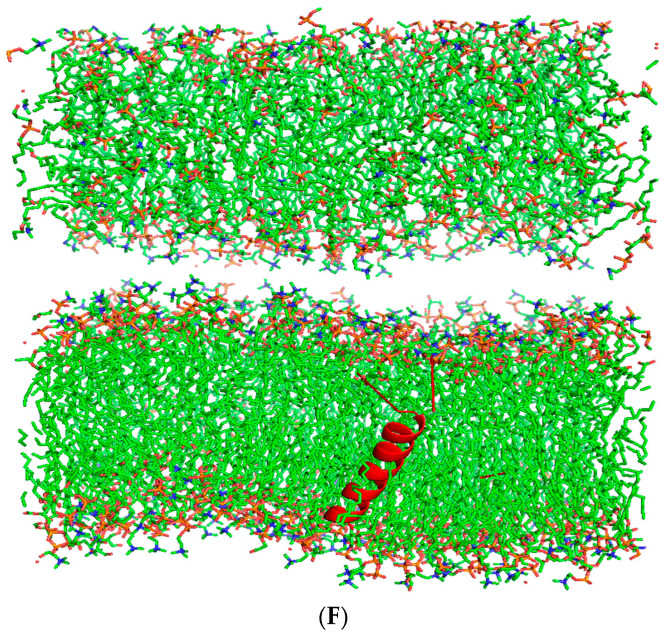
Molecular illustrations for a two-surfactant lipid bilayer simulation system with one SP-Cff ion-lock peptide in a single bilayer adjacent to a second surfactant bilayer without peptide. The approximate distance between the surfaces of the two adjacent bilayers was ten ångström at the start of the simulation. (**A**) Canine SP-C with palmitic acid thioester-linked to cysteine 4 at zero time in red in two adjacent surfactant bilayers. (**B**) Canine SP-C protein in red between two bilayers after 10 nano seconds of molecular dynamics. (**C**) Starting ensemble of the simulation with the canine SP-Cff ion-lock peptide shown in red highlight with their N-terminus oriented toward the opposing bilayer. (**D**) The two adjacent bilayers of synthetic surfactant lipids after 10 nanoseconds of simulation shown with the interaction of the canine SP-Cff ion-lock peptides with the opposing bilayer system. (**E**) Starting ensemble of the simulation of canine SP-Csf ion-lock peptide highlighted in red with the N-terminal domain oriented opposing bilayer. (**F**) Two adjacent bilayers of synthetic surfactant lipids after 10 nanoseconds of simulation.

The preliminary secondary structural predictions for canine SP-Cff ion-lock and SP-Csf ion-lock constructs were then used to gain insights into the impact of these alterations on protein function in simulated surfactant lipid dispersions with molecular dynamics. SP-C is a critical element in the attachment of subphase surfactant lipid multilayers to the opposing lipid monolayer at the air–water interface in the lung [15]. The protein attaches the reservoir of lipids to the monolayer to facilitate the transport of surfactant lipids to and from the surface film during expansion and contraction of the breathing cycle [49]. This function was tested in silico by placing two bilayer surfaces approximately 10 angstroms from one another with one bilayer containing the native canine SP-C with a thioester-linked palmitic acid or the canine SP-C variant peptide and touching the opposing bilayer without peptide (Figure 3). The simulation was then initiated to determine if the presence of the peptide resulted in lipid bilayer–bilayer interaction. As shown in Figure 3B,D, after only 10 nanoseconds of simulation, there was considerable bilayer interaction facilitated by the presence of the palmitoylated native canine SP-C and the canine SP-Cff ion-lock peptide. These MD simulation results suggest that an amino acid residue with a palmitoylated cysteine side chain or hydrophobic aromatic side chain at positions four and five in the canine SP-C protein can be a critical element for optimal interaction with surfactant lipid films. In contrast to the palmitoylated native and SP-Cff ion-lock derivative canine peptides, the SP-Csf ion-lock derivative with a serine residue at sequence position 4 did not show any apparent fusion with the adjacent bilayer (Figure 3F). This observation suggests that SP-C bilayer-bilayer mediated interaction is dependent on the presence of a hydrophobic amino acid side chain in the N-terminal domain of the peptide.

### 3.2. Experimental Determination of the Secondary Structure of Canine SP-C Ion-Lock Peptide Mimics in Micellar Membrane Mimic Dispersions and Surfactant Lipid Films

Both native SP-C protein and SP-Cff peptide mimics have shown strong beta sheet propensities that in time form amyloid-like structures that render surfactant dispersions functionally inactive [50]. The formation of these beta amyloid SP-C structures correlates with the limited shelf life of approximately 12 months for most surfactant dispersion now in clinical use. The replacement of leucine for valine in the hydrophobic poly-valine sequence [51] or the introduction of charged polar residues at selective positions in the sequence that form ion-locks in hydrophobic lipid bilayer environments favor alpha helical secondary structure and minimize the formation of beta sheets that form surface-inactive amyloid structures [10]. Both native SP-C and SP-C mimics have been shown to assume alpha helical axial orientations as trans-bilayer in surfactant lipid dispersions [10,51,52,53].

**Figure 4:**
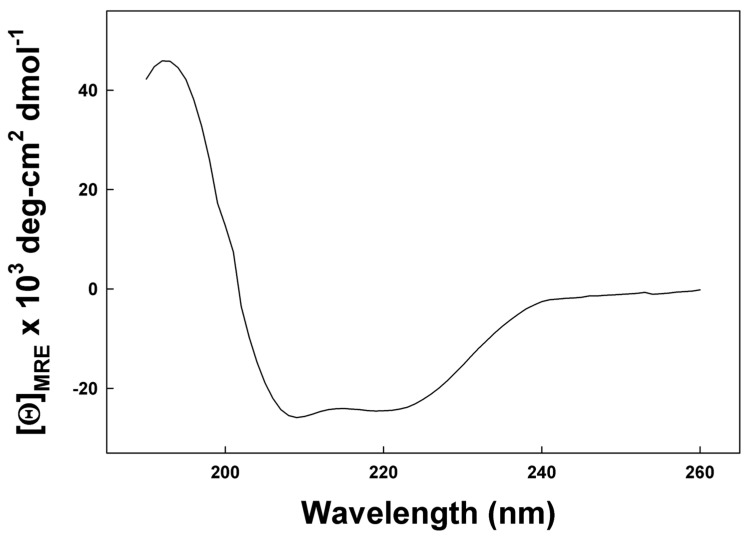
CD spectrum of SP-Cff ion-lock canine peptide in SDS micelles. The detergent–peptide sample was scanned from 190 nm to 260 at a temperature of 37 °C. Detailed Spectral data analysis is available in Supplementary File S1.

Preliminary secondary structural characterization of SP-Cff peptide was determined in the membrane mimic micellar anionic detergent Sodium Dodecyl Sulfate (SDS)–buffer dispersion using circular dichroism spectroscopy (CD). The CD spectrum shown in Figure 4 has definitive spectral minima at 208 nm and 222 nm that are the spectral signature of a peptide assuming dominant alpha helical conformations in this membrane mimic environment. Deconvolution of the spectrum with the program Selcon 3 [29] indicates that the peptide has approximately 68.7% helical structure. This high helical component is consistent with molecular dynamics simulations of the canine SP-C ion-lock in an SDS micelle and confirms the high alpha helical peptide propensity in the detergent dispersion. Additional evidence for the credibility of AI-based structural predictions is provided by using the simulated molecular structure (Supplementary File S1).

**Figure 5:**
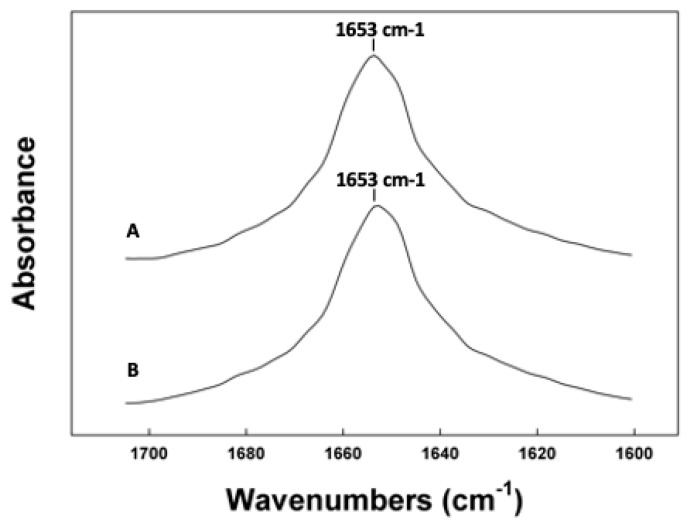
ATR-IR spectra of the amide I region of canine SP-C peptides in synthetic surfactant lipid films. (**A**) Canine SP-Cff ion-lock. (**B**) Canine SP-Csf ion-lock. The FTIR spectra for canine SP-Cff ion-lock and SP-Csf ion-lock peptides in deuterium vapor phosphate buffer (pD 7.4) hydrated lipid–peptide film after one hour of hydration. FTIR spectra for peptide in lipid with phosphate buffer were obtained by subtracting the lipid spectrum hydrated with deuterated buffer from those of peptides in lipid with buffer vapor.

To further validate the in-silico peptide–lipid predictions, the conformation and molecular topography of the canine SP-C variants was measured in surfactant lipid films using ATR-FTIR spectroscopy, a surface-sampling experimental technique permitting analysis of the peptide conformations in lipid without interference from light-scattering artifacts that would be encountered in transmission spectroscopy [52]. Examination of the infrared spectrum of canine SP-Cff ion-lock in synthetic surfactant lipids is shown in Figure 5. The amide I band that is the major conformational spectral region indicative of protein secondary structure has a dominant absorption peak at 1653 cm^−1^, typical for alpha helical secondary structures. There is also some minor absorption around 1643 cm^−1^, suggesting the presence of disordered structures for the peptide in this environment as well as some absorption between 1660 to 1680 cm^−1^ (Table 1). The infrared spectrum of canine SP-Csf ion-lock shown in Figure 5B closely resembles that of canine SP-Cff ion-lock, further suggesting that both variants have dominant alpha helical conformations (Table 1).

The orientation of the dominant conformations of the canine SP-C protein variants was also determined using polarized ATR-FTIR spectroscopy of the peptides in synthetic surfactant lipid films. The helical axis of both canine SP-Cff ion-lock and SP-Csf ion-lock peptides had a trans-bilayer orientation in the film with an angle of insertion of approximately 33° that was close to parallel to that of the phospholipid acyl chain long axis (Table 1 and Supplementary File S4).

**Figure 6:**
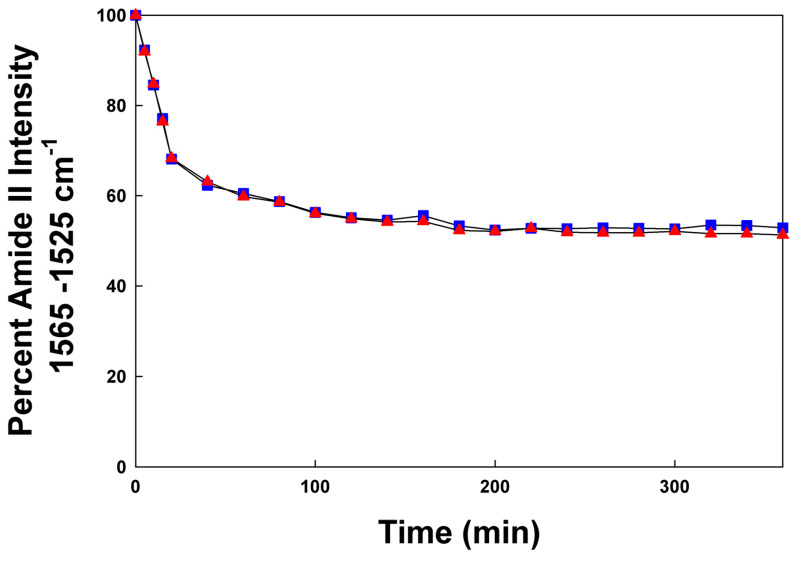
Deuterium/hydrogen exchange kinetic of canine SP-C peptide mimics in synthetic surfactant lipids (DPPC:POPC:POPG, 5:3:2 weight ratio). Canine SP-Cff ion-lock (red triangles) and canine SP-Csf ion-lock (blue squares) incorporated into synthetic surfactant lipids after the peptide–lipid sample on the ATR crystal was hydrated with phosphate buffer vapor (pD 7.4) at 37 °C. The time-dependent H/D exchange of the amide groups was determined from the decay of the amide II band as a function of time (0–6 h).

Further insight into the molecular topography of the canine SP-Cff ion-lock was revealed via hydrogen–deuterium exchange that can be monitored using the amplitude of the amide II band peak center at ~1543 cm^−1^. One of the signatures of the extent of peptide and protein insertion into bilayer structures can be inferred by observing the extent of hydrogen–deuterium exchange (H/D) [10,52] of the amide protons as a function of time. Peptide amino acid residues that are buried in the interior or trans-bilayer domain of the membrane-like ensemble tend to exchange protons with bulk aqueous environment at a much slower rate than those residues near the aqueous–lipid interface. The H/D exchange for both canine SP-Cff ion-lock and SP-Csf ion-lock peptides is shown in Figure 6. Both SP-C ion-lock mimic peptides had a similar biphasic time course of H/D exchange in surfactant lipid films. There was an initial rapid exchange of protons in the first 20 min followed by a very slow phase that continued for at least 6 h. This biphasic H/D exchange is typical of trans-bilayer alpha helical proteins that have most of the structure buried in the hydrophobic mid-section of the bilayer that is inaccessible to the aqueous interface [10]. Similar H/D exchange kinetics have been reported for native bovine and porcine SP-C in surfactant lipid films [52,53], suggesting that the canine SP-C ion-lock peptide mimics assume orientations in surfactant lipid ensembles like that of the native proteins.

### 3.3. Changes in Lipid Molecular Order Induced by Synthetic Canine SP-C Ion-Lock Peptides

Although fatty acid doxyl spin labels cannot characterize directly the formation of non-bilayer structures in lipid dispersions or the occurrence of motional restricted or so-called boundary lipids directly adjacent the protein-bulk lipid environment, they are very sensitive probes of the overall bilayer acyl chain molecular order as a function of depth in the ensemble [54]. Since the Electron Spin Resonance nitroxide spectrum of the fatty acid derivative is not an optical experimental approach, there are no light-scattering artifacts and reports the angle of amplitude of motion for a very specific bilayer domain. This approach permits detection of peptide induced changes in the molecular order of lipids near the lipid–bulk interface of the lipid membrane as well as those deeper in the bilayer. Unlike FTIR based hydrogen–deuterium exchange measurements which only infer the effects of peptide insertion into the lipid, ESR spin probes allow characterization of the degree and specific trans-bilayer depth of a given peptide’s influence on a surfactant lipid bilayer system’s molecular order. The use ESR nitroxide labelled fatty acyl chain lipids has been shown to be an excellent approach for the detection of SP-C-induced changes in surfactant lipid dispersions [55].

**Figure 7:**
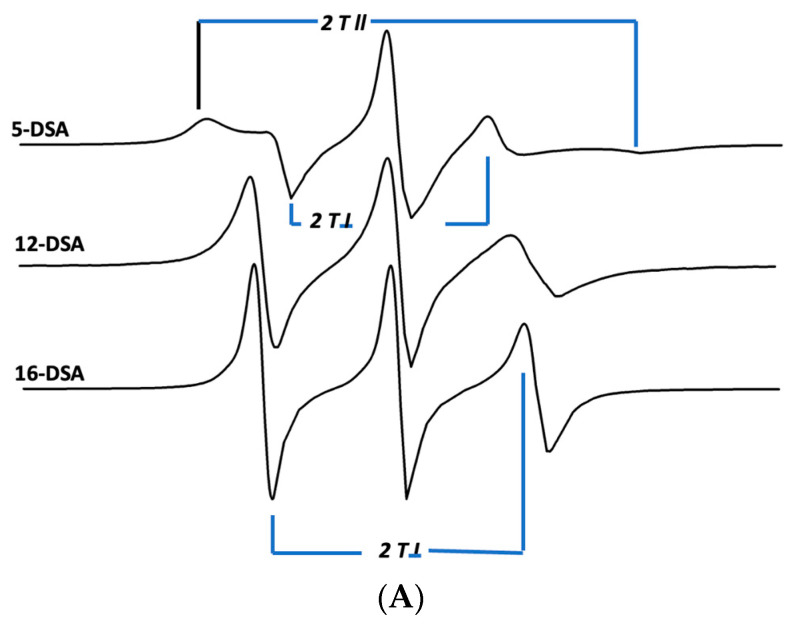

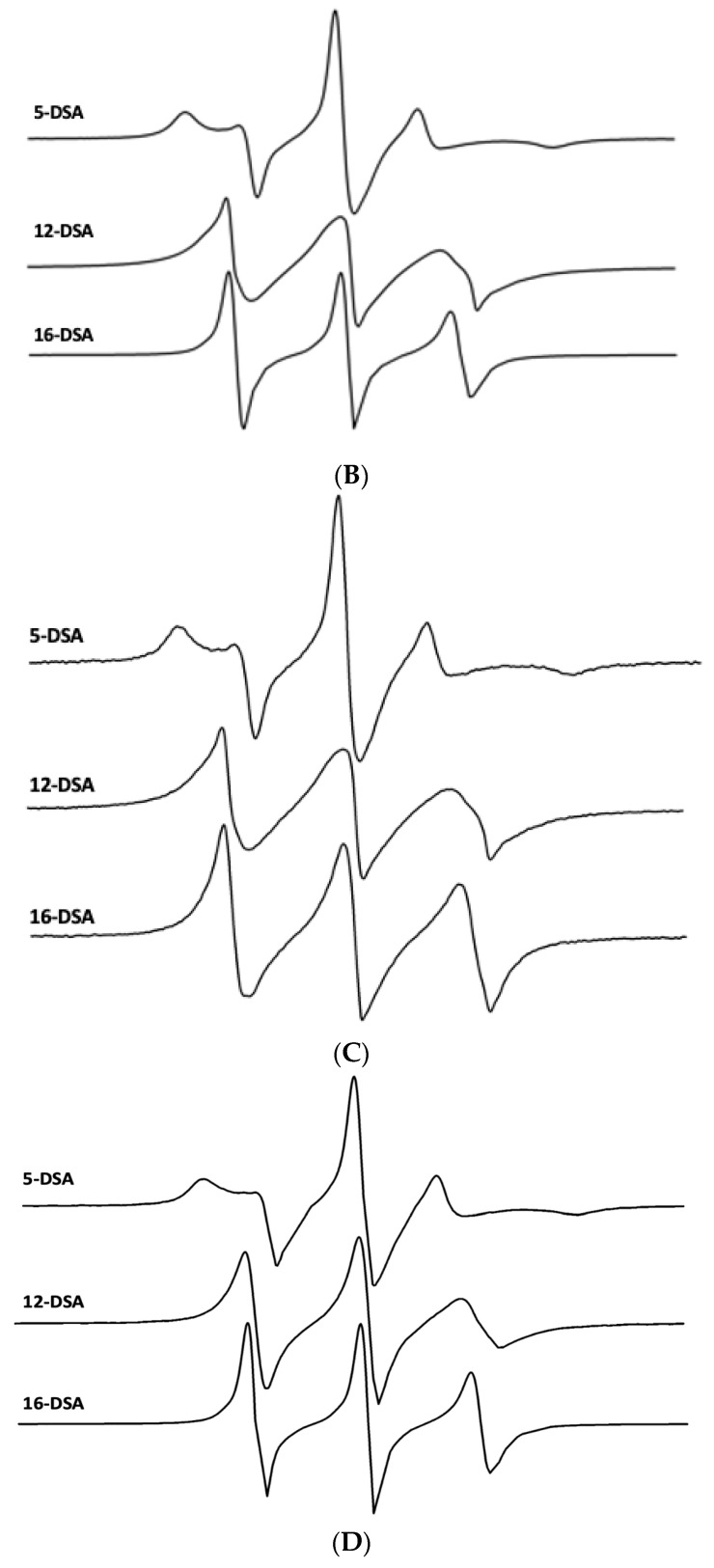
ESR spectra of doxyl stearic acid (DSA) spin labels in synthetic lung surfactant liposomal dispersions. Spectral parameters at 37 °C for 5-DSA, 12-DSA, and 16-DSA in synthetic surfactant lipids are indicated as parallel (2T_ll_) and perpendicular (2T_l_) hyperfine components. The lipid to probe ratio was 200:1, mole:mole. Spectral scan from 3320 to 3400 Gauss. (**A**) Synthetic surfactant liposomes alone. (**B**) Synthetic surfactant liposomes with 2% canine SP-Cff ion-lock peptide. (**C**) Synthetic surfactant liposomes with 2% canine SP-Csf ion-lock peptide. (**D**) Synthetic surfactant liposomes with 4% B-YL peptide and 2% canine SP-C ion-lock peptide.

The influence of hydrophobic surfactant peptides on the bilayer fluidity or microviscosity in surfactant lipids was investigated by determining the molecular order of nitroxide fatty acid spin labels in the lipid–peptide ensembles. The influence of the canine SP-Cff ion-lock peptide near the phospholipid polar head groups was characterized by incorporating a 5-doxyl stearic acid spin label into the surfactant dispersions, which enhanced the lipid ordering in this bilayer domain compared with surfactant lipids without peptide (Figure 7A and Table 2). Addition of the B-YL peptide to the canine SP-Cff ion-lock surfactant lipid dispersion increased the bilayer microviscosity even more than with canine SP-Cff ion-lock and lipids alone (Figure 7B and Table 2). Sampling the middle trans-bilayer domain of the surfactant lipid bilayer with the 12-doxyl stearic acid spin label probe indicated that the canine SP-Cff ion-lock also increases the lipid acyl chain molecular order like that observed near the polar head group region of the bilayer (Figure 7B and Table 2). Similar changes in the acyl chain lipid molecular order can also be seen for SP-Csf, further indicating that the hydrophobic alpha helical domain of the SP-C ion-lock variants induce lipid ordering in the same trans-bilayer fashion (Figure 7C and Table 2). However, the addition of the B-YL peptide to the dispersion had no effect on the molecular order near the twelfth carbon of the phospholipid acyl chains near the middle of the bilayer, suggesting that the SP-B peptide mimic interacts with the dispersion at the aqueous–bilayer interface domain. Measurements of the trans-bilayer domain near the phospholipid acyl chain terminus in the middle of the ensemble with 16-doxyl stearic acid shows that canine SP-Cff ion-lock also increases the molecular order as observed at the other depths of the surfactant lipid bilayer (Figure 7D and Table 2). The inclusion of the B-YL peptide in the formulation had no effect on the middle of the bilayer, further suggesting that the influence of the SP-B peptide mimic on the bilayer fluidity was primarily at the lipid–bulk aqueous surface of the multilayer ensemble. These depth dependent bilayer molecular ordering effects are consistent with the predicted topographical orientations, peptide backbone polarized FTIR measurements, and deuterium exchange signatures for canine SP-Cff ion-lock peptides in surfactant lipid bilayers. These observations are consistent with canine SP-Cff ion-lock variants assuming alpha helical conformations of hydrophobic trans-bilayer peptides, whereas B-YL is an amphipathic surface seeking helix hairpin that is at the liposomal–aqueous interfacial domain (Figure 7).

### 3.4. In Vitro Adsorption and Quasi-Static and Dynamic Surface Activity

Synthetic surfactant preparations were formulated by mixing 35 mg/mL of synthetic surfactant lipids, consisting of DPPC:POPC:POPG 5:3:2 by weight ratio, with canine SP-Cff ion-lock 4%, canine SP-Cff ion-lock 2% + B-YL 4%, canine SP-Csf ion-lock 4%, canine SP-Csf ion-lock 2% + B-YL 4%, and B-YL 4%. The clinical surfactant Curosurf^®^ (Poractant Alfa, Chiesi Farmaceutici, Parma, Italy), a porcine lung surfactant extract that contains both native surfactant proteins B and C, was used as a positive control.

**Figure 8:**
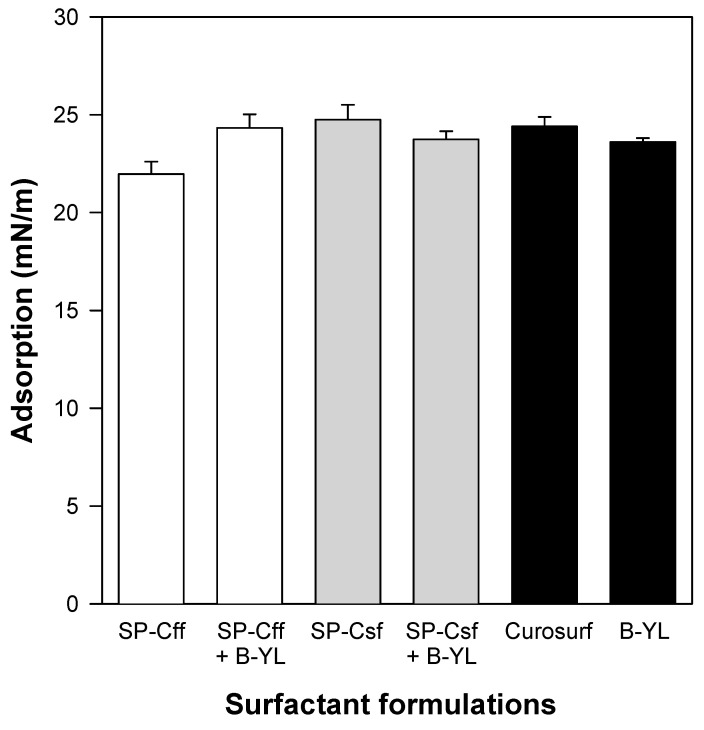
Post-expansion adsorption of six surfactant formulations. Canine SP-Cff ion-lock 4%, SP-Cff ion-lock 2% + B-YL 4%, SP-Csf ion-lock 4%, SP-Csf ion-lock 2% + B-YL 4%, and B-YL 4% were mixed in 35 mg/mL of surfactant lipids consisting of DPPC:POPC:POPG 5:3:2 (wt:wt:wt). Curosurf^®^ contains both porcine surfactant proteins B and C and 80 mg/mL of lipids. Adsorption is expressed as surface tension in mN/m. Values are the average of at least four separate experiments + SEM.

**Figure 9:**
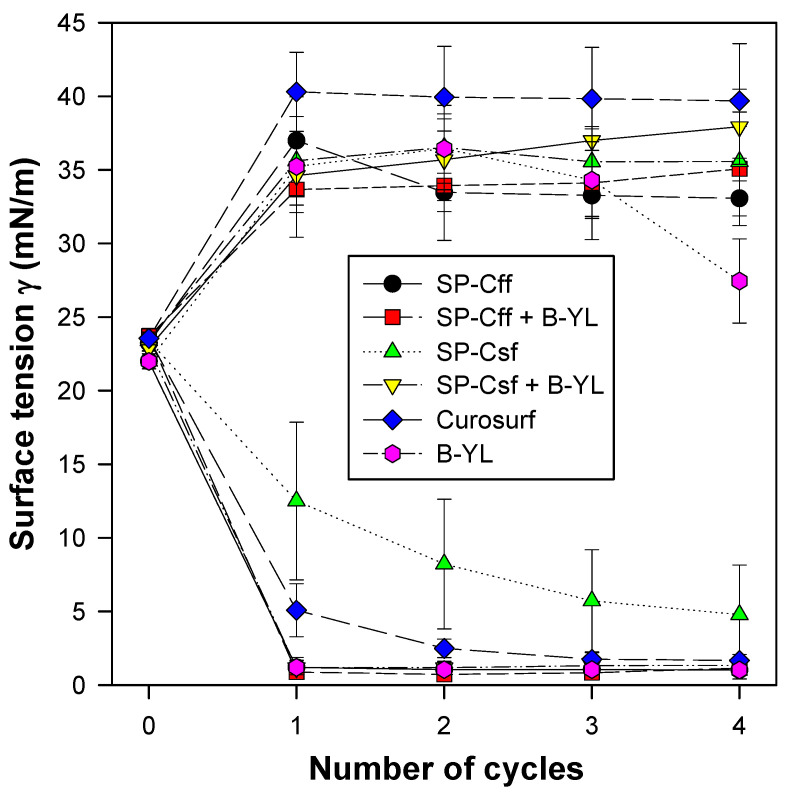
Quasi-static cycling data of six surfactant formulations at captive bubble surfactometry. Canine SP-Cff 4%, SP-Cff 2% + B-YL 4%, SP-Csf 4%, SP-Csf 2% + B-YL 4%, and B-YL 4% were mixed in 35 mg/mL of surfactant lipids consisting of DPPC:POPC:POPG 5:3:2 (wt:wt:wt). Curosurf^®^ contains both native surfactant proteins B and C. The lower lines depict minimum surface tension and the upper lines maximum surface tension at quasi-static cycles 1 to 4. Values are the average of at least four separate experiments + SEM.

**Figure 10:**
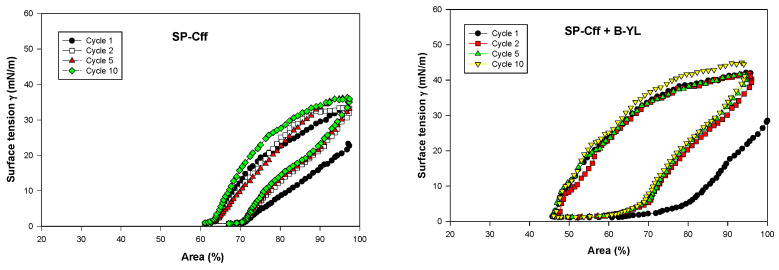

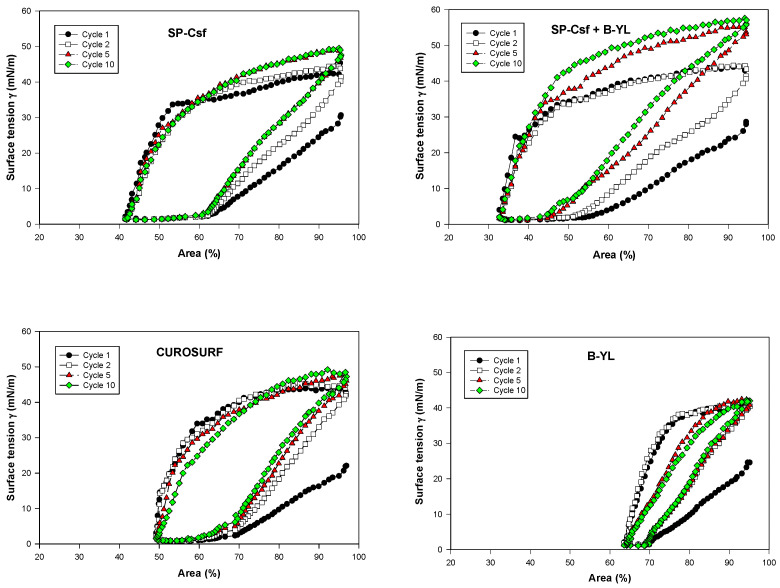
Area compression (%) necessary to reach a low surface tension during dynamic cycling at captive bubble surfactometry. Canine SP-Cff ion-lock 4%, SP-Cff ion-lock 2% + B-YL 4%, SP-Csf ion-lock 4%, SP-Csf ion-lock 2% + B-YL 4%, and B-YL 4% were mixed in 35 mg/mL of surfactant lipids consisting of DPPC:POPC:POPG 5:3:2 (wt:wt:wt). Curosurf^®^ contains both surfactant proteins B and C. The graphs depict the 1st, 2nd, 5th, and 10th cycle of each of the six surfactant formulations. Values are the average of at least four separate experiments.

Post-expansion adsorption values varied between 22 and 25 mN/m and were comparable among the six surfactants (Figure 8). Quasi-static cycling experiments (Figure 9) indicate that SP-Csf ion-lock 4% was less surfaceactive than SP-Cff ion-lock 4% during the first four quasi-static cycles and that all formulations except SP-Csf ion-lock 4% reached minimum surface tension values << 5 mN/m. Dynamic cycling experiments (Figure 10) provide insight into hysteresis and the area of compression necessary to reach a low surface tension value (here defined as <5 mN/m). SP-Cff ion-lock 4%, SP-Cff ion-lock 2% + B-YL 4%, Curosurf, and B-YL 4% needed a smaller area of compression to be surface-active than SP-Csf ion-lock 4% and SP-Csf ion-lock 2% + B-YL 4% (Figure 10). Among the four surfactant formulations with a canine SP-Cff or SP-Csf ion-lock peptide, SP-Cff 2% with B-YL 4% was the most surface-active combination and most comparable to the Curosurf control.

## 4. Discussion

The proteomic molecular design of SP-C peptide mimics has focused on the development of macromolecules that emulate the structure and function of native SP-C protein. Because SP-C is a lipo-protein, thesynthesis and expression of the protein presents challenges regarding palmitoylation of the N-terminal domain which stabilizes the dominant alpha helical structure of the transmembrane hydrophobic sequence and is critical for optimal function in surfactant lipids [56]. Lung surfactant monolayer–multilayer interactions are some of the most important functions of SP-C in lung surfactant. The molecular mechanism of this interaction has been studied in detail via monolayer fluorescent imaging and scanning-force microscopy (SFM) [15,56]. These studies suggest that one of the key roles of the SP-C protein in surfactant lipid ensembles is to mediate the surface pressure-dependent, fully reversible exclusion of multilayered lipid protrusions as part of the surface monolayer continuum. Additional SFM studies of SP-C peptides with phenylalanine residues substituted for covalently linked palmitate also emulate a similar mechanism of action as observed with the SP-C parent protein [16,57]. The amino acid sequence of canine SP-C is an attractive template for design of SP-C peptide mimics since the N-terminal domain already has a phenylalanine amino acid residue at position 4 that is a surrogate for one of the vicinal cysteine-palmitate residues [17]. However, both native deacylated SP-Cs and surrogate SP-C peptides with vicinal phenylalanines substituted for thioester linked palmitate have limited shelf-lives in surfactant lipids and tend to form inactive beta sheet structures [56]. These high amyloid propensities of the poly-valine sequence have been minimized through the substitution of leucine for valines [58] or the mutation of specific residues in the hydrophobic sequence with ion pairs that stabilize a helical conformation in hydrophobic environments [10].

SP-C also has a major role in the molecular ordering of surfactant lipids in a manner that alters mono and multilayer structure and suggests enhanced lung surfactant interfacial functionality by altering lipid interdigitation [55]. The possible mechanism of action of SP-C mediated changes in a lipid structure has been shown by electron spin resonance measurements of surfactant dispersions labeled with doxyl-stearic acid. By examining the protein-induced changes in surfactant lipid molecular motion using a suite of fatty acid spin labels at various positions in the probe acyl chain, changes in the surfactant phospholipid molecular motion were determined as function of protein concentration. These spin label studies indicate that the presence of SP-C in surfactant lipids enhances the trans-bilayer average acyl chain molecular order.

In the present studies with the canine SP-Cff ion-lock construct, doxyl-stearic acid spin label measurements clearly show that the presence of the peptide in synthetic surfactant lipids increases the trans-bilayer average molecular order of the phospholipid acyl chains. In contrast to the canine SP-Cff ion-lock peptide, the SP-B peptide mimic B-YL increases the phospholipid molecular order near the surface of the liposomal–bulk aqueous interface, but did not have any effect on the mid-lipid bilayer domain of the multilamellar vesicles observed for amphipathic alpha helical SP-B sequences.

At captive bubble surfactometry, adsorption of both canine SP-C ion-lock peptides mixed in surfactant lipids with or without the SP-B peptide mimic B-YL was similar to B-YL alone and to the clinical porcine surfactant Curosurf that contains both native SP-B and SP-C and is one of the leading animal-derived surfactants used in the treatment of premature infants with neonatal respiratory distress syndrome due to surfactant deficiency as a result of lung immaturity. Captive bubble surfactometry of canine SP-Cff and SP-Csf ion-lock peptides mixed in surfactant lipids showed relatively low quasi-static and dynamic surface activity in the absence of the SP-B peptide mimic B-YL. Mixed with B-YL canine SP-Cff ion-lock performed clearly better than canine SP-Csf ion-lock. These in vitro functional data are in line with the structural findings obtained with FTIR.

In future studies we plan to determine the high-resolution residue specific peptide secondary structure in surfactant lipid dispersions with solid state NMR or cryo-EM methodology.

## 5. Conclusions

AI modeling, molecular dynamics, circular dichroism spectroscopy, Fourier Transform InfraRed spectroscopy, and electron spin resonance studies showed that the secondary structure of canine SP-Cff ion-lock peptide was like that of native SP-C, suggesting that substitution of phenylalanine for cysteine has no apparent effect on the secondary structure of the peptide. Captive bubble surfactometry demonstrated higher surface activity for canine SP-Cff ion-lock peptide in combination with B-YL in surfactant lipids than with canine SP-Csf ion-lock peptide. These studies demonstrate the potential of canine SP-Cff ion-lock peptide to enhance the functionality of the SP-B peptide mimic B-YL in synthetic surfactant lipids.

## Figures and Tables

**Table 1:** Proportions of secondary structures for SP-C peptide mimics in synthetic surfactant lipid multilayers (DPPC:POPC:POPG, 5:3:2, wt%).

Sample	% Conformation				
	α helix	β sheet	Turn/Loop	Disordered	Helix Tilt°
Canine SP-Cff ion-lock	73.2	12.1	7.9	6.8	34.3
Canine SP-Csf ion-lock	72.3	8.2	7.6	11.9	33.8

Secondary structure contribution was estimated from deconvolution of the ATR-FTIR spectra of the peptide amide I band (Figure 2), as described in the Methods section. Data are means of three separate determinations and have a SD ± 5% or better. Helical axis tilt angle is in degrees and has a SD ± 1.7°.

**Table 2:** Doxyl stearic acid spin label order parameter S in surfactant lipid–peptide dispersions as a function of bilayer depth.

**(A)**			
Sample 5 doxyl-stearate	2A_II_	2A_l_	S
Surfactant Lipids	46.0 G	21.0 G	0.547
Surfactant Lipids 2% SP-Cff ion-lock	48.0 G	20.8 G	0.595
Surfactant Lipids 2% SP-Csf ion-lock	47.8 G	20.7 G	0.593
Surfactant Lipids 4% BYL + 2% SP-Cff ion-lock	48.3 G	20.9 G	0.602
**(B)**			
Sample 12 doxyl-stearate	2A_II_	2A_l_	S
Surfactant Lipids		25.2 G	0.564
Surfactant Lipids 2% SP-Cff ion-lock		24.7 G	0.582
Surfactant Lipids 2% SP-Cfs ion-lock		24.3 G	0.597
Surfactant Lipids 4% BYL + 2% SP-Cff ion-lock		24.7 G	0.582
**(C)**			
Sample 16 doxyl-stearate	2A_II_	2A_l_	S
Surfactant Lipids		26.9 G	0.501
Surfactant Lipids 2% SP-Cff ion-lock		26.2 G	0.527
Surfactant Lipids 2% SP-Csf ion-lock		25.9 G	0.538
Surfactant Lipids 4% BYL + 2% SP-Cff ion-lock		26.0 G	0.557

## Data Availability

Data are contained within the article and supplementary materials.

## Supplementary Materials

The following supporting information can be downloaded at: https://www.mdpi.com/article/10.3390/biomedicines12010163/s1. File S1: Secondary Structure Model of Canine SP-C protein using AlphaFold prediction program and Analysis of CD spectra of canine SP-Cff ion-lock peptide in SDS micelles. File S2: Secondary Structure Model of Canine SP-Cff ion-lock protein using AlphaFold prediction program. File S3: Secondary Structure Model of Canine SP-Csf ion-lock protein using AlphaFold prediction program. File S4: FTIR Determination of peptide conformation and phospholipid acyl chain orientation in surfactant lipid multilayers. References [59,60] are cited in the supplementary materials.
