# Supplementary material for: Structure and Function of Canine SP-C Mimic Proteins in Synthetic Surfactant Lipid Dispersions
Source: Biomedicines. 2024 Jan 12;12(1):163. doi: 10.3390/biomedicines12010163 (PMC10813813; doi:10.3390/biomedicines12010163)
Supplement: Supplementary file 1 [file biomedicines-12-00163-s001.zip › S1.pdf]

S1 – Secondary Structure Model of Canine SP-C protein using AlphaFold prediction program and Analysis of CD spectra of Canine SP-Cff ion-lock peptide in SDS Micelles.

Article Title: Structure and Function of Canine SP-C Mimic Proteins in Synthetic Surfactant Lipid Dispersions

Frans J. Walther[1,2,*] & Alan J. Waring[1,3]

[1] Lundquist Institute for Biomedical Innovation at Harbor-UCLA Medical Center
1124 West Carson Street
Torrance, CA, USA

[2] Department of Pediatrics
David Geffen School of Medicine
University of California Los Angeles
405 Hilgard Avenue
Los Angeles, CA, USA

[3] Department of Medicine
David Geffen School of Medicine
University of California Los Angeles
405 Hilgard Avenue
Los Angeles, CA, USA

Modeling Protocol

Canine SP-C amino acid sequence downloaded from: https://www.uniprot.org Deposition file: P22397 · PSPC_CANLF. Secondary structure modeling SP-C canine amino acid sequence monomer (Canis lupus familiars) using primary amino acid sequence with the AI based secondary structure prediction program AlphaFold. Structure refinement to the thioester palmitate derivative of cysteine using Charmm GUI (http://www.charmm-gui.org/?doc=input/membrane.bilayer).

Modeling canine SP-C amino acid sequence monomer using primary amino acid sequence with the AI based secondary structure prediction program AlphaFold. The AlphaFold program was run through the Chimera X (version 1.6.1) molecular modeling environment at https://www.cgl.ucsf.edu/chimera/docs/relnotes.html.

Jumper J, Evans R, Pritzel A, et al. Highly accurate protein structure prediction with AlphaFold. *Nature*. 2021;596(7873):583-589. doi:10.1038/s41586-021-03819-2

Mirdita M, Schütze K, Moriwaki Y, Heo L, Ovchinnikov S, Steinegger M. ColabFold: making protein folding accessible to all. *Nat Methods*. 2022;19(6):679-682. doi:10.1038/s41592-022-01488-1

Input amino acid sequence for Chimera X:

>SPC_palF_native_dog

GIPCFPSSLKRLLIIVVVIVLVVVVIVGALLMGL

Command Sequence for AlphaFold Prediction of SP-C dog amino acid sequence:

ChimeraX > Structure Prediction > AlphaFold > paste amino acid sequence > Predict

AlphaFold output for Canine SP-C Atomic Coordinate Data Predicted for Canine SP-C Monomer Structure in PDB format:

File: SPC_PalF_native_dog.pdb

```
ATOM      1  N   GLY A   1      64.480  38.190  90.880  1.00  0.00            N
ATOM      2  HT1 GLY A   1      64.860  37.970  89.940  1.00  0.00            H
ATOM      3  HT2 GLY A   1      63.450  38.370  90.860  1.00  0.00            H
ATOM      4  HT3 GLY A   1      64.660  37.420  91.550  1.00  0.00            H
ATOM      5  CA  GLY A   1      65.150  39.390  91.440  1.00  0.00            C
ATOM      6  HA1 GLY A   1      64.750  40.220  90.870  1.00  0.00            H
ATOM      7  HA2 GLY A   1      66.170  39.120  91.230  1.00  0.00            H
ATOM      8  C   GLY A   1      64.940  39.580  92.920  1.00  0.00            C
ATOM      9  O   GLY A   1      63.820  39.430  93.400  1.00  0.00            O
ATOM     10  N   ILE A   2      65.990  39.890  93.710  1.00  0.00            N
ATOM     11  HN  ILE A   2      66.910  39.820  93.340  1.00  0.00            H
ATOM     12  CA  ILE A   2      65.740  40.370  95.080  1.00  0.00            C
ATOM     13  HA  ILE A   2      64.970  39.750  95.530  1.00  0.00            H
ATOM     14  CB  ILE A   2      66.960  40.110  96.020  1.00  0.00            C
ATOM     15  HB  ILE A   2      67.730  40.820  95.640  1.00  0.00            H
ATOM     16  CG2 ILE A   2      66.710  40.420  97.400  1.00  0.00            C
ATOM     17 HG21 ILE A   2      65.890  39.830  97.860  1.00  0.00            H
ATOM     18 HG22 ILE A   2      66.380  41.470  97.510  1.00  0.00            H
ATOM     19 HG23 ILE A   2      67.670  40.310  97.950  1.00  0.00            H
ATOM     20  CG1 ILE A   2      67.500  38.610  95.910  1.00  0.00            C
ATOM     21 HG11 ILE A   2      68.410  38.590  96.530  1.00  0.00            H
ATOM     22 HG12 ILE A   2      67.810  38.380  94.870  1.00  0.00            H
ATOM     23  CD  ILE A   2      66.520  37.450  96.250  1.00  0.00            C
ATOM     24  HD1 ILE A   2      66.360  37.540  97.350  1.00  0.00            H
ATOM     25  HD2 ILE A   2      66.950  36.460  96.000  1.00  0.00            H
ATOM     26  HD3 ILE A   2      65.490  37.520  95.820  1.00  0.00            H
ATOM     27  C   ILE A   2      65.260  41.920  95.050  1.00  0.00            C
ATOM     28  O   ILE A   2      65.880  42.690  94.330  1.00  0.00            O
ATOM     29  N   PRO A   3      64.130  42.420  95.630  1.00  0.00            N
ATOM     30  CD  PRO A   3      63.160  41.610  96.410  1.00  0.00            C
ATOM     31  HD1 PRO A   3      63.490  41.660  97.470  1.00  0.00            H
ATOM     32  HD2 PRO A   3      63.170  40.600  95.950  1.00  0.00            H
ATOM     33  CA  PRO A   3      63.810  43.760  95.530  1.00  0.00            C
ATOM     34  HA  PRO A   3      63.720  44.110  94.510  1.00  0.00            H
ATOM     35  CB  PRO A   3      62.450  43.890  96.290  1.00  0.00            C
ATOM     36  HB1 PRO A   3      61.770  44.680  95.900  1.00  0.00            H
ATOM     37  HB2 PRO A   3      62.710  44.130  97.350  1.00  0.00            H
ATOM     38  CG  PRO A   3      61.920  42.510  96.190  1.00  0.00            C
ATOM     39  HG1 PRO A   3      61.120  42.400  96.960  1.00  0.00            H
ATOM     40  HG2 PRO A   3      61.460  42.320  95.200  1.00  0.00            H
ATOM     41  C   PRO A   3      64.790  44.770  96.110  1.00  0.00            C
ATOM     42  O   PRO A   3      65.350  44.530  97.180  1.00  0.00            O
ATOM     43  N   CYSPA   4      65.130  45.870  95.450  1.00  0.00            N
ATOM     44  HN  CYSPA   4      64.830  45.910  94.500  1.00  0.00            H
ATOM     45  CA  CYSPA   4      65.880  46.970  96.120  1.00  0.00            C
ATOM     46  HA  CYSPA   4      66.790  46.510  96.480  1.00  0.00            H
ATOM     47  C   CYSPA   4      65.230  47.740  97.300  1.00  0.00            C
ATOM     48  O   CYSPA   4      64.040  47.920  97.350  1.00  0.00            O
ATOM     49  CB  CYSPA   4      66.340  48.070  95.070  1.00  0.00            C
ATOM     50  HB1 CYSPA   4      65.470  48.560  94.590  1.00  0.00            H
ATOM     51  HB2 CYSPA   4      66.890  48.860  95.610  1.00  0.00            H
ATOM     52  SG  CYSPA   4      67.390  47.470  93.750  1.00  0.00            S
ATOM     53  C1  CYSPA   4      67.750  49.030  92.840  1.00  0.00            C
ATOM     54  O1  CYSPA   4      67.040  49.980  92.850  1.00  0.00            O
ATOM     55  C2  CYSPA   4      69.110  49.070  92.120  1.00  0.00            C
```

ATOM	56	H2A	CYSPA	4	69.280	48.010	91.840	1.00	0.00	H
ATOM	57	H2B	CYSPA	4	69.770	49.280	92.990	1.00	0.00	H
ATOM	58	C3	CYSPA	4	69.170	50.050	90.920	1.00	0.00	C
ATOM	59	H3A	CYSPA	4	70.250	50.190	90.660	1.00	0.00	H
ATOM	60	H3B	CYSPA	4	68.930	51.100	91.220	1.00	0.00	H
ATOM	61	C4	CYSPA	4	68.280	49.560	89.780	1.00	0.00	C
ATOM	62	H4A	CYSPA	4	67.240	49.660	90.170	1.00	0.00	H
ATOM	63	H4B	CYSPA	4	68.510	48.480	89.700	1.00	0.00	H
ATOM	64	C5	CYSPA	4	68.330	50.330	88.480	1.00	0.00	C
ATOM	65	H5A	CYSPA	4	69.340	50.790	88.420	1.00	0.00	H
ATOM	66	H5B	CYSPA	4	67.730	51.260	88.610	1.00	0.00	H
ATOM	67	C6	CYSPA	4	67.880	49.610	87.240	1.00	0.00	C
ATOM	68	H6A	CYSPA	4	66.820	49.300	87.310	1.00	0.00	H
ATOM	69	H6B	CYSPA	4	68.540	48.730	87.150	1.00	0.00	H
ATOM	70	C7	CYSPA	4	68.100	50.530	85.990	1.00	0.00	C
ATOM	71	H7A	CYSPA	4	69.100	50.990	86.140	1.00	0.00	H
ATOM	72	H7B	CYSPA	4	67.390	51.380	85.840	1.00	0.00	H
ATOM	73	C8	CYSPA	4	68.000	49.670	84.730	1.00	0.00	C
ATOM	74	H8A	CYSPA	4	66.920	49.420	84.630	1.00	0.00	H
ATOM	75	H8B	CYSPA	4	68.520	48.690	84.760	1.00	0.00	H
ATOM	76	C9	CYSPA	4	68.370	50.380	83.420	1.00	0.00	C
ATOM	77	H9A	CYSPA	4	69.430	50.720	83.340	1.00	0.00	H
ATOM	78	H9B	CYSPA	4	67.670	51.250	83.360	1.00	0.00	H
ATOM	79	C10	CYSPA	4	68.190	49.470	82.200	1.00	0.00	C
ATOM	80	H10A	CYSPA	4	67.250	48.920	82.380	1.00	0.00	H
ATOM	81	H10B	CYSPA	4	68.980	48.700	82.190	1.00	0.00	H
ATOM	82	C11	CYSPA	4	68.160	50.310	80.900	1.00	0.00	C
ATOM	83	H11A	CYSPA	4	67.360	51.070	80.810	1.00	0.00	H
ATOM	84	H11B	CYSPA	4	67.970	49.590	80.070	1.00	0.00	H
ATOM	85	C12	CYSPA	4	69.540	50.980	80.580	1.00	0.00	C
ATOM	86	H12A	CYSPA	4	69.830	51.720	81.350	1.00	0.00	H
ATOM	87	H12B	CYSPA	4	69.380	51.500	79.610	1.00	0.00	H
ATOM	88	C13	CYSPA	4	70.630	49.970	80.430	1.00	0.00	C
ATOM	89	H13A	CYSPA	4	70.690	49.380	81.370	1.00	0.00	H
ATOM	90	H13B	CYSPA	4	71.540	50.610	80.430	1.00	0.00	H
ATOM	91	C14	CYSPA	4	70.510	49.070	79.240	1.00	0.00	C
ATOM	92	H14A	CYSPA	4	70.200	49.730	78.390	1.00	0.00	H
ATOM	93	H14B	CYSPA	4	69.620	48.410	79.310	1.00	0.00	H
ATOM	94	C15	CYSPA	4	71.730	48.330	78.810	1.00	0.00	C
ATOM	95	H15A	CYSPA	4	71.450	47.730	77.930	1.00	0.00	H
ATOM	96	H15B	CYSPA	4	72.000	47.560	79.560	1.00	0.00	H
ATOM	97	C16	CYSPA	4	72.950	49.250	78.450	1.00	0.00	C
ATOM	98	H16A	CYSPA	4	73.340	49.770	79.350	1.00	0.00	H
ATOM	99	H16B	CYSPA	4	73.880	48.680	78.210	1.00	0.00	H
ATOM	100	H16C	CYSPA	4	72.590	49.910	77.630	1.00	0.00	H
ATOM	101	N	PHE A	5	66.050	48.260	98.210	1.00	0.00	N
ATOM	102	HN	PHE A	5	67.040	48.170	98.160	1.00	0.00	H
ATOM	103	CA	PHE A	5	65.700	49.170	99.310	1.00	0.00	C
ATOM	104	HA	PHE A	5	64.790	48.730	99.690	1.00	0.00	H
ATOM	105	CB	PHE A	5	66.690	48.930	100.430	1.00	0.00	C
ATOM	106	HB1	PHE A	5	67.630	49.440	100.130	1.00	0.00	H
ATOM	107	HB2	PHE A	5	66.820	47.840	100.610	1.00	0.00	H
ATOM	108	CG	PHE A	5	66.260	49.480	101.750	1.00	0.00	C
ATOM	109	CD1	PHE A	5	67.180	50.090	102.610	1.00	0.00	C
ATOM	110	HD1	PHE A	5	68.180	50.220	102.230	1.00	0.00	H
ATOM	111	CE1	PHE A	5	66.850	50.510	103.890	1.00	0.00	C
ATOM	112	HE1	PHE A	5	67.660	50.870	104.500	1.00	0.00	H
ATOM	113	CZ	PHE A	5	65.540	50.270	104.370	1.00	0.00	C
ATOM	114	HZ	PHE A	5	65.240	50.660	105.340	1.00	0.00	H
ATOM	115	CD2	PHE A	5	64.930	49.220	102.220	1.00	0.00	C
ATOM	116	HD2	PHE A	5	64.220	48.690	101.610	1.00	0.00	H
ATOM	117	CE2	PHE A	5	64.640	49.650	103.480	1.00	0.00	C
ATOM	118	HE2	PHE A	5	63.680	49.390	103.900	1.00	0.00	H

ATOM	119	C	PHE	A	5	65.620	50.620	98.820	1.00	0.00	C
ATOM	120	O	PHE	A	5	66.570	51.080	98.260	1.00	0.00	O
ATOM	121	N	PRO	A	6	64.490	51.250	98.970	1.00	0.00	N
ATOM	122	CD	PRO	A	6	63.170	50.600	99.250	1.00	0.00	C
ATOM	123	HD1	PRO	A	6	63.080	50.120	100.240	1.00	0.00	H
ATOM	124	HD2	PRO	A	6	62.980	49.840	98.460	1.00	0.00	H
ATOM	125	CA	PRO	A	6	64.340	52.690	98.870	1.00	0.00	C
ATOM	126	HA	PRO	A	6	64.320	52.830	97.800	1.00	0.00	H
ATOM	127	CB	PRO	A	6	63.010	52.970	99.450	1.00	0.00	C
ATOM	128	HB1	PRO	A	6	62.440	53.730	98.880	1.00	0.00	H
ATOM	129	HB2	PRO	A	6	63.050	53.230	100.530	1.00	0.00	H
ATOM	130	CG	PRO	A	6	62.180	51.700	99.130	1.00	0.00	C
ATOM	131	HG1	PRO	A	6	61.190	51.560	99.620	1.00	0.00	H
ATOM	132	HG2	PRO	A	6	62.110	51.820	98.030	1.00	0.00	H
ATOM	133	C	PRO	A	6	65.420	53.670	99.390	1.00	0.00	C
ATOM	134	O	PRO	A	6	65.890	53.600	100.510	1.00	0.00	O
ATOM	135	N	SER	A	7	65.730	54.700	98.630	1.00	0.00	N
ATOM	136	HN	SER	A	7	65.330	54.780	97.720	1.00	0.00	H
ATOM	137	CA	SER	A	7	66.550	55.820	99.030	1.00	0.00	C
ATOM	138	HA	SER	A	7	67.470	55.410	99.430	1.00	0.00	H
ATOM	139	CB	SER	A	7	66.630	56.860	97.900	1.00	0.00	C
ATOM	140	HB1	SER	A	7	67.280	56.470	97.080	1.00	0.00	H
ATOM	141	HB2	SER	A	7	67.030	57.830	98.280	1.00	0.00	H
ATOM	142	OG	SER	A	7	65.310	57.140	97.380	1.00	0.00	O
ATOM	143	HG1	SER	A	7	65.060	56.390	96.840	1.00	0.00	H
ATOM	144	C	SER	A	7	65.920	56.570	100.240	1.00	0.00	C
ATOM	145	O	SER	A	7	66.600	56.850	101.200	1.00	0.00	O
ATOM	146	N	SER	A	8	64.570	56.840	100.190	1.00	0.00	N
ATOM	147	HN	SER	A	8	64.100	56.740	99.310	1.00	0.00	H
ATOM	148	CA	SER	A	8	63.910	57.410	101.350	1.00	0.00	C
ATOM	149	HA	SER	A	8	64.200	58.440	101.490	1.00	0.00	H
ATOM	150	CB	SER	A	8	62.400	57.400	101.020	1.00	0.00	C
ATOM	151	HB1	SER	A	8	61.990	56.360	100.950	1.00	0.00	H
ATOM	152	HB2	SER	A	8	62.230	57.980	100.090	1.00	0.00	H
ATOM	153	OG	SER	A	8	61.770	58.030	102.050	1.00	0.00	O
ATOM	154	HG1	SER	A	8	62.050	58.950	102.020	1.00	0.00	H
ATOM	155	C	SER	A	8	64.180	56.550	102.550	1.00	0.00	C
ATOM	156	O	SER	A	8	64.520	57.150	103.570	1.00	0.00	O
ATOM	157	N	LEU	A	9	63.970	55.230	102.490	1.00	0.00	N
ATOM	158	HN	LEU	A	9	63.760	54.900	101.570	1.00	0.00	H
ATOM	159	CA	LEU	A	9	64.170	54.360	103.650	1.00	0.00	C
ATOM	160	HA	LEU	A	9	63.520	54.870	104.350	1.00	0.00	H
ATOM	161	CB	LEU	A	9	63.480	53.010	103.270	1.00	0.00	C
ATOM	162	HB1	LEU	A	9	63.660	52.190	104.000	1.00	0.00	H
ATOM	163	HB2	LEU	A	9	63.860	52.620	102.300	1.00	0.00	H
ATOM	164	CG	LEU	A	9	61.890	53.050	103.160	1.00	0.00	C
ATOM	165	HG	LEU	A	9	61.630	53.770	102.350	1.00	0.00	H
ATOM	166	CD1	LEU	A	9	61.220	51.790	102.730	1.00	0.00	C
ATOM	167	HD11	LEU	A	9	61.170	50.950	103.450	1.00	0.00	H
ATOM	168	HD12	LEU	A	9	61.620	51.580	101.720	1.00	0.00	H
ATOM	169	HD13	LEU	A	9	60.150	52.020	102.540	1.00	0.00	H
ATOM	170	CD2	LEU	A	9	61.260	53.680	104.390	1.00	0.00	C
ATOM	171	HD21	LEU	A	9	60.160	53.510	104.320	1.00	0.00	H
ATOM	172	HD22	LEU	A	9	61.500	54.760	104.480	1.00	0.00	H
ATOM	173	HD23	LEU	A	9	61.610	53.220	105.340	1.00	0.00	H
ATOM	174	C	LEU	A	9	65.640	54.230	104.150	1.00	0.00	C
ATOM	175	O	LEU	A	9	65.870	54.050	105.360	1.00	0.00	O
ATOM	176	N	LYS	A	10	66.640	54.430	103.290	1.00	0.00	N
ATOM	177	HN	LYS	A	10	66.270	54.590	102.380	1.00	0.00	H
ATOM	178	CA	LYS	A	10	68.030	54.480	103.650	1.00	0.00	C
ATOM	179	HA	LYS	A	10	68.160	53.610	104.280	1.00	0.00	H
ATOM	180	CB	LYS	A	10	69.040	54.410	102.470	1.00	0.00	C
ATOM	181	HB1	LYS	A	10	70.030	54.860	102.670	1.00	0.00	H

ATOM	182	HB2	LYS	A	10	68.670	55.010	101.600	1.00	0.00	H
ATOM	183	CG	LYS	A	10	69.110	53.010	101.810	1.00	0.00	C
ATOM	184	HG1	LYS	A	10	68.070	52.800	101.470	1.00	0.00	H
ATOM	185	HG2	LYS	A	10	69.380	52.370	102.670	1.00	0.00	H
ATOM	186	CD	LYS	A	10	69.990	52.930	100.580	1.00	0.00	C
ATOM	187	HD1	LYS	A	10	69.580	53.760	99.960	1.00	0.00	H
ATOM	188	HD2	LYS	A	10	69.860	52.050	99.900	1.00	0.00	H
ATOM	189	CE	LYS	A	10	71.490	53.220	100.680	1.00	0.00	C
ATOM	190	HE1	LYS	A	10	71.820	53.950	101.450	1.00	0.00	H
ATOM	191	HE2	LYS	A	10	71.910	53.500	99.690	1.00	0.00	H
ATOM	192	NZ	LYS	A	10	72.200	51.940	101.080	1.00	0.00	N
ATOM	193	HZ1	LYS	A	10	73.200	52.150	101.290	1.00	0.00	H
ATOM	194	HZ2	LYS	A	10	72.130	51.340	100.230	1.00	0.00	H
ATOM	195	HZ3	LYS	A	10	71.720	51.460	101.870	1.00	0.00	H
ATOM	196	C	LYS	A	10	68.330	55.690	104.610	1.00	0.00	C
ATOM	197	O	LYS	A	10	68.980	55.590	105.660	1.00	0.00	O
ATOM	198	N	ARG	A	11	67.840	56.860	104.160	1.00	0.00	N
ATOM	199	HN	ARG	A	11	67.250	56.800	103.360	1.00	0.00	H
ATOM	200	CA	ARG	A	11	67.970	58.140	104.890	1.00	0.00	C
ATOM	201	HA	ARG	A	11	69.030	58.180	105.110	1.00	0.00	H
ATOM	202	CB	ARG	A	11	67.380	59.310	103.990	1.00	0.00	C
ATOM	203	HB1	ARG	A	11	67.510	60.300	104.490	1.00	0.00	H
ATOM	204	HB2	ARG	A	11	66.290	59.170	103.890	1.00	0.00	H
ATOM	205	CG	ARG	A	11	68.030	59.440	102.570	1.00	0.00	C
ATOM	206	HG1	ARG	A	11	68.120	58.420	102.140	1.00	0.00	H
ATOM	207	HG2	ARG	A	11	69.040	59.900	102.650	1.00	0.00	H
ATOM	208	CD	ARG	A	11	67.250	60.340	101.570	1.00	0.00	C
ATOM	209	HD1	ARG	A	11	67.100	61.300	102.110	1.00	0.00	H
ATOM	210	HD2	ARG	A	11	66.230	59.900	101.480	1.00	0.00	H
ATOM	211	NE	ARG	A	11	68.050	60.460	100.340	1.00	0.00	N
ATOM	212	HE	ARG	A	11	68.630	59.710	100.020	1.00	0.00	H
ATOM	213	CZ	ARG	A	11	67.950	61.410	99.460	1.00	0.00	C
ATOM	214	NH1	ARG	A	11	67.040	62.360	99.490	1.00	0.00	N
ATOM	215	HH11	ARG	A	11	66.930	62.970	98.700	1.00	0.00	H
ATOM	216	HH12	ARG	A	11	66.210	62.190	100.030	1.00	0.00	H
ATOM	217	NH2	ARG	A	11	68.620	61.330	98.340	1.00	0.00	N
ATOM	218	HH21	ARG	A	11	69.050	60.450	98.150	1.00	0.00	H
ATOM	219	HH22	ARG	A	11	68.580	62.080	97.680	1.00	0.00	H
ATOM	220	C	ARG	A	11	67.240	58.050	106.270	1.00	0.00	C
ATOM	221	O	ARG	A	11	67.710	58.490	107.250	1.00	0.00	O
ATOM	222	N	LEU	A	12	66.100	57.430	106.170	1.00	0.00	N
ATOM	223	HN	LEU	A	12	65.880	56.880	105.370	1.00	0.00	H
ATOM	224	CA	LEU	A	12	65.260	57.100	107.310	1.00	0.00	C
ATOM	225	HA	LEU	A	12	65.210	57.980	107.940	1.00	0.00	H
ATOM	226	CB	LEU	A	12	63.750	56.800	106.990	1.00	0.00	C
ATOM	227	HB1	LEU	A	12	63.350	56.360	107.920	1.00	0.00	H
ATOM	228	HB2	LEU	A	12	63.750	56.030	106.180	1.00	0.00	H
ATOM	229	CG	LEU	A	12	62.840	58.000	106.690	1.00	0.00	C
ATOM	230	HG	LEU	A	12	63.330	58.630	105.910	1.00	0.00	H
ATOM	231	CD1	LEU	A	12	61.490	57.640	105.970	1.00	0.00	C
ATOM	232	HD11	LEU	A	12	60.890	57.000	106.640	1.00	0.00	H
ATOM	233	HD12	LEU	A	12	61.600	57.020	105.050	1.00	0.00	H
ATOM	234	HD13	LEU	A	12	60.940	58.590	105.820	1.00	0.00	H
ATOM	235	CD2	LEU	A	12	62.590	59.010	107.830	1.00	0.00	C
ATOM	236	HD21	LEU	A	12	63.550	59.400	108.230	1.00	0.00	H
ATOM	237	HD22	LEU	A	12	61.950	58.520	108.590	1.00	0.00	H
ATOM	238	HD23	LEU	A	12	62.060	59.870	107.350	1.00	0.00	H
ATOM	239	C	LEU	A	12	65.770	56.010	108.240	1.00	0.00	C
ATOM	240	O	LEU	A	12	65.600	56.030	109.470	1.00	0.00	O
ATOM	241	N	LEU	A	13	66.570	55.050	107.740	1.00	0.00	N
ATOM	242	HN	LEU	A	13	66.680	54.990	106.760	1.00	0.00	H
ATOM	243	CA	LEU	A	13	67.370	54.130	108.540	1.00	0.00	C
ATOM	244	HA	LEU	A	13	66.740	53.540	109.190	1.00	0.00	H

ATOM	245	CB	LEU	A	13	68.140	53.200	107.710	1.00	0.00	C
ATOM	246	HB1	LEU	A	13	68.600	53.820	106.910	1.00	0.00	H
ATOM	247	HB2	LEU	A	13	67.650	52.340	107.200	1.00	0.00	H
ATOM	248	CG	LEU	A	13	69.420	52.460	108.370	1.00	0.00	C
ATOM	249	HG	LEU	A	13	70.150	53.170	108.820	1.00	0.00	H
ATOM	250	CD1	LEU	A	13	68.970	51.390	109.380	1.00	0.00	C
ATOM	251	HD11	LEU	A	13	68.510	51.950	110.220	1.00	0.00	H
ATOM	252	HD12	LEU	A	13	69.810	50.800	109.790	1.00	0.00	H
ATOM	253	HD13	LEU	A	13	68.290	50.610	108.970	1.00	0.00	H
ATOM	254	CD2	LEU	A	13	70.260	51.660	107.330	1.00	0.00	C
ATOM	255	HD21	LEU	A	13	70.480	52.380	106.510	1.00	0.00	H
ATOM	256	HD22	LEU	A	13	69.740	50.750	106.940	1.00	0.00	H
ATOM	257	HD23	LEU	A	13	71.180	51.210	107.760	1.00	0.00	H
ATOM	258	C	LEU	A	13	68.380	54.870	109.410	1.00	0.00	C
ATOM	259	O	LEU	A	13	68.440	54.580	110.570	1.00	0.00	O
ATOM	260	N	ILE	A	14	69.180	55.760	108.810	1.00	0.00	N
ATOM	261	HN	ILE	A	14	69.240	55.770	107.810	1.00	0.00	H
ATOM	262	CA	ILE	A	14	70.040	56.680	109.460	1.00	0.00	C
ATOM	263	HA	ILE	A	14	70.760	56.030	109.940	1.00	0.00	H
ATOM	264	CB	ILE	A	14	70.830	57.480	108.420	1.00	0.00	C
ATOM	265	HB	ILE	A	14	70.150	57.950	107.690	1.00	0.00	H
ATOM	266	CG2	ILE	A	14	71.590	58.720	109.040	1.00	0.00	C
ATOM	267	HG21	ILE	A	14	72.170	59.180	108.210	1.00	0.00	H
ATOM	268	HG22	ILE	A	14	72.250	58.410	109.880	1.00	0.00	H
ATOM	269	HG23	ILE	A	14	70.900	59.510	109.380	1.00	0.00	H
ATOM	270	CG1	ILE	A	14	71.750	56.460	107.650	1.00	0.00	C
ATOM	271	HG11	ILE	A	14	71.180	55.690	107.090	1.00	0.00	H
ATOM	272	HG12	ILE	A	14	72.420	55.840	108.290	1.00	0.00	H
ATOM	273	CD	ILE	A	14	72.780	57.110	106.670	1.00	0.00	C
ATOM	274	HD1	ILE	A	14	72.240	57.700	105.900	1.00	0.00	H
ATOM	275	HD2	ILE	A	14	73.350	56.370	106.070	1.00	0.00	H
ATOM	276	HD3	ILE	A	14	73.480	57.820	107.140	1.00	0.00	H
ATOM	277	C	ILE	A	14	69.430	57.530	110.610	1.00	0.00	C
ATOM	278	O	ILE	A	14	69.970	57.830	111.700	1.00	0.00	O
ATOM	279	N	ILE	A	15	68.200	58.080	110.380	1.00	0.00	N
ATOM	280	HN	ILE	A	15	67.650	57.970	109.550	1.00	0.00	H
ATOM	281	CA	ILE	A	15	67.470	58.810	111.370	1.00	0.00	C
ATOM	282	HA	ILE	A	15	68.070	59.570	111.840	1.00	0.00	H
ATOM	283	CB	ILE	A	15	66.280	59.430	110.630	1.00	0.00	C
ATOM	284	HB	ILE	A	15	65.800	58.850	109.810	1.00	0.00	H
ATOM	285	CG2	ILE	A	15	65.000	59.660	111.530	1.00	0.00	C
ATOM	286	HG21	ILE	A	15	64.110	60.080	111.030	1.00	0.00	H
ATOM	287	HG22	ILE	A	15	65.240	60.330	112.380	1.00	0.00	H
ATOM	288	HG23	ILE	A	15	64.580	58.700	111.910	1.00	0.00	H
ATOM	289	CG1	ILE	A	15	66.620	60.820	110.020	1.00	0.00	C
ATOM	290	HG11	ILE	A	15	67.620	60.840	109.540	1.00	0.00	H
ATOM	291	HG12	ILE	A	15	66.580	61.690	110.710	1.00	0.00	H
ATOM	292	CD	ILE	A	15	65.770	61.270	108.850	1.00	0.00	C
ATOM	293	HD1	ILE	A	15	66.320	62.140	108.430	1.00	0.00	H
ATOM	294	HD2	ILE	A	15	64.770	61.470	109.280	1.00	0.00	H
ATOM	295	HD3	ILE	A	15	65.750	60.380	108.180	1.00	0.00	H
ATOM	296	C	ILE	A	15	67.000	57.950	112.520	1.00	0.00	C
ATOM	297	O	ILE	A	15	67.140	58.240	113.760	1.00	0.00	O
ATOM	298	N	VAL	A	16	66.540	56.660	112.270	1.00	0.00	N
ATOM	299	HN	VAL	A	16	66.320	56.440	111.320	1.00	0.00	H
ATOM	300	CA	VAL	A	16	66.340	55.640	113.290	1.00	0.00	C
ATOM	301	HA	VAL	A	16	65.800	56.160	114.060	1.00	0.00	H
ATOM	302	CB	VAL	A	16	65.430	54.630	112.650	1.00	0.00	C
ATOM	303	HB	VAL	A	16	65.840	54.250	111.690	1.00	0.00	H
ATOM	304	CG1	VAL	A	16	65.180	53.410	113.520	1.00	0.00	C
ATOM	305	HG11	VAL	A	16	64.590	53.660	114.430	1.00	0.00	H
ATOM	306	HG12	VAL	A	16	66.180	52.970	113.740	1.00	0.00	H
ATOM	307	HG13	VAL	A	16	64.610	52.620	112.980	1.00	0.00	H

ATOM	308	CG2	VAL	A	16	64.050	55.280	112.380	1.00	0.00	C
ATOM	309	HG21	VAL	A	16	64.090	55.780	111.390	1.00	0.00	H
ATOM	310	HG22	VAL	A	16	63.760	55.980	113.190	1.00	0.00	H
ATOM	311	HG23	VAL	A	16	63.310	54.450	112.380	1.00	0.00	H
ATOM	312	C	VAL	A	16	67.610	55.160	114.100	1.00	0.00	C
ATOM	313	O	VAL	A	16	67.530	54.890	115.290	1.00	0.00	O
ATOM	314	N	VAL	A	17	68.770	55.130	113.390	1.00	0.00	N
ATOM	315	HN	VAL	A	17	68.760	55.030	112.400	1.00	0.00	H
ATOM	316	CA	VAL	A	17	70.060	54.940	114.050	1.00	0.00	C
ATOM	317	HA	VAL	A	17	69.890	54.040	114.610	1.00	0.00	H
ATOM	318	CB	VAL	A	17	71.160	54.710	112.970	1.00	0.00	C
ATOM	319	HB	VAL	A	17	71.010	55.370	112.090	1.00	0.00	H
ATOM	320	CG1	VAL	A	17	72.560	55.010	113.610	1.00	0.00	C
ATOM	321	HG11	VAL	A	17	73.330	54.730	112.860	1.00	0.00	H
ATOM	322	HG12	VAL	A	17	72.710	54.330	114.470	1.00	0.00	H
ATOM	323	HG13	VAL	A	17	72.720	56.080	113.880	1.00	0.00	H
ATOM	324	CG2	VAL	A	17	71.180	53.180	112.630	1.00	0.00	C
ATOM	325	HG21	VAL	A	17	71.980	52.970	111.890	1.00	0.00	H
ATOM	326	HG22	VAL	A	17	70.240	52.770	112.200	1.00	0.00	H
ATOM	327	HG23	VAL	A	17	71.400	52.580	113.540	1.00	0.00	H
ATOM	328	C	VAL	A	17	70.460	55.950	115.110	1.00	0.00	C
ATOM	329	O	VAL	A	17	70.680	55.500	116.240	1.00	0.00	O
ATOM	330	N	VAL	A	18	70.260	57.250	114.790	1.00	0.00	N
ATOM	331	HN	VAL	A	18	69.930	57.430	113.870	1.00	0.00	H
ATOM	332	CA	VAL	A	18	70.280	58.340	115.750	1.00	0.00	C
ATOM	333	HA	VAL	A	18	71.200	58.490	116.280	1.00	0.00	H
ATOM	334	CB	VAL	A	18	70.030	59.690	115.010	1.00	0.00	C
ATOM	335	HB	VAL	A	18	69.230	59.610	114.240	1.00	0.00	H
ATOM	336	CG1	VAL	A	18	69.880	60.840	116.070	1.00	0.00	C
ATOM	337	HG11	VAL	A	18	70.680	60.730	116.840	1.00	0.00	H
ATOM	338	HG12	VAL	A	18	68.910	60.730	116.600	1.00	0.00	H
ATOM	339	HG13	VAL	A	18	70.000	61.880	115.700	1.00	0.00	H
ATOM	340	CG2	VAL	A	18	71.310	60.020	114.200	1.00	0.00	C
ATOM	341	HG21	VAL	A	18	72.200	60.180	114.850	1.00	0.00	H
ATOM	342	HG22	VAL	A	18	70.990	60.950	113.680	1.00	0.00	H
ATOM	343	HG23	VAL	A	18	71.530	59.300	113.380	1.00	0.00	H
ATOM	344	C	VAL	A	18	69.300	58.280	116.930	1.00	0.00	C
ATOM	345	O	VAL	A	18	69.720	58.490	118.110	1.00	0.00	O
ATOM	346	N	ILE	A	19	68.000	57.930	116.650	1.00	0.00	N
ATOM	347	HN	ILE	A	19	67.650	57.880	115.720	1.00	0.00	H
ATOM	348	CA	ILE	A	19	66.990	57.730	117.730	1.00	0.00	C
ATOM	349	HA	ILE	A	19	67.050	58.620	118.340	1.00	0.00	H
ATOM	350	CB	ILE	A	19	65.520	57.720	117.160	1.00	0.00	C
ATOM	351	HB	ILE	A	19	65.400	56.900	116.420	1.00	0.00	H
ATOM	352	CG2	ILE	A	19	64.550	57.310	118.360	1.00	0.00	C
ATOM	353	HG21	ILE	A	19	64.850	57.830	119.300	1.00	0.00	H
ATOM	354	HG22	ILE	A	19	64.430	56.200	118.420	1.00	0.00	H
ATOM	355	HG23	ILE	A	19	63.540	57.730	118.180	1.00	0.00	H
ATOM	356	CG1	ILE	A	19	65.150	59.080	116.540	1.00	0.00	C
ATOM	357	HG11	ILE	A	19	66.010	59.570	116.050	1.00	0.00	H
ATOM	358	HG12	ILE	A	19	64.870	59.790	117.350	1.00	0.00	H
ATOM	359	CD	ILE	A	19	63.960	59.060	115.600	1.00	0.00	C
ATOM	360	HD1	ILE	A	19	63.660	60.060	115.220	1.00	0.00	H
ATOM	361	HD2	ILE	A	19	63.070	58.620	116.120	1.00	0.00	H
ATOM	362	HD3	ILE	A	19	64.080	58.370	114.740	1.00	0.00	H
ATOM	363	C	ILE	A	19	67.280	56.570	118.600	1.00	0.00	C
ATOM	364	O	ILE	A	19	67.270	56.600	119.840	1.00	0.00	O
ATOM	365	N	GLU	A	20	67.720	55.470	118.030	1.00	0.00	N
ATOM	366	HN	GLU	A	20	67.980	55.410	117.070	1.00	0.00	H
ATOM	367	CA	GLU	A	20	68.150	54.230	118.810	1.00	0.00	C
ATOM	368	HA	GLU	A	20	67.290	54.040	119.430	1.00	0.00	H
ATOM	369	CB	GLU	A	20	68.350	53.000	117.870	1.00	0.00	C
ATOM	370	HB1	GLU	A	20	69.120	53.180	117.090	1.00	0.00	H

ATOM	371	HB2	GLU	A	20	67.370	52.940	117.350	1.00	0.00	H
ATOM	372	CG	GLU	A	20	68.570	51.530	118.460	1.00	0.00	C
ATOM	373	HG1	GLU	A	20	68.350	50.720	117.740	1.00	0.00	H
ATOM	374	HG2	GLU	A	20	67.810	51.370	119.260	1.00	0.00	H
ATOM	375	CD	GLU	A	20	70.010	51.280	118.860	1.00	0.00	C
ATOM	376	OE1	GLU	A	20	70.960	51.690	118.150	1.00	0.00	O
ATOM	377	OE2	GLU	A	20	70.230	50.690	119.920	1.00	0.00	O
ATOM	378	C	GLU	A	20	69.340	54.390	119.760	1.00	0.00	C
ATOM	379	O	GLU	A	20	69.430	53.850	120.870	1.00	0.00	O
ATOM	380	N	LEU	A	21	70.240	55.250	119.360	1.00	0.00	N
ATOM	381	HN	LEU	A	21	70.270	55.490	118.390	1.00	0.00	H
ATOM	382	CA	LEU	A	21	71.400	55.730	120.130	1.00	0.00	C
ATOM	383	HA	LEU	A	21	71.870	54.820	120.460	1.00	0.00	H
ATOM	384	CB	LEU	A	21	72.420	56.430	119.230	1.00	0.00	C
ATOM	385	HB1	LEU	A	21	71.970	57.290	118.700	1.00	0.00	H
ATOM	386	HB2	LEU	A	21	72.860	55.770	118.440	1.00	0.00	H
ATOM	387	CG	LEU	A	21	73.670	57.070	120.000	1.00	0.00	C
ATOM	388	HG	LEU	A	21	73.220	57.800	120.710	1.00	0.00	H
ATOM	389	CD1	LEU	A	21	74.400	55.950	120.880	1.00	0.00	C
ATOM	390	HD11	LEU	A	21	74.890	55.370	120.070	1.00	0.00	H
ATOM	391	HD12	LEU	A	21	73.680	55.440	121.560	1.00	0.00	H
ATOM	392	HD13	LEU	A	21	75.160	56.430	121.520	1.00	0.00	H
ATOM	393	CD2	LEU	A	21	74.580	57.720	119.040	1.00	0.00	C
ATOM	394	HD21	LEU	A	21	74.050	58.480	118.410	1.00	0.00	H
ATOM	395	HD22	LEU	A	21	74.930	56.990	118.270	1.00	0.00	H
ATOM	396	HD23	LEU	A	21	75.460	58.130	119.580	1.00	0.00	H
ATOM	397	C	LEU	A	21	70.940	56.460	121.320	1.00	0.00	C
ATOM	398	O	LEU	A	21	71.430	56.190	122.420	1.00	0.00	O
ATOM	399	N	VAL	A	22	69.990	57.380	121.190	1.00	0.00	N
ATOM	400	HN	VAL	A	22	69.930	57.780	120.280	1.00	0.00	H
ATOM	401	CA	VAL	A	22	69.300	58.030	122.340	1.00	0.00	C
ATOM	402	HA	VAL	A	22	70.140	58.310	122.970	1.00	0.00	H
ATOM	403	CB	VAL	A	22	68.450	59.310	121.980	1.00	0.00	C
ATOM	404	HB	VAL	A	22	67.890	59.050	121.060	1.00	0.00	H
ATOM	405	CG1	VAL	A	22	67.660	59.790	123.230	1.00	0.00	C
ATOM	406	HG11	VAL	A	22	68.320	59.730	124.130	1.00	0.00	H
ATOM	407	HG12	VAL	A	22	66.720	59.240	123.430	1.00	0.00	H
ATOM	408	HG13	VAL	A	22	67.500	60.870	123.040	1.00	0.00	H
ATOM	409	CG2	VAL	A	22	69.510	60.310	121.460	1.00	0.00	C
ATOM	410	HG21	VAL	A	22	69.810	59.930	120.460	1.00	0.00	H
ATOM	411	HG22	VAL	A	22	70.360	60.500	122.150	1.00	0.00	H
ATOM	412	HG23	VAL	A	22	69.020	61.280	121.260	1.00	0.00	H
ATOM	413	C	VAL	A	22	68.600	57.000	123.270	1.00	0.00	C
ATOM	414	O	VAL	A	22	68.600	57.100	124.480	1.00	0.00	O
ATOM	415	N	VAL	A	23	67.930	56.030	122.700	1.00	0.00	N
ATOM	416	HN	VAL	A	23	67.920	55.940	121.700	1.00	0.00	H
ATOM	417	CA	VAL	A	23	67.350	54.980	123.470	1.00	0.00	C
ATOM	418	HA	VAL	A	23	66.760	55.510	124.210	1.00	0.00	H
ATOM	419	CB	VAL	A	23	66.480	54.060	122.570	1.00	0.00	C
ATOM	420	HB	VAL	A	23	66.940	53.640	121.650	1.00	0.00	H
ATOM	421	CG1	VAL	A	23	65.900	52.840	123.370	1.00	0.00	C
ATOM	422	HG11	VAL	A	23	66.610	52.060	123.720	1.00	0.00	H
ATOM	423	HG12	VAL	A	23	65.220	52.380	122.620	1.00	0.00	H
ATOM	424	HG13	VAL	A	23	65.330	53.210	124.250	1.00	0.00	H
ATOM	425	CG2	VAL	A	23	65.260	55.020	122.150	1.00	0.00	C
ATOM	426	HG21	VAL	A	23	64.610	55.390	122.970	1.00	0.00	H
ATOM	427	HG22	VAL	A	23	64.590	54.460	121.470	1.00	0.00	H
ATOM	428	HG23	VAL	A	23	65.650	55.930	121.640	1.00	0.00	H
ATOM	429	C	VAL	A	23	68.230	54.120	124.370	1.00	0.00	C
ATOM	430	O	VAL	A	23	67.860	53.610	125.380	1.00	0.00	O
ATOM	431	N	LYS	A	24	69.480	53.860	123.960	1.00	0.00	N
ATOM	432	HN	LYS	A	24	69.840	54.170	123.080	1.00	0.00	H
ATOM	433	CA	LYS	A	24	70.430	53.190	124.890	1.00	0.00	C

ATOM	434	HA	LYS	A	24	70.100	52.200	125.150	1.00	0.00	H
ATOM	435	CB	LYS	A	24	71.810	52.980	124.070	1.00	0.00	C
ATOM	436	HB1	LYS	A	24	72.540	52.630	124.830	1.00	0.00	H
ATOM	437	HB2	LYS	A	24	72.060	54.000	123.720	1.00	0.00	H
ATOM	438	CG	LYS	A	24	71.760	52.010	122.830	1.00	0.00	C
ATOM	439	HG1	LYS	A	24	71.280	52.490	121.950	1.00	0.00	H
ATOM	440	HG2	LYS	A	24	71.120	51.170	123.170	1.00	0.00	H
ATOM	441	CD	LYS	A	24	73.220	51.590	122.430	1.00	0.00	C
ATOM	442	HD1	LYS	A	24	73.580	51.080	123.350	1.00	0.00	H
ATOM	443	HD2	LYS	A	24	73.860	52.470	122.180	1.00	0.00	H
ATOM	444	CE	LYS	A	24	73.340	50.480	121.410	1.00	0.00	C
ATOM	445	HE1	LYS	A	24	72.760	49.560	121.630	1.00	0.00	H
ATOM	446	HE2	LYS	A	24	74.300	50.020	121.100	1.00	0.00	H
ATOM	447	NZ	LYS	A	24	72.870	51.070	120.100	1.00	0.00	N
ATOM	448	HZ1	LYS	A	24	72.980	50.420	119.300	1.00	0.00	H
ATOM	449	HZ2	LYS	A	24	73.210	52.010	119.820	1.00	0.00	H
ATOM	450	HZ3	LYS	A	24	71.830	51.110	120.060	1.00	0.00	H
ATOM	451	C	LYS	A	24	70.670	53.810	126.280	1.00	0.00	C
ATOM	452	O	LYS	A	24	70.590	53.280	127.390	1.00	0.00	O
ATOM	453	N	VAL	A	25	70.710	55.140	126.120	1.00	0.00	N
ATOM	454	HN	VAL	A	25	70.560	55.550	125.220	1.00	0.00	H
ATOM	455	CA	VAL	A	25	70.940	56.070	127.150	1.00	0.00	C
ATOM	456	HA	VAL	A	25	71.730	55.720	127.790	1.00	0.00	H
ATOM	457	CB	VAL	A	25	71.130	57.440	126.590	1.00	0.00	C
ATOM	458	HB	VAL	A	25	70.260	57.750	125.980	1.00	0.00	H
ATOM	459	CG1	VAL	A	25	71.190	58.590	127.690	1.00	0.00	C
ATOM	460	HG11	VAL	A	25	70.210	58.700	128.200	1.00	0.00	H
ATOM	461	HG12	VAL	A	25	71.350	59.510	127.080	1.00	0.00	H
ATOM	462	HG13	VAL	A	25	72.020	58.480	128.420	1.00	0.00	H
ATOM	463	CG2	VAL	A	25	72.460	57.400	125.890	1.00	0.00	C
ATOM	464	HG21	VAL	A	25	72.650	58.370	125.390	1.00	0.00	H
ATOM	465	HG22	VAL	A	25	72.440	56.720	125.000	1.00	0.00	H
ATOM	466	HG23	VAL	A	25	73.400	57.260	126.470	1.00	0.00	H
ATOM	467	C	VAL	A	25	69.690	56.000	128.060	1.00	0.00	C
ATOM	468	O	VAL	A	25	69.850	55.790	129.270	1.00	0.00	O
ATOM	469	N	ILE	A	26	68.510	56.230	127.440	1.00	0.00	N
ATOM	470	HN	ILE	A	26	68.530	56.650	126.540	1.00	0.00	H
ATOM	471	CA	ILE	A	26	67.200	56.160	128.190	1.00	0.00	C
ATOM	472	HA	ILE	A	26	67.410	56.810	129.030	1.00	0.00	H
ATOM	473	CB	ILE	A	26	65.980	56.470	127.250	1.00	0.00	C
ATOM	474	HB	ILE	A	26	66.130	55.790	126.390	1.00	0.00	H
ATOM	475	CG2	ILE	A	26	64.640	56.100	127.960	1.00	0.00	C
ATOM	476	HG21	ILE	A	26	64.440	56.620	128.920	1.00	0.00	H
ATOM	477	HG22	ILE	A	26	64.510	55.010	128.150	1.00	0.00	H
ATOM	478	HG23	ILE	A	26	63.780	56.370	127.320	1.00	0.00	H
ATOM	479	CG1	ILE	A	26	65.960	57.860	126.690	1.00	0.00	C
ATOM	480	HG11	ILE	A	26	65.060	58.020	126.060	1.00	0.00	H
ATOM	481	HG12	ILE	A	26	66.790	58.030	125.980	1.00	0.00	H
ATOM	482	CD	ILE	A	26	66.020	58.920	127.780	1.00	0.00	C
ATOM	483	HD1	ILE	A	26	65.070	58.840	128.350	1.00	0.00	H
ATOM	484	HD2	ILE	A	26	66.030	59.990	127.470	1.00	0.00	H
ATOM	485	HD3	ILE	A	26	66.880	58.850	128.480	1.00	0.00	H
ATOM	486	C	ILE	A	26	66.940	54.760	128.830	1.00	0.00	C
ATOM	487	O	ILE	A	26	66.450	54.790	129.980	1.00	0.00	O
ATOM	488	N	VAL	A	27	67.170	53.580	128.170	1.00	0.00	N
ATOM	489	HN	VAL	A	27	67.600	53.590	127.270	1.00	0.00	H
ATOM	490	CA	VAL	A	27	67.150	52.280	128.880	1.00	0.00	C
ATOM	491	HA	VAL	A	27	66.150	52.140	129.270	1.00	0.00	H
ATOM	492	CB	VAL	A	27	67.500	51.150	127.900	1.00	0.00	C
ATOM	493	HB	VAL	A	27	68.360	51.510	127.300	1.00	0.00	H
ATOM	494	CG1	VAL	A	27	67.820	49.840	128.660	1.00	0.00	C
ATOM	495	HG11	VAL	A	27	68.000	48.930	128.060	1.00	0.00	H
ATOM	496	HG12	VAL	A	27	67.020	49.550	129.380	1.00	0.00	H

ATOM	497	HG13	VAL	A	27	68.760	49.950	129.250	1.00	0.00	H
ATOM	498	CG2	VAL	A	27	66.300	50.840	126.990	1.00	0.00	C
ATOM	499	HG21	VAL	A	27	65.660	50.030	127.400	1.00	0.00	H
ATOM	500	HG22	VAL	A	27	66.690	50.480	126.010	1.00	0.00	H
ATOM	501	HG23	VAL	A	27	65.690	51.740	126.800	1.00	0.00	H
ATOM	502	C	VAL	A	27	68.170	52.310	130.120	1.00	0.00	C
ATOM	503	O	VAL	A	27	67.900	51.770	131.200	1.00	0.00	O
ATOM	504	N	GLY	A	28	69.350	52.880	129.960	1.00	0.00	N
ATOM	505	HN	GLY	A	28	69.710	53.220	129.100	1.00	0.00	H
ATOM	506	CA	GLY	A	28	70.290	52.940	131.060	1.00	0.00	C
ATOM	507	HA1	GLY	A	28	71.100	53.550	130.710	1.00	0.00	H
ATOM	508	HA2	GLY	A	28	70.650	51.950	131.310	1.00	0.00	H
ATOM	509	C	GLY	A	28	69.800	53.680	132.270	1.00	0.00	C
ATOM	510	O	GLY	A	28	69.970	53.370	133.400	1.00	0.00	O
ATOM	511	N	ALA	A	29	69.020	54.690	132.060	1.00	0.00	N
ATOM	512	HN	ALA	A	29	68.890	55.100	131.160	1.00	0.00	H
ATOM	513	CA	ALA	A	29	68.290	55.420	133.050	1.00	0.00	C
ATOM	514	HA	ALA	A	29	69.030	55.620	133.810	1.00	0.00	H
ATOM	515	CB	ALA	A	29	67.750	56.740	132.400	1.00	0.00	C
ATOM	516	HB1	ALA	A	29	67.200	56.500	131.460	1.00	0.00	H
ATOM	517	HB2	ALA	A	29	68.660	57.330	132.150	1.00	0.00	H
ATOM	518	HB3	ALA	A	29	67.160	57.430	133.040	1.00	0.00	H
ATOM	519	C	ALA	A	29	67.150	54.610	133.720	1.00	0.00	C
ATOM	520	O	ALA	A	29	66.890	54.580	134.930	1.00	0.00	O
ATOM	521	N	LEU	A	30	66.360	53.850	132.920	1.00	0.00	N
ATOM	522	HN	LEU	A	30	66.470	53.780	131.930	1.00	0.00	H
ATOM	523	CA	LEU	A	30	65.310	52.950	133.470	1.00	0.00	C
ATOM	524	HA	LEU	A	30	64.730	53.460	134.220	1.00	0.00	H
ATOM	525	CB	LEU	A	30	64.320	52.430	132.450	1.00	0.00	C
ATOM	526	HB1	LEU	A	30	64.920	52.310	131.520	1.00	0.00	H
ATOM	527	HB2	LEU	A	30	63.590	53.220	132.150	1.00	0.00	H
ATOM	528	CG	LEU	A	30	63.480	51.100	132.600	1.00	0.00	C
ATOM	529	HG	LEU	A	30	64.230	50.320	132.810	1.00	0.00	H
ATOM	530	CD1	LEU	A	30	62.590	51.100	133.790	1.00	0.00	C
ATOM	531	HD11	LEU	A	30	61.950	51.990	133.640	1.00	0.00	H
ATOM	532	HD12	LEU	A	30	63.070	51.120	134.800	1.00	0.00	H
ATOM	533	HD13	LEU	A	30	61.940	50.210	133.830	1.00	0.00	H
ATOM	534	CD2	LEU	A	30	62.800	50.650	131.300	1.00	0.00	C
ATOM	535	HD21	LEU	A	30	63.520	50.760	130.460	1.00	0.00	H
ATOM	536	HD22	LEU	A	30	61.860	51.230	131.240	1.00	0.00	H
ATOM	537	HD23	LEU	A	30	62.580	49.580	131.490	1.00	0.00	H
ATOM	538	C	LEU	A	30	65.970	51.860	134.290	1.00	0.00	C
ATOM	539	O	LEU	A	30	65.530	51.510	135.380	1.00	0.00	O
ATOM	540	N	LEU	A	31	67.040	51.270	133.780	1.00	0.00	N
ATOM	541	HN	LEU	A	31	67.340	51.500	132.860	1.00	0.00	H
ATOM	542	CA	LEU	A	31	67.790	50.290	134.460	1.00	0.00	C
ATOM	543	HA	LEU	A	31	67.140	49.540	134.880	1.00	0.00	H
ATOM	544	CB	LEU	A	31	68.870	49.610	133.640	1.00	0.00	C
ATOM	545	HB1	LEU	A	31	69.530	49.060	134.340	1.00	0.00	H
ATOM	546	HB2	LEU	A	31	69.510	50.340	133.110	1.00	0.00	H
ATOM	547	CG	LEU	A	31	68.450	48.470	132.680	1.00	0.00	C
ATOM	548	HG	LEU	A	31	67.720	48.970	132.000	1.00	0.00	H
ATOM	549	CD1	LEU	A	31	69.720	48.010	131.890	1.00	0.00	C
ATOM	550	HD11	LEU	A	31	70.440	47.610	132.640	1.00	0.00	H
ATOM	551	HD12	LEU	A	31	70.190	48.800	131.260	1.00	0.00	H
ATOM	552	HD13	LEU	A	31	69.470	47.160	131.220	1.00	0.00	H
ATOM	553	CD2	LEU	A	31	67.730	47.340	133.350	1.00	0.00	C
ATOM	554	HD21	LEU	A	31	66.740	47.700	133.700	1.00	0.00	H
ATOM	555	HD22	LEU	A	31	68.260	46.810	134.160	1.00	0.00	H
ATOM	556	HD23	LEU	A	31	67.560	46.610	132.530	1.00	0.00	H
ATOM	557	C	LEU	A	31	68.460	50.820	135.760	1.00	0.00	C
ATOM	558	O	LEU	A	31	68.430	50.130	136.760	1.00	0.00	O
ATOM	559	N	MET	A	32	69.070	52.020	135.710	1.00	0.00	N

ATOM	560	HN	MET	A	32	69.280	52.520	134.870	1.00	0.00	H
ATOM	561	CA	MET	A	32	69.450	52.680	136.950	1.00	0.00	C
ATOM	562	HA	MET	A	32	70.000	51.970	137.560	1.00	0.00	H
ATOM	563	CB	MET	A	32	70.350	53.900	136.900	1.00	0.00	C
ATOM	564	HB1	MET	A	32	70.280	54.430	137.880	1.00	0.00	H
ATOM	565	HB2	MET	A	32	69.880	54.570	136.150	1.00	0.00	H
ATOM	566	CG	MET	A	32	71.890	53.500	136.700	1.00	0.00	C
ATOM	567	HG1	MET	A	32	71.980	53.060	135.680	1.00	0.00	H
ATOM	568	HG2	MET	A	32	72.140	52.690	137.410	1.00	0.00	H
ATOM	569	SD	MET	A	32	72.930	54.930	136.820	1.00	0.00	S
ATOM	570	CE	MET	A	32	73.210	55.210	135.060	1.00	0.00	C
ATOM	571	HE1	MET	A	32	74.030	55.930	134.870	1.00	0.00	H
ATOM	572	HE2	MET	A	32	72.320	55.670	134.580	1.00	0.00	H
ATOM	573	HE3	MET	A	32	73.530	54.280	134.540	1.00	0.00	H
ATOM	574	C	MET	A	32	68.220	52.880	137.900	1.00	0.00	C
ATOM	575	O	MET	A	32	68.190	52.500	139.040	1.00	0.00	O
ATOM	576	N	GLY	A	33	67.130	53.520	137.390	1.00	0.00	N
ATOM	577	HN	GLY	A	33	67.070	53.810	136.440	1.00	0.00	H
ATOM	578	CA	GLY	A	33	65.920	53.810	138.170	1.00	0.00	C
ATOM	579	HA1	GLY	A	33	65.590	52.900	138.630	1.00	0.00	H
ATOM	580	HA2	GLY	A	33	65.260	54.140	137.380	1.00	0.00	H
ATOM	581	C	GLY	A	33	65.930	54.810	139.270	1.00	0.00	C
ATOM	582	O	GLY	A	33	66.980	55.320	139.590	1.00	0.00	O
ATOM	583	N	LEU	A	34	64.790	55.120	139.900	1.00	0.00	N
ATOM	584	HN	LEU	A	34	63.940	54.670	139.640	1.00	0.00	H
ATOM	585	CA	LEU	A	34	64.660	56.150	140.920	1.00	0.00	C
ATOM	586	HA	LEU	A	34	65.400	56.900	140.680	1.00	0.00	H
ATOM	587	CB	LEU	A	34	63.180	56.680	140.960	1.00	0.00	C
ATOM	588	HB1	LEU	A	34	62.950	57.220	141.900	1.00	0.00	H
ATOM	589	HB2	LEU	A	34	62.600	55.730	140.940	1.00	0.00	H
ATOM	590	CG	LEU	A	34	62.850	57.570	139.780	1.00	0.00	C
ATOM	591	HG	LEU	A	34	63.220	57.170	138.820	1.00	0.00	H
ATOM	592	CD1	LEU	A	34	61.300	57.630	139.640	1.00	0.00	C
ATOM	593	HD11	LEU	A	34	61.050	56.590	139.320	1.00	0.00	H
ATOM	594	HD12	LEU	A	34	61.000	58.380	138.890	1.00	0.00	H
ATOM	595	HD13	LEU	A	34	60.880	58.000	140.600	1.00	0.00	H
ATOM	596	CD2	LEU	A	34	63.290	59.000	139.900	1.00	0.00	C
ATOM	597	HD21	LEU	A	34	63.050	59.530	140.840	1.00	0.00	H
ATOM	598	HD22	LEU	A	34	63.020	59.590	139.000	1.00	0.00	H
ATOM	599	HD23	LEU	A	34	64.400	58.930	139.840	1.00	0.00	H
ATOM	600	C	LEU	A	34	65.160	55.620	0.160	1.00	0.00	C
ATOM	601	OT1	LEU	A	34	65.540	54.410	0.330	1.00	0.00	O
ATOM	602	OT2	LEU	A	34	65.080	56.460	1.090	1.00	0.00	O

Quality of AlphaFold Best Predicted Model

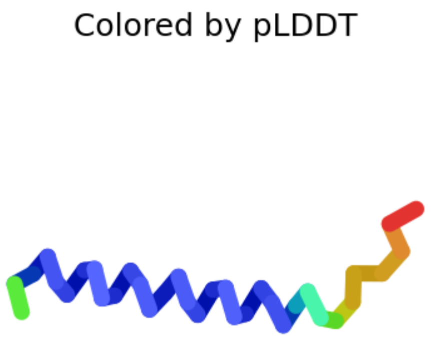

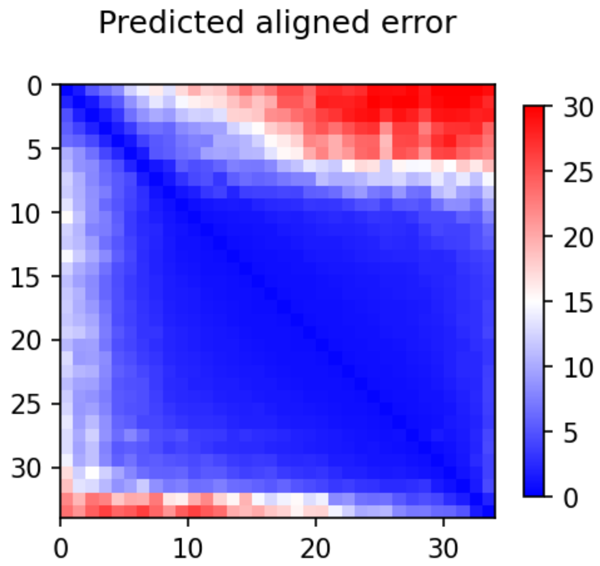

The predicted local distance difference test (pLDDT) score (0-100) is a per-residue confidence score, with values greater than 90 indicating high confidence, and values below 50 indicating low confidence.

High confidence scores Colored in Blue on structure.

Model structural quality of the AlphaFold predicted structure analyzed by PROCHECK (Laskowski et al., 1993, 1996) generated with PDBsum (https://ebi.ac.uk).

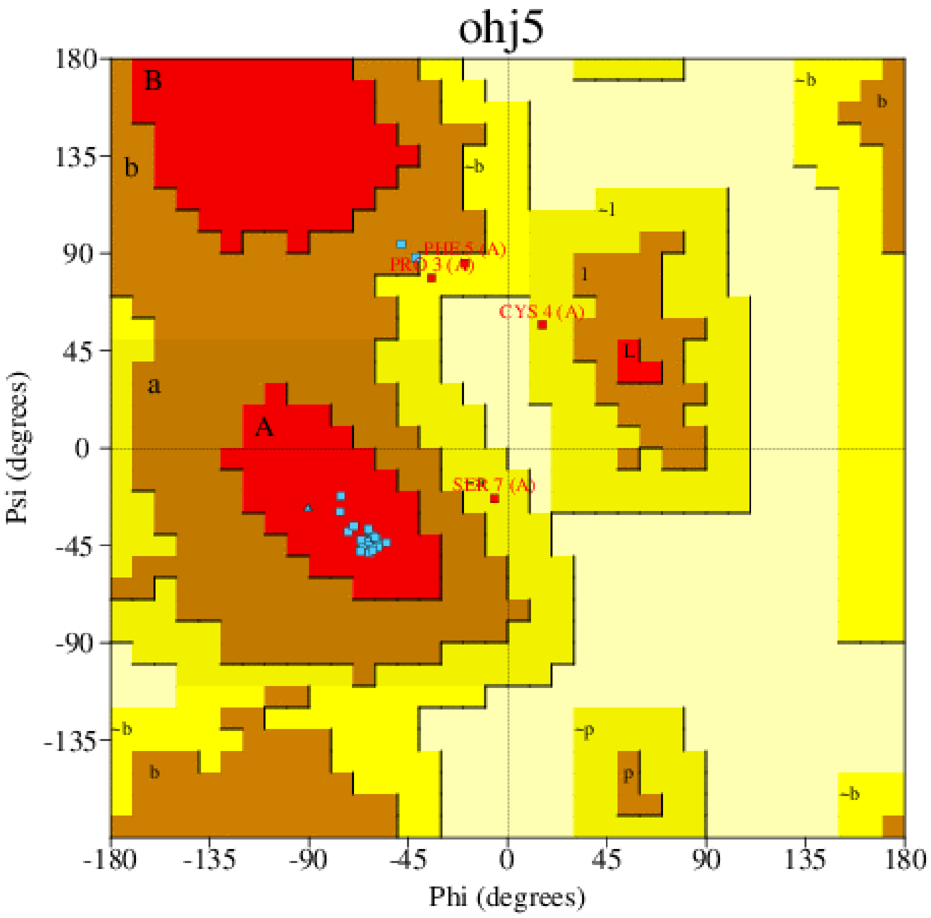

PROCHECK statistics

1. Ramachandran Plot statistics

		No. of residues	%-tage
		------	------
Most favoured regions	[A,B,L]	24	85.7%*

```
Additional allowed regions [a,b,l,p]         1        3.6%
Generously allowed regions [~a,~b,~l,~p]     3       10.7%
Disallowed regions         [XX]              0        0.0%
                                          ----      ------
Non-glycine and non-proline residues        28      100.0%

End-residues (excl. Gly and Pro)             1

Glycine residues                             3
Proline residues                             2
                                          ----
Total number of residues                    34
```

Based on an analysis of **118** structures of resolution of at least **2.0** Angstroms and *R*-factor no greater than **20.0** a good quality model

2. G-Factors

```
                                                       Average
Parameter                                  Score        Score
---------                                  -----        -----
Dihedral angles:-
     Phi-psi distribution                  -0.06
     Chi1-chi2 distribution                -0.70*
     Chi1 only                              0.45
     Chi3 & chi4                            0.65
     Omega                                  0.17
                                                         0.01
                                                        =====
Main-chain covalent forces:-
     Main-chain bond lengths               -0.99*
     Main-chain bond angles                -2.10**
                                                        -1.63**
                                                        =====

     OVERALL AVERAGE                                    -0.69*
                                                        =====
```

G-factors provide a measure of how **unusual**, or out-of-the-ordinary, a property is.

```
Values below -0.5*  - unusual
Values below -1.0** - highly unusual
```

Important note: The main-chain bond-lengths and bond angles are compared with the Engh & Huber (1991) ideal values derived fron
may show apparently large deviations from normality.

Chain A (34 residues)

Secondary structure summary

Strand	Alpha helix	3-10 helix	Other	Total residues
0 (0.0%)	27 (79.4%)	0 (0.0%)	7 (20.6%)	34

1 helix

Start	End	Type	No. resid
Ser7	Gly33	H	27

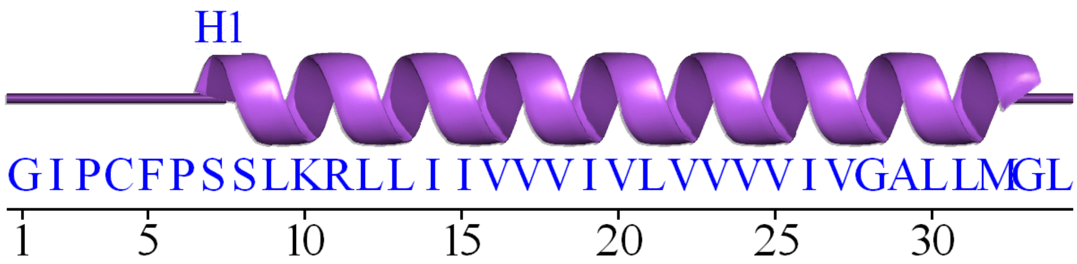

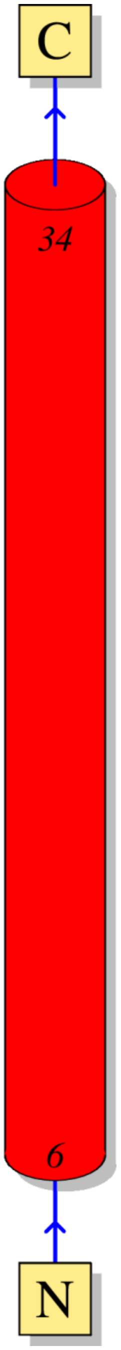

Molecular Topography of the canine SP-C sequence in bilayer membrane. Red cylinder is the sequence that is in the hydrophobic core of the membrane.

Deconvolution of the CD spectrum of canine SP-Cff ion-lock peptide in SDS detergent micelles performed using Dichroweb implementation located at:

Dichroweb (http://dichroweb.cryst.bbk.ac.uk/html/home.shtml)

Selcon 3 (The Self-Consistent Method): Reference dataset: 7 .

Use of the reference set requires the citation of:
Sreerama, N. and Woody, R.W. (2000), Analytical Biochemistry, 287, 252–260 and references therein.

Secondary Structures:

Helix1= Regular Alpha Helix
Helix2= Distorted Alpha Helix
Strand1= Regular Beta Sheet
Strand2= Distorted Beta Sheet
Turns= Turn Structures
Unordered= Random or Disordered Structures

NRMSD:0.049

Result	Helix1	Helix2	Strand1	Strand2	Turns	Unordered	Total
**Guess**	0.529	0.225	0.000	0.000	0.044	0.202	**1**
**SVD**	0.475	0.278	0.128	0.094	0.278	0.451	**1.704**
**Convergent**	0.476	0.202	0.004	0.015	0.119	0.205	**1.021**
**Stage2**	**0.476**	**0.202**	**0.004**	**0.015**	**0.119**	**0.205**	**1.020**
**final**	**0.476**	**0.202**	**0.004**	**0.015**	**0.119**	**0.205**	**1.020**

HELICES (Per 100 Residues) are: 5.040
The AVERAGE LENGTH of HELICES : 13.440

STRANDS (Per 100 Residues) are: 0.756
The AVERAGE LENGTH of STRANDS : 2.491

1: Initial Guess - Closest matching protein: ColA
2: First Stage: Solution based on H&J Method
3: Convergent solution: After iterating with H&J solution for consistency
4: Third Stage: Average of matching Solutions:

Selection rules: Total sum of secondary structures must be >1: determined to be 1.020

Each individual fraction must be > -0.03: observed

The RMSD (NRMSD) between original and reconstructed spectra must be <0.250 : determined to be 0.049

NRMSD (Normalised Root Mean Squared Deviation) indicates the goodness of fit between experimental and calculated structure.

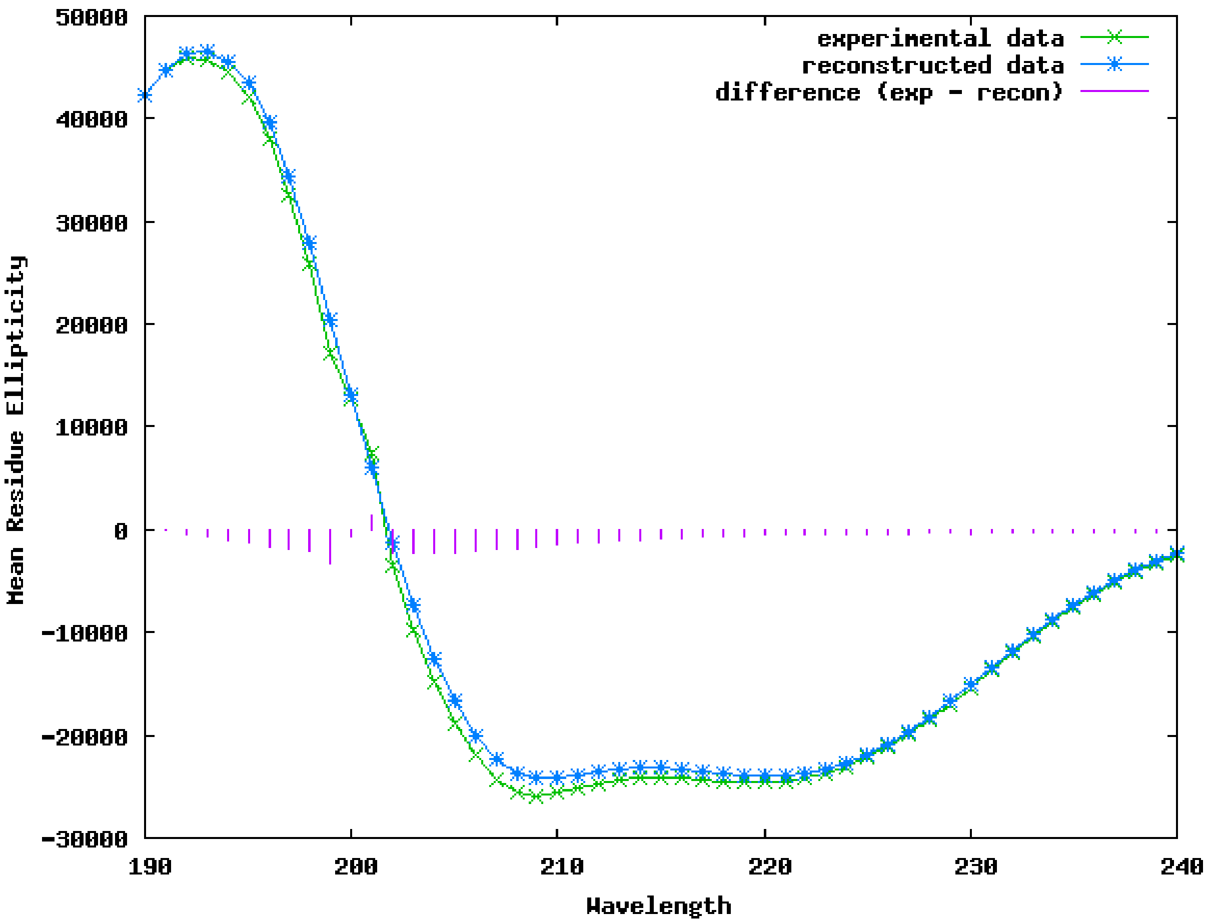

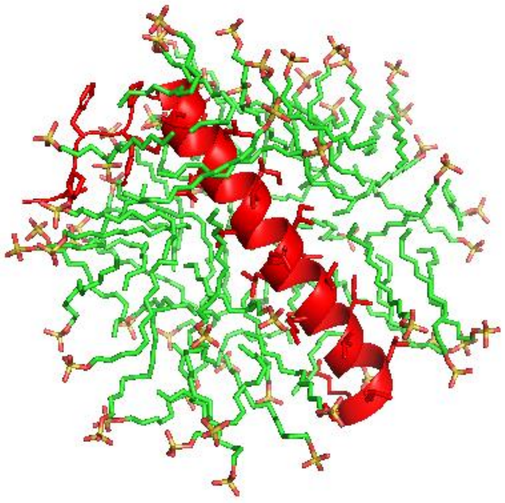

Molecular illustration of canine SP-Cff ion-lock peptide in SDS micelle refined by molecular dynamics. The system was subjected to 10 nano seconds of molecular dynamics as described for other lipid systems in methods section of the manuscript to obtain a representative peptide structure in this environment for the peptide. The SDS micellar structure consisted of 60 SDS molecules with the acyl chains in green and the sulfate polar head groups yellow and orange. Water and counter ions have been removed from the illustration for clarity. The helical domain is highlighted in red ribbon and spans from residue 7 to 31 indicating that the helical component accounts for approximately 70% of the total structure that is consistent with the proportion of helix observed experimentally by CD analysis.
