# Supplementary material for: Structure and Function of Canine SP-C Mimic Proteins in Synthetic Surfactant Lipid Dispersions
Source: Biomedicines. 2024 Jan 12;12(1):163. doi: 10.3390/biomedicines12010163 (PMC10813813; doi:10.3390/biomedicines12010163)

S2 – Secondary Structure Model of Canine SP-Cff ion-lock protein using AlphaFold prediction program.

Article Title: Structure and Function of Canine SP-C Mimic Proteins in Synthetic Surfactant Lipid Dispersions

Frans J. Walther[1,2,*] & Alan J. Waring[1,3]

[1] Lundquist Institute for Biomedical Innovation at Harbor-UCLA Medical Center
1124 West Carson Street
Torrance, CA, USA

[2] Department of Pediatrics
David Geffen School of Medicine
University of California Los Angeles
405 Hilgard Avenue
Los Angeles, CA, USA

[3] Department of Medicine
David Geffen School of Medicine
University of California Los Angeles
405 Hilgard Avenue
Los Angeles, CA, USA

Modeling Protocol

Canine SP-C amino acid sequence downloaded from: https://www.uniprot.org Deposition file: P22397 · PSPC_CANLF. Secondary structure modeling canine SP-C amino acid sequence monomer (Canis lupus familiars) using primary amino acid sequence with the AI based secondary structure prediction program AlphaFold.

Canine SP-C amino acid sequence downloaded from: https://www.uniprot.org Deposition file: P22397 · PSPC_CANLF. The Cysteine residue at position 4 was mutated to phenylalanine to serve as a surrogate for Cys-palmitate in the native sequence. There was also an ion-lock amino acid pair (glutamic acid 20 – lysine 24) placed in the hydrophobic helical sequence to stabilize the helical propensity of the transmembrane domain. The secondary structure of the modified canine SP-C amino acid sequence monomer was then modeled using modified amino acid sequence with the AI based secondary structure prediction program AlphaFold. The AlphaFold program was run through the Chimera X (version 1.6.1) molecular modeling environment at https://www.cgl.ucsf.edu/chimera/docs/relnotes.html.

Jumper J, Evans R, Pritzel A, et al. Highly accurate protein structure prediction with AlphaFold. *Nature*. 2021;596(7873):583-589. doi:10.1038/s41586-021-03819-2
Mirdita M, Schütze K, Moriwaki Y, Heo L, Ovchinnikov S, Steinegger M. ColabFold: making protein folding accessible to all. *Nat Methods*. 2022;19(6):679-682. doi:10.1038/s41592-022-01488-1

Input amino acid sequence for Chimera X:

>SPC_ff_ion_lock_dog

GIPFFPSSLKRLLIIVVVIELVVKVIVGALLMGL

Command Sequence for AlphaFold Prediction of SP-C dog amino acid sequence:

ChimeraX > Structure Prediction > AlphaFold > paste amino acid sequence > Predict

AlphaFold output for Canine SP-C Atomic Coordinate Data Predicted for Canine SP-C Monomer Structure in PDB format:

File: SP-Cff_ion_dog.pdb

```
ATOM      1  N   GLY A   1      -26.977  -4.625  -3.419  1.00 55.34           N
ATOM      2  CA  GLY A   1      -26.637  -4.419  -2.021  1.00 55.34           C
ATOM      3  C   GLY A   1      -25.181  -4.712  -1.712  1.00 55.34           C
ATOM      4  O   GLY A   1      -24.603  -5.655  -2.255  1.00 55.34           O
ATOM      5  N   ILE A   2      -24.183  -3.844  -2.015  1.00 62.84           N
ATOM      6  CA  ILE A   2      -22.778  -4.152  -1.769  1.00 62.84           C
ATOM      7  C   ILE A   2      -22.645  -4.961  -0.480  1.00 62.84           C
ATOM      8  CB  ILE A   2      -21.924  -2.867  -1.685  1.00 62.84           C
ATOM      9  O   ILE A   2      -23.249  -4.620   0.539  1.00 62.84           O
ATOM     10  CG1 ILE A   2      -22.814  -1.625  -1.813  1.00 62.84           C
ATOM     11  CG2 ILE A   2      -20.833  -2.872  -2.759  1.00 62.84           C
ATOM     12  CD1 ILE A   2      -22.189  -0.355  -1.252  1.00 62.84           C
ATOM     13  N   PRO A   3      -22.397  -6.185  -0.533  1.00 59.02           N
ATOM     14  CA  PRO A   3      -22.258  -7.090   0.610  1.00 59.02           C
ATOM     15  C   PRO A   3      -21.448  -6.479   1.751  1.00 59.02           C
ATOM     16  CB  PRO A   3      -21.536  -8.301   0.016  1.00 59.02           C
ATOM     17  O   PRO A   3      -20.522  -5.700   1.508  1.00 59.02           O
ATOM     18  CG  PRO A   3      -21.016  -7.827  -1.303  1.00 59.02           C
ATOM     19  CD  PRO A   3      -21.678  -6.521  -1.634  1.00 59.02           C
ATOM     20  N   PHE A   4      -22.095  -5.777   2.714  1.00 65.41           N
ATOM     21  CA  PHE A   4      -21.612  -5.538   4.068  1.00 65.41           C
ATOM     22  C   PHE A   4      -20.414  -6.427   4.381  1.00 65.41           C
ATOM     23  CB  PHE A   4      -22.727  -5.782   5.089  1.00 65.41           C
ATOM     24  O   PHE A   4      -20.502  -7.652   4.286  1.00 65.41           O
ATOM     25  CG  PHE A   4      -22.498  -5.103   6.413  1.00 65.41           C
ATOM     26  CD1 PHE A   4      -21.963  -5.805   7.486  1.00 65.41           C
ATOM     27  CD2 PHE A   4      -22.818  -3.763   6.583  1.00 65.41           C
ATOM     28  CE1 PHE A   4      -21.750  -5.179   8.712  1.00 65.41           C
ATOM     29  CE2 PHE A   4      -22.608  -3.131   7.806  1.00 65.41           C
ATOM     30  CZ  PHE A   4      -22.075  -3.841   8.869  1.00 65.41           C
ATOM     31  N   PHE A   5      -19.222  -6.139   3.952  1.00 66.07           N
ATOM     32  CA  PHE A   5      -18.008  -6.855   4.325  1.00 66.07           C
ATOM     33  C   PHE A   5      -17.913  -7.007   5.838  1.00 66.07           C
ATOM     34  CB  PHE A   5      -16.769  -6.129   3.792  1.00 66.07           C
ATOM     35  O   PHE A   5      -18.158  -6.054   6.580  1.00 66.07           O
ATOM     36  CG  PHE A   5      -16.636  -6.176   2.294  1.00 66.07           C
ATOM     37  CD1 PHE A   5      -16.116  -7.300   1.663  1.00 66.07           C
ATOM     38  CD2 PHE A   5      -17.030  -5.096   1.515  1.00 66.07           C
ATOM     39  CE1 PHE A   5      -15.992  -7.347   0.276  1.00 66.07           C
ATOM     40  CE2 PHE A   5      -16.909  -5.135   0.129  1.00 66.07           C
ATOM     41  CZ  PHE A   5      -16.389  -6.261  -0.488  1.00 66.07           C
ATOM     42  N   PRO A   6      -18.069  -8.190   6.432  1.00 78.07           N
ATOM     43  CA  PRO A   6      -17.731  -8.384   7.844  1.00 78.07           C
ATOM     44  C   PRO A   6      -16.452  -7.656   8.250  1.00 78.07           C
ATOM     45  CB  PRO A   6      -17.556  -9.900   7.959  1.00 78.07           C
ATOM     46  O   PRO A   6      -15.622  -7.335   7.395  1.00 78.07           O
ATOM     47  CG  PRO A   6      -17.372 -10.372   6.553  1.00 78.07           C
ATOM     48  CD  PRO A   6      -17.900  -9.317   5.623  1.00 78.07           C
ATOM     49  N   SER A   7      -16.446  -6.839   9.236  1.00 81.92           N
ATOM     50  CA  SER A   7      -15.343  -6.102   9.844  1.00 81.92           C
ATOM     51  C   SER A   7      -14.012  -6.811   9.616  1.00 81.92           C
ATOM     52  CB  SER A   7      -15.582  -5.917  11.343  1.00 81.92           C
ATOM     53  O   SER A   7      -13.002  -6.168   9.321  1.00 81.92           O
ATOM     54  OG  SER A   7      -16.787  -5.208  11.576  1.00 81.92           O
ATOM     55  N   SER A   8      -13.903  -8.149   9.612  1.00 84.81           N
```

ATOM	56	CA	SER	A	8	-12.683	-8.930	9.430	1.00	84.81	C
ATOM	57	C	SER	A	8	-12.184	-8.848	7.992	1.00	84.81	C
ATOM	58	CB	SER	A	8	-12.920	-10.391	9.814	1.00	84.81	C
ATOM	59	O	SER	A	8	-10.984	-8.702	7.753	1.00	84.81	O
ATOM	60	OG	SER	A	8	-13.343	-10.495	11.163	1.00	84.81	O
ATOM	61	N	LEU	A	9	-13.199	-8.912	7.074	1.00	87.43	N
ATOM	62	CA	LEU	A	9	-12.806	-8.849	5.671	1.00	87.43	C
ATOM	63	C	LEU	A	9	-12.312	-7.453	5.307	1.00	87.43	C
ATOM	64	CB	LEU	A	9	-13.979	-9.240	4.767	1.00	87.43	C
ATOM	65	O	LEU	A	9	-11.368	-7.307	4.528	1.00	87.43	O
ATOM	66	CG	LEU	A	9	-14.372	-10.718	4.768	1.00	87.43	C
ATOM	67	CD1	LEU	A	9	-15.637	-10.930	3.944	1.00	87.43	C
ATOM	68	CD2	LEU	A	9	-13.230	-11.576	4.235	1.00	87.43	C
ATOM	69	N	LYS	A	10	-13.041	-6.449	5.991	1.00	84.76	N
ATOM	70	CA	LYS	A	10	-12.620	-5.069	5.767	1.00	84.76	C
ATOM	71	C	LYS	A	10	-11.173	-4.858	6.202	1.00	84.76	C
ATOM	72	CB	LYS	A	10	-13.538	-4.100	6.513	1.00	84.76	C
ATOM	73	O	LYS	A	10	-10.393	-4.219	5.492	1.00	84.76	O
ATOM	74	CG	LYS	A	10	-13.214	-2.632	6.275	1.00	84.76	C
ATOM	75	CD	LYS	A	10	-14.311	-1.723	6.814	1.00	84.76	C
ATOM	76	CE	LYS	A	10	-14.396	-0.422	6.027	1.00	84.76	C
ATOM	77	NZ	LYS	A	10	-14.893	0.705	6.873	1.00	84.76	N
ATOM	78	N	ARG	A	11	-10.875	-5.475	7.304	1.00	91.36	N
ATOM	79	CA	ARG	A	11	-9.520	-5.347	7.830	1.00	91.36	C
ATOM	80	C	ARG	A	11	-8.514	-6.058	6.930	1.00	91.36	C
ATOM	81	CB	ARG	A	11	-9.440	-5.907	9.251	1.00	91.36	C
ATOM	82	O	ARG	A	11	-7.435	-5.529	6.658	1.00	91.36	O
ATOM	83	CG	ARG	A	11	-8.085	-5.714	9.914	1.00	91.36	C
ATOM	84	CD	ARG	A	11	-8.096	-6.175	11.365	1.00	91.36	C
ATOM	85	NE	ARG	A	11	-8.708	-5.182	12.242	1.00	91.36	N
ATOM	86	NH1	ARG	A	11	-8.328	-6.364	14.190	1.00	91.36	N
ATOM	87	NH2	ARG	A	11	-9.382	-4.330	14.266	1.00	91.36	N
ATOM	88	CZ	ARG	A	11	-8.805	-5.294	13.564	1.00	91.36	C
ATOM	89	N	LEU	A	12	-8.813	-7.099	6.458	1.00	94.39	N
ATOM	90	CA	LEU	A	12	-7.951	-7.866	5.564	1.00	94.39	C
ATOM	91	C	LEU	A	12	-7.693	-7.102	4.270	1.00	94.39	C
ATOM	92	CB	LEU	A	12	-8.579	-9.226	5.251	1.00	94.39	C
ATOM	93	O	LEU	A	12	-6.554	-7.027	3.805	1.00	94.39	O
ATOM	94	CG	LEU	A	12	-7.749	-10.172	4.382	1.00	94.39	C
ATOM	95	CD1	LEU	A	12	-6.467	-10.569	5.106	1.00	94.39	C
ATOM	96	CD2	LEU	A	12	-8.562	-11.407	4.007	1.00	94.39	C
ATOM	97	N	LEU	A	13	-8.638	-6.522	3.720	1.00	94.70	N
ATOM	98	CA	LEU	A	13	-8.537	-5.794	2.459	1.00	94.70	C
ATOM	99	C	LEU	A	13	-7.564	-4.626	2.583	1.00	94.70	C
ATOM	100	CB	LEU	A	13	-9.912	-5.285	2.022	1.00	94.70	C
ATOM	101	O	LEU	A	13	-6.732	-4.410	1.699	1.00	94.70	O
ATOM	102	CG	LEU	A	13	-10.660	-6.143	1.000	1.00	94.70	C
ATOM	103	CD1	LEU	A	13	-12.166	-5.964	1.158	1.00	94.70	C
ATOM	104	CD2	LEU	A	13	-10.220	-5.791	-0.417	1.00	94.70	C
ATOM	105	N	ILE	A	14	-7.678	-3.965	3.673	1.00	96.03	N
ATOM	106	CA	ILE	A	14	-6.836	-2.798	3.910	1.00	96.03	C
ATOM	107	C	ILE	A	14	-5.370	-3.222	3.968	1.00	96.03	C
ATOM	108	CB	ILE	A	14	-7.235	-2.069	5.213	1.00	96.03	C
ATOM	109	O	ILE	A	14	-4.508	-2.582	3.362	1.00	96.03	O
ATOM	110	CG1	ILE	A	14	-8.646	-1.484	5.088	1.00	96.03	C
ATOM	111	CG2	ILE	A	14	-6.217	-0.976	5.554	1.00	96.03	C
ATOM	112	CD1	ILE	A	14	-9.238	-1.006	6.406	1.00	96.03	C
ATOM	113	N	ILE	A	15	-5.163	-4.326	4.639	1.00	97.18	N
ATOM	114	CA	ILE	A	15	-3.804	-4.831	4.801	1.00	97.18	C
ATOM	115	C	ILE	A	15	-3.220	-5.189	3.437	1.00	97.18	C
ATOM	116	CB	ILE	A	15	-3.766	-6.058	5.740	1.00	97.18	C
ATOM	117	O	ILE	A	15	-2.084	-4.823	3.125	1.00	97.18	O
ATOM	118	CG1	ILE	A	15	-4.158	-5.652	7.165	1.00	97.18	C

ATOM	119	CG2	ILE	A	15	-2.381	-6.712	5.718	1.00	97.18	C
ATOM	120	CD1	ILE	A	15	-4.390	-6.830	8.102	1.00	97.18	C
ATOM	121	N	VAL	A	16	-4.015	-5.818	2.688	1.00	96.91	N
ATOM	122	CA	VAL	A	16	-3.575	-6.261	1.369	1.00	96.91	C
ATOM	123	C	VAL	A	16	-3.279	-5.050	0.488	1.00	96.91	C
ATOM	124	CB	VAL	A	16	-4.630	-7.166	0.694	1.00	96.91	C
ATOM	125	O	VAL	A	16	-2.272	-5.022	-0.222	1.00	96.91	O
ATOM	126	CG1	VAL	A	16	-4.315	-7.349	-0.789	1.00	96.91	C
ATOM	127	CG2	VAL	A	16	-4.702	-8.519	1.399	1.00	96.91	C
ATOM	128	N	VAL	A	17	-4.088	-4.097	0.610	1.00	97.15	N
ATOM	129	CA	VAL	A	17	-3.941	-2.885	-0.189	1.00	97.15	C
ATOM	130	C	VAL	A	17	-2.649	-2.167	0.194	1.00	97.15	C
ATOM	131	CB	VAL	A	17	-5.149	-1.938	-0.012	1.00	97.15	C
ATOM	132	O	VAL	A	17	-1.888	-1.738	-0.676	1.00	97.15	O
ATOM	133	CG1	VAL	A	17	-4.834	-0.551	-0.569	1.00	97.15	C
ATOM	134	CG2	VAL	A	17	-6.387	-2.521	-0.691	1.00	97.15	C
ATOM	135	N	VAL	A	18	-2.518	-2.099	1.435	1.00	97.55	N
ATOM	136	CA	VAL	A	18	-1.328	-1.418	1.935	1.00	97.55	C
ATOM	137	C	VAL	A	18	-0.076	-2.165	1.481	1.00	97.55	C
ATOM	138	CB	VAL	A	18	-1.348	-1.297	3.475	1.00	97.55	C
ATOM	139	O	VAL	A	18	0.878	-1.553	0.996	1.00	97.55	O
ATOM	140	CG1	VAL	A	18	-0.020	-0.746	3.991	1.00	97.55	C
ATOM	141	CG2	VAL	A	18	-2.508	-0.411	3.925	1.00	97.55	C
ATOM	142	N	ILE	A	19	-0.093	-3.425	1.649	1.00	98.28	N
ATOM	143	CA	ILE	A	19	1.047	-4.250	1.265	1.00	98.28	C
ATOM	144	C	ILE	A	19	1.294	-4.123	-0.236	1.00	98.28	C
ATOM	145	CB	ILE	A	19	0.827	-5.731	1.649	1.00	98.28	C
ATOM	146	O	ILE	A	19	2.438	-3.980	-0.673	1.00	98.28	O
ATOM	147	CG1	ILE	A	19	0.826	-5.893	3.173	1.00	98.28	C
ATOM	148	CG2	ILE	A	19	1.895	-6.620	1.004	1.00	98.28	C
ATOM	149	CD1	ILE	A	19	0.434	-7.286	3.647	1.00	98.28	C
ATOM	150	N	GLU	A	20	0.220	-4.236	-1.008	1.00	97.65	N
ATOM	151	CA	GLU	A	20	0.358	-4.114	-2.456	1.00	97.65	C
ATOM	152	C	GLU	A	20	0.995	-2.781	-2.840	1.00	97.65	C
ATOM	153	CB	GLU	A	20	-1.002	-4.263	-3.141	1.00	97.65	C
ATOM	154	O	GLU	A	20	1.845	-2.727	-3.731	1.00	97.65	O
ATOM	155	CG	GLU	A	20	-1.378	-5.704	-3.457	1.00	97.65	C
ATOM	156	CD	GLU	A	20	-0.885	-6.168	-4.818	1.00	97.65	C
ATOM	157	OE1	GLU	A	20	0.102	-6.937	-4.876	1.00	97.65	O
ATOM	158	OE2	GLU	A	20	-1.489	-5.759	-5.834	1.00	97.65	O
ATOM	159	N	LEU	A	21	0.623	-1.820	-2.188	1.00	98.27	N
ATOM	160	CA	LEU	A	21	1.142	-0.480	-2.441	1.00	98.27	C
ATOM	161	C	LEU	A	21	2.629	-0.403	-2.111	1.00	98.27	C
ATOM	162	CB	LEU	A	21	0.371	0.557	-1.621	1.00	98.27	C
ATOM	163	O	LEU	A	21	3.414	0.144	-2.889	1.00	98.27	O
ATOM	164	CG	LEU	A	21	0.664	2.025	-1.936	1.00	98.27	C
ATOM	165	CD1	LEU	A	21	0.030	2.418	-3.266	1.00	98.27	C
ATOM	166	CD2	LEU	A	21	0.163	2.924	-0.812	1.00	98.27	C
ATOM	167	N	VAL	A	22	2.983	-0.937	-0.969	1.00	97.75	N
ATOM	168	CA	VAL	A	22	4.372	-0.923	-0.523	1.00	97.75	C
ATOM	169	C	VAL	A	22	5.238	-1.713	-1.501	1.00	97.75	C
ATOM	170	CB	VAL	A	22	4.516	-1.500	0.903	1.00	97.75	C
ATOM	171	O	VAL	A	22	6.312	-1.256	-1.899	1.00	97.75	O
ATOM	172	CG1	VAL	A	22	5.989	-1.639	1.283	1.00	97.75	C
ATOM	173	CG2	VAL	A	22	3.782	-0.619	1.912	1.00	97.75	C
ATOM	174	N	VAL	A	23	4.825	-2.784	-1.892	1.00	97.68	N
ATOM	175	CA	VAL	A	23	5.569	-3.653	-2.798	1.00	97.68	C
ATOM	176	C	VAL	A	23	5.770	-2.950	-4.139	1.00	97.68	C
ATOM	177	CB	VAL	A	23	4.850	-5.005	-3.007	1.00	97.68	C
ATOM	178	O	VAL	A	23	6.860	-2.996	-4.714	1.00	97.68	O
ATOM	179	CG1	VAL	A	23	5.531	-5.814	-4.110	1.00	97.68	C
ATOM	180	CG2	VAL	A	23	4.817	-5.797	-1.702	1.00	97.68	C
ATOM	181	N	LYS	A	24	4.710	-2.315	-4.584	1.00	96.92	N

ATOM	182	CA	LYS	A	24	4.799	-1.603	-5.856	1.00	96.92	C
ATOM	183	C	LYS	A	24	5.839	-0.488	-5.790	1.00	96.92	C
ATOM	184	CB	LYS	A	24	3.437	-1.027	-6.245	1.00	96.92	C
ATOM	185	O	LYS	A	24	6.567	-0.254	-6.757	1.00	96.92	O
ATOM	186	CG	LYS	A	24	2.536	-2.010	-6.980	1.00	96.92	C
ATOM	187	CD	LYS	A	24	1.153	-1.422	-7.228	1.00	96.92	C
ATOM	188	CE	LYS	A	24	0.331	-2.300	-8.162	1.00	96.92	C
ATOM	189	NZ	LYS	A	24	-1.109	-1.904	-8.169	1.00	96.92	N
ATOM	190	N	VAL	A	25	5.869	0.166	-4.721	1.00	96.75	N
ATOM	191	CA	VAL	A	25	6.843	1.236	-4.529	1.00	96.75	C
ATOM	192	C	VAL	A	25	8.255	0.656	-4.528	1.00	96.75	C
ATOM	193	CB	VAL	A	25	6.583	2.011	-3.218	1.00	96.75	C
ATOM	194	O	VAL	A	25	9.151	1.185	-5.191	1.00	96.75	O
ATOM	195	CG1	VAL	A	25	7.725	2.985	-2.931	1.00	96.75	C
ATOM	196	CG2	VAL	A	25	5.250	2.753	-3.292	1.00	96.75	C
ATOM	197	N	ILE	A	26	8.394	-0.427	-3.829	1.00	97.05	N
ATOM	198	CA	ILE	A	26	9.712	-1.043	-3.714	1.00	97.05	C
ATOM	199	C	ILE	A	26	10.164	-1.553	-5.080	1.00	97.05	C
ATOM	200	CB	ILE	A	26	9.709	-2.195	-2.685	1.00	97.05	C
ATOM	201	O	ILE	A	26	11.306	-1.328	-5.488	1.00	97.05	O
ATOM	202	CG1	ILE	A	26	9.401	-1.658	-1.282	1.00	97.05	C
ATOM	203	CG2	ILE	A	26	11.048	-2.939	-2.704	1.00	97.05	C
ATOM	204	CD1	ILE	A	26	9.268	-2.741	-0.220	1.00	97.05	C
ATOM	205	N	VAL	A	27	9.282	-2.207	-5.695	1.00	96.52	N
ATOM	206	CA	VAL	A	27	9.597	-2.753	-7.011	1.00	96.52	C
ATOM	207	C	VAL	A	27	9.889	-1.615	-7.986	1.00	96.52	C
ATOM	208	CB	VAL	A	27	8.447	-3.634	-7.549	1.00	96.52	C
ATOM	209	O	VAL	A	27	10.829	-1.697	-8.781	1.00	96.52	O
ATOM	210	CG1	VAL	A	27	8.727	-4.066	-8.988	1.00	96.52	C
ATOM	211	CG2	VAL	A	27	8.244	-4.853	-6.651	1.00	96.52	C
ATOM	212	N	GLY	A	28	9.082	-0.614	-7.880	1.00	96.83	N
ATOM	213	CA	GLY	A	28	9.296	0.556	-8.716	1.00	96.83	C
ATOM	214	C	GLY	A	28	10.641	1.217	-8.485	1.00	96.83	C
ATOM	215	O	GLY	A	28	11.312	1.622	-9.437	1.00	96.83	O
ATOM	216	N	ALA	A	29	11.039	1.248	-7.281	1.00	96.13	N
ATOM	217	CA	ALA	A	29	12.322	1.844	-6.917	1.00	96.13	C
ATOM	218	C	ALA	A	29	13.485	0.996	-7.425	1.00	96.13	C
ATOM	219	CB	ALA	A	29	12.417	2.019	-5.403	1.00	96.13	C
ATOM	220	O	ALA	A	29	14.502	1.531	-7.872	1.00	96.13	O
ATOM	221	N	LEU	A	30	13.298	-0.293	-7.412	1.00	94.96	N
ATOM	222	CA	LEU	A	30	14.331	-1.228	-7.845	1.00	94.96	C
ATOM	223	C	LEU	A	30	14.546	-1.141	-9.352	1.00	94.96	C
ATOM	224	CB	LEU	A	30	13.956	-2.660	-7.453	1.00	94.96	C
ATOM	225	O	LEU	A	30	15.682	-1.211	-9.826	1.00	94.96	O
ATOM	226	CG	LEU	A	30	14.104	-3.019	-5.974	1.00	94.96	C
ATOM	227	CD1	LEU	A	30	13.431	-4.356	-5.683	1.00	94.96	C
ATOM	228	CD2	LEU	A	30	15.575	-3.058	-5.577	1.00	94.96	C
ATOM	229	N	LEU	A	31	13.386	-0.878	-10.070	1.00	94.76	N
ATOM	230	CA	LEU	A	31	13.458	-0.825	-11.526	1.00	94.76	C
ATOM	231	C	LEU	A	31	14.066	0.494	-11.992	1.00	94.76	C
ATOM	232	CB	LEU	A	31	12.066	-1.003	-12.139	1.00	94.76	C
ATOM	233	O	LEU	A	31	14.713	0.548	-13.040	1.00	94.76	O
ATOM	234	CG	LEU	A	31	11.430	-2.386	-11.987	1.00	94.76	C
ATOM	235	CD1	LEU	A	31	10.011	-2.381	-12.545	1.00	94.76	C
ATOM	236	CD2	LEU	A	31	12.281	-3.445	-12.681	1.00	94.76	C
ATOM	237	N	MET	A	32	13.833	1.494	-11.140	1.00	93.08	N
ATOM	238	CA	MET	A	32	14.332	2.811	-11.523	1.00	93.08	C
ATOM	239	C	MET	A	32	15.789	2.983	-11.107	1.00	93.08	C
ATOM	240	CB	MET	A	32	13.476	3.914	-10.899	1.00	93.08	C
ATOM	241	O	MET	A	32	16.483	3.868	-11.611	1.00	93.08	O
ATOM	242	CG	MET	A	32	12.920	4.905	-11.909	1.00	93.08	C
ATOM	243	SD	MET	A	32	11.131	4.654	-12.230	1.00	93.08	S
ATOM	244	CE	MET	A	32	10.794	6.080	-13.299	1.00	93.08	C

```
ATOM    245  N   GLY A  33      16.538   1.864 -10.684  1.00 85.12           N
ATOM    246  CA  GLY A  33      17.967   1.922 -10.419  1.00 85.12           C
ATOM    247  C   GLY A  33      18.331   2.895  -9.314  1.00 85.12           C
ATOM    248  O   GLY A  33      19.480   3.330  -9.216  1.00 85.12           O
ATOM    249  N   LEU A  34      17.286   3.355  -8.550  1.00 68.36           N
ATOM    250  CA  LEU A  34      17.706   4.114  -7.376  1.00 68.36           C
ATOM    251  C   LEU A  34      18.452   3.221  -6.390  1.00 68.36           C
ATOM    252  CB  LEU A  34      16.495   4.752  -6.690  1.00 68.36           C
ATOM    253  O   LEU A  34      19.460   3.633  -5.813  1.00 68.36           O
ATOM    254  CG  LEU A  34      15.912   5.995  -7.363  1.00 68.36           C
ATOM    255  CD1 LEU A  34      14.503   6.267  -6.845  1.00 68.36           C
ATOM    256  CD2 LEU A  34      16.814   7.203  -7.130  1.00 68.36           C
CONECT  249  250  247
CONECT  250  249  252  251
CONECT  251  250  253
CONECT  252  250  254
CONECT  253  251
CONECT  254  252  255  256
CONECT  255  254
CONECT  256  254
END


ATOM      2  HT1 GLY     1      60.059  37.915  37.310  1.00  0.00      PROA H
ATOM      3  HT2 GLY     1      58.966  38.418  36.105  1.00  0.00      PROA H
ATOM      4  HT3 GLY     1      60.382  39.314  36.410  1.00  0.00      PROA H
ATOM      5  CA  GLY     1      60.705  37.538  35.341  1.00  0.00      PROA C
ATOM      6  HA1 GLY     1      60.240  36.560  35.317  1.00  0.00      PROA H
ATOM      7  HA2 GLY     1      61.744  37.495  35.639  1.00  0.00      PROA H
ATOM      8  C   GLY     1      60.653  38.104  33.944  1.00  0.00      PROA C
ATOM      9  O   GLY     1      61.230  37.538  33.031  1.00  0.00      PROA O
ATOM     10  N   ILE     2      59.955  39.247  33.759  1.00  0.00      PROA N
ATOM     11  HN  ILE     2      59.532  39.751  34.496  1.00  0.00      PROA H
ATOM     12  CA  ILE     2      59.692  39.837  32.458  1.00  0.00      PROA C
ATOM     13  HA  ILE     2      60.213  39.318  31.673  1.00  0.00      PROA H
ATOM     14  CB  ILE     2      58.236  39.860  32.111  1.00  0.00      PROA C
ATOM     15  HB  ILE     2      57.628  40.418  32.872  1.00  0.00      PROA H
ATOM     16  CG2 ILE     2      57.906  40.427  30.714  1.00  0.00      PROA C
ATOM     17 HG21 ILE     2      58.568  39.971  29.950  1.00  0.00      PROA H
ATOM     18 HG22 ILE     2      58.013  41.529  30.706  1.00  0.00      PROA H
ATOM     19 HG23 ILE     2      56.852  40.192  30.435  1.00  0.00      PROA H
ATOM     20  CG1 ILE     2      57.753  38.444  32.153  1.00  0.00      PROA C
ATOM     21 HG11 ILE     2      56.719  38.666  31.826  1.00  0.00      PROA H
ATOM     22 HG12 ILE     2      57.768  38.055  33.196  1.00  0.00      PROA H
ATOM     23  CD  ILE     2      58.294  37.394  31.168  1.00  0.00      PROA C
ATOM     24  HD1 ILE     2      59.368  37.213  31.318  1.00  0.00      PROA H
ATOM     25  HD2 ILE     2      58.078  37.711  30.127  1.00  0.00      PROA H
ATOM     26  HD3 ILE     2      57.774  36.413  31.283  1.00  0.00      PROA H
ATOM     27  C   ILE     2      60.196  41.216  32.458  1.00  0.00      PROA C
ATOM     28  O   ILE     2      59.469  42.192  32.683  1.00  0.00      PROA O
ATOM     29  N   PRO     3      61.487  41.343  32.212  1.00  0.00      PROA N
ATOM     30  CD  PRO     3      62.544  40.329  32.153  1.00  0.00      PROA C
ATOM     31  HD1 PRO     3      62.246  39.585  31.406  1.00  0.00      PROA H
ATOM     32  HD2 PRO     3      62.740  39.917  33.169  1.00  0.00      PROA H
ATOM     33  CA  PRO     3      62.035  42.612  32.149  1.00  0.00      PROA C
ATOM     34  HA  PRO     3      61.573  43.058  33.013  1.00  0.00      PROA H
ATOM     35  CB  PRO     3      63.557  42.345  32.211  1.00  0.00      PROA C
ATOM     36  HB1 PRO     3      63.972  42.439  33.249  1.00  0.00      PROA H
ATOM     37  HB2 PRO     3      64.088  43.004  31.503  1.00  0.00      PROA H
ATOM     38  CG  PRO     3      63.806  41.008  31.667  1.00  0.00      PROA C
ATOM     39  HG1 PRO     3      63.859  41.162  30.563  1.00  0.00      PROA H
ATOM     40  HG2 PRO     3      64.780  40.641  32.062  1.00  0.00      PROA H
ATOM     41  C   PRO     3      61.619  43.468  30.951  1.00  0.00      PROA C
```

ATOM	42	O	PRO	3	61.738	44.684	30.896	1.00	0.00	PROA	O
ATOM	43	N	CYSP	4	61.069	42.834	29.961	1.00	0.00	PROA	N
ATOM	44	HN	CYSP	4	60.982	41.858	30.083	1.00	0.00	PROA	H
ATOM	45	CA	CYSP	4	60.579	43.451	28.760	1.00	0.00	PROA	C
ATOM	46	HA	CYSP	4	61.340	44.131	28.392	1.00	0.00	PROA	H
ATOM	47	C	CYSP	4	59.297	44.285	28.939	1.00	0.00	PROA	C
ATOM	48	O	CYSP	4	58.680	44.640	27.940	1.00	0.00	PROA	O
ATOM	49	CB	CYSP	4	60.370	42.347	27.689	1.00	0.00	PROA	C
ATOM	50	HB1	CYSP	4	59.620	41.615	28.065	1.00	0.00	PROA	H
ATOM	51	HB2	CYSP	4	59.930	42.823	26.790	1.00	0.00	PROA	H
ATOM	52	SG	CYSP	4	61.935	41.521	27.165	1.00	0.00	PROA	S
ATOM	53	C1	CYSP	4	61.957	40.010	28.180	1.00	0.00	PROA	C
ATOM	54	O1	CYSP	4	61.111	39.843	29.046	1.00	0.00	PROA	O
ATOM	55	C2	CYSP	4	63.077	39.002	27.873	1.00	0.00	PROA	C
ATOM	56	H2A	CYSP	4	64.058	39.522	27.900	1.00	0.00	PROA	H
ATOM	57	H2B	CYSP	4	62.936	38.593	26.850	1.00	0.00	PROA	H
ATOM	58	C3	CYSP	4	62.930	37.933	28.985	1.00	0.00	PROA	C
ATOM	59	H3A	CYSP	4	61.971	37.384	28.857	1.00	0.00	PROA	H
ATOM	60	H3B	CYSP	4	63.016	38.425	29.971	1.00	0.00	PROA	H
ATOM	61	C4	CYSP	4	63.947	36.841	29.148	1.00	0.00	PROA	C
ATOM	62	H4A	CYSP	4	64.922	37.350	29.345	1.00	0.00	PROA	H
ATOM	63	H4B	CYSP	4	63.785	36.274	28.208	1.00	0.00	PROA	H
ATOM	64	C5	CYSP	4	63.844	36.028	30.428	1.00	0.00	PROA	C
ATOM	65	H5A	CYSP	4	62.841	35.595	30.423	1.00	0.00	PROA	H
ATOM	66	H5B	CYSP	4	64.183	36.743	31.216	1.00	0.00	PROA	H
ATOM	67	C6	CYSP	4	64.708	34.808	30.627	1.00	0.00	PROA	C
ATOM	68	H6A	CYSP	4	65.543	35.439	30.451	1.00	0.00	PROA	H
ATOM	69	H6B	CYSP	4	64.546	34.113	29.798	1.00	0.00	PROA	H
ATOM	70	C7	CYSP	4	65.546	34.478	31.877	1.00	0.00	PROA	C
ATOM	71	H7A	CYSP	4	64.701	34.303	32.558	1.00	0.00	PROA	H
ATOM	72	H7B	CYSP	4	66.170	35.350	32.208	1.00	0.00	PROA	H
ATOM	73	C8	CYSP	4	66.489	33.252	31.870	1.00	0.00	PROA	C
ATOM	74	H8A	CYSP	4	67.128	33.005	31.011	1.00	0.00	PROA	H
ATOM	75	H8B	CYSP	4	65.756	32.524	31.639	1.00	0.00	PROA	H
ATOM	76	C9	CYSP	4	67.281	33.113	33.197	1.00	0.00	PROA	C
ATOM	77	H9A	CYSP	4	66.632	33.497	34.012	1.00	0.00	PROA	H
ATOM	78	H9B	CYSP	4	68.145	33.799	33.134	1.00	0.00	PROA	H
ATOM	79	C10	CYSP	4	67.798	31.712	33.609	1.00	0.00	PROA	C
ATOM	80	H10A	CYSP	4	68.350	31.241	32.767	1.00	0.00	PROA	H
ATOM	81	H10B	CYSP	4	66.912	31.084	33.836	1.00	0.00	PROA	H
ATOM	82	C11	CYSP	4	68.713	31.698	34.864	1.00	0.00	PROA	C
ATOM	83	H11A	CYSP	4	68.297	32.406	35.615	1.00	0.00	PROA	H
ATOM	84	H11B	CYSP	4	69.727	32.059	34.586	1.00	0.00	PROA	H
ATOM	85	C12	CYSP	4	68.831	30.308	35.530	1.00	0.00	PROA	C
ATOM	86	H12A	CYSP	4	69.227	29.571	34.797	1.00	0.00	PROA	H
ATOM	87	H12B	CYSP	4	67.808	29.982	35.826	1.00	0.00	PROA	H
ATOM	88	C13	CYSP	4	69.721	30.253	36.783	1.00	0.00	PROA	C
ATOM	89	H13A	CYSP	4	69.501	31.121	37.446	1.00	0.00	PROA	H
ATOM	90	H13B	CYSP	4	70.798	30.295	36.509	1.00	0.00	PROA	H
ATOM	91	C14	CYSP	4	69.511	28.986	37.612	1.00	0.00	PROA	C
ATOM	92	H14A	CYSP	4	69.726	28.063	37.036	1.00	0.00	PROA	H
ATOM	93	H14B	CYSP	4	68.433	28.947	37.901	1.00	0.00	PROA	H
ATOM	94	C15	CYSP	4	70.477	29.011	38.792	1.00	0.00	PROA	C
ATOM	95	H15A	CYSP	4	70.259	29.921	39.426	1.00	0.00	PROA	H
ATOM	96	H15B	CYSP	4	71.538	28.971	38.521	1.00	0.00	PROA	H
ATOM	97	C16	CYSP	4	70.427	27.816	39.661	1.00	0.00	PROA	C
ATOM	98	H16A	CYSP	4	69.596	28.211	40.047	1.00	0.00	PROA	H
ATOM	99	H16B	CYSP	4	69.982	26.846	39.364	1.00	0.00	PROA	H
ATOM	100	H16C	CYSP	4	70.646	28.008	40.713	1.00	0.00	PROA	H
ATOM	101	N	PHE	5	58.904	44.628	30.203	1.00	0.00	PROA	N
ATOM	102	HN	PHE	5	59.362	44.157	30.953	1.00	0.00	PROA	H
ATOM	103	CA	PHE	5	57.975	45.673	30.635	1.00	0.00	PROA	C
ATOM	104	HA	PHE	5	58.507	46.188	31.418	1.00	0.00	PROA	H

ATOM	105	CB	PHE	5	57.597	46.713	29.532	1.00	0.00	PROA	C
ATOM	106	HB1	PHE	5	56.873	46.253	28.826	1.00	0.00	PROA	H
ATOM	107	HB2	PHE	5	58.512	46.980	28.963	1.00	0.00	PROA	H
ATOM	108	CG	PHE	5	57.003	47.993	30.063	1.00	0.00	PROA	C
ATOM	109	CD1	PHE	5	57.848	49.039	30.474	1.00	0.00	PROA	C
ATOM	110	HD1	PHE	5	58.919	48.905	30.428	1.00	0.00	PROA	H
ATOM	111	CE1	PHE	5	57.315	50.242	30.952	1.00	0.00	PROA	C
ATOM	112	HE1	PHE	5	57.975	51.038	31.266	1.00	0.00	PROA	H
ATOM	113	CZ	PHE	5	55.926	50.413	31.018	1.00	0.00	PROA	C
ATOM	114	HZ	PHE	5	55.514	51.344	31.377	1.00	0.00	PROA	H
ATOM	115	CD2	PHE	5	55.612	48.181	30.128	1.00	0.00	PROA	C
ATOM	116	HD2	PHE	5	54.954	47.389	29.812	1.00	0.00	PROA	H
ATOM	117	CE2	PHE	5	55.072	49.379	30.611	1.00	0.00	PROA	C
ATOM	118	HE2	PHE	5	53.999	49.507	30.661	1.00	0.00	PROA	H
ATOM	119	C	PHE	5	56.722	45.063	31.261	1.00	0.00	PROA	C
ATOM	120	O	PHE	5	56.214	44.069	30.738	1.00	0.00	PROA	O
ATOM	121	N	PRO	6	56.162	45.592	32.365	1.00	0.00	PROA	N
ATOM	122	CD	PRO	6	56.770	46.639	33.198	1.00	0.00	PROA	C
ATOM	123	HD1	PRO	6	57.044	47.525	32.594	1.00	0.00	PROA	H
ATOM	124	HD2	PRO	6	57.676	46.236	33.703	1.00	0.00	PROA	H
ATOM	125	CA	PRO	6	54.908	45.113	32.936	1.00	0.00	PROA	C
ATOM	126	HA	PRO	6	54.894	44.035	32.957	1.00	0.00	PROA	H
ATOM	127	CB	PRO	6	54.904	45.717	34.362	1.00	0.00	PROA	C
ATOM	128	HB1	PRO	6	55.434	45.028	35.057	1.00	0.00	PROA	H
ATOM	129	HB2	PRO	6	53.889	45.897	34.768	1.00	0.00	PROA	H
ATOM	130	CG	PRO	6	55.711	47.011	34.233	1.00	0.00	PROA	C
ATOM	131	HG1	PRO	6	55.057	47.819	33.833	1.00	0.00	PROA	H
ATOM	132	HG2	PRO	6	56.158	47.341	35.192	1.00	0.00	PROA	H
ATOM	133	C	PRO	6	53.701	45.547	32.110	1.00	0.00	PROA	C
ATOM	134	O	PRO	6	52.848	46.281	32.584	1.00	0.00	PROA	O
ATOM	135	N	SER	7	53.603	45.098	30.853	1.00	0.00	PROA	N
ATOM	136	HN	SER	7	54.343	44.508	30.515	1.00	0.00	PROA	H
ATOM	137	CA	SER	7	52.591	45.507	29.894	1.00	0.00	PROA	C
ATOM	138	HA	SER	7	52.363	46.553	30.024	1.00	0.00	PROA	H
ATOM	139	CB	SER	7	53.133	45.267	28.464	1.00	0.00	PROA	C
ATOM	140	HB1	SER	7	53.436	44.205	28.327	1.00	0.00	PROA	H
ATOM	141	HB2	SER	7	54.044	45.891	28.324	1.00	0.00	PROA	H
ATOM	142	OG	SER	7	52.174	45.614	27.477	1.00	0.00	PROA	O
ATOM	143	HG1	SER	7	52.632	45.596	26.632	1.00	0.00	PROA	H
ATOM	144	C	SER	7	51.286	44.793	30.132	1.00	0.00	PROA	C
ATOM	145	O	SER	7	50.346	45.280	30.754	1.00	0.00	PROA	O
ATOM	146	N	SER	8	51.213	43.545	29.702	1.00	0.00	PROA	N
ATOM	147	HN	SER	8	51.905	43.124	29.123	1.00	0.00	PROA	H
ATOM	148	CA	SER	8	50.201	42.646	30.100	1.00	0.00	PROA	C
ATOM	149	HA	SER	8	49.232	42.995	29.753	1.00	0.00	PROA	H
ATOM	150	CB	SER	8	50.624	41.396	29.340	1.00	0.00	PROA	C
ATOM	151	HB1	SER	8	50.426	41.537	28.252	1.00	0.00	PROA	H
ATOM	152	HB2	SER	8	50.122	40.537	29.654	1.00	0.00	PROA	H
ATOM	153	OG	SER	8	51.888	40.842	29.589	1.00	0.00	PROA	O
ATOM	154	HG1	SER	8	51.797	39.967	29.183	1.00	0.00	PROA	H
ATOM	155	C	SER	8	50.091	42.492	31.618	1.00	0.00	PROA	C
ATOM	156	O	SER	8	49.032	42.456	32.207	1.00	0.00	PROA	O
ATOM	157	N	LEU	9	51.175	42.505	32.351	1.00	0.00	PROA	N
ATOM	158	HN	LEU	9	52.046	42.603	31.893	1.00	0.00	PROA	H
ATOM	159	CA	LEU	9	51.149	42.506	33.795	1.00	0.00	PROA	C
ATOM	160	HA	LEU	9	50.566	41.659	34.134	1.00	0.00	PROA	H
ATOM	161	CB	LEU	9	52.582	42.468	34.334	1.00	0.00	PROA	C
ATOM	162	HB1	LEU	9	52.528	42.569	35.444	1.00	0.00	PROA	H
ATOM	163	HB2	LEU	9	53.103	43.355	33.928	1.00	0.00	PROA	H
ATOM	164	CG	LEU	9	53.435	41.225	33.995	1.00	0.00	PROA	C
ATOM	165	HG	LEU	9	54.361	41.315	34.617	1.00	0.00	PROA	H
ATOM	166	CD1	LEU	9	52.741	39.933	34.371	1.00	0.00	PROA	C
ATOM	167	HD11	LEU	9	51.766	39.798	33.859	1.00	0.00	PROA	H

ATOM	168	HD12	LEU	9	52.568	40.028	35.440	1.00	0.00	PROA	H
ATOM	169	HD13	LEU	9	53.379	39.043	34.201	1.00	0.00	PROA	H
ATOM	170	CD2	LEU	9	53.893	41.097	32.548	1.00	0.00	PROA	C
ATOM	171	HD21	LEU	9	54.352	42.043	32.204	1.00	0.00	PROA	H
ATOM	172	HD22	LEU	9	53.037	40.829	31.898	1.00	0.00	PROA	H
ATOM	173	HD23	LEU	9	54.645	40.282	32.475	1.00	0.00	PROA	H
ATOM	174	C	LEU	9	50.480	43.720	34.395	1.00	0.00	PROA	C
ATOM	175	O	LEU	9	49.832	43.628	35.436	1.00	0.00	PROA	O
ATOM	176	N	LYS	10	50.575	44.899	33.725	1.00	0.00	PROA	N
ATOM	177	HN	LYS	10	51.067	45.023	32.872	1.00	0.00	PROA	H
ATOM	178	CA	LYS	10	49.655	45.943	33.966	1.00	0.00	PROA	C
ATOM	179	HA	LYS	10	49.641	46.042	35.047	1.00	0.00	PROA	H
ATOM	180	CB	LYS	10	50.023	47.369	33.408	1.00	0.00	PROA	C
ATOM	181	HB1	LYS	10	49.109	47.968	33.182	1.00	0.00	PROA	H
ATOM	182	HB2	LYS	10	50.550	47.194	32.451	1.00	0.00	PROA	H
ATOM	183	CG	LYS	10	50.962	48.224	34.308	1.00	0.00	PROA	C
ATOM	184	HG1	LYS	10	51.427	49.034	33.715	1.00	0.00	PROA	H
ATOM	185	HG2	LYS	10	51.743	47.548	34.731	1.00	0.00	PROA	H
ATOM	186	CD	LYS	10	50.236	48.865	35.478	1.00	0.00	PROA	C
ATOM	187	HD1	LYS	10	49.979	47.935	36.015	1.00	0.00	PROA	H
ATOM	188	HD2	LYS	10	49.328	49.415	35.124	1.00	0.00	PROA	H
ATOM	189	CE	LYS	10	51.013	49.644	36.525	1.00	0.00	PROA	C
ATOM	190	HE1	LYS	10	51.918	49.061	36.798	1.00	0.00	PROA	H
ATOM	191	HE2	LYS	10	50.397	49.820	37.441	1.00	0.00	PROA	H
ATOM	192	NZ	LYS	10	51.394	50.938	35.955	1.00	0.00	PROA	N
ATOM	193	HZ1	LYS	10	50.539	51.491	35.731	1.00	0.00	PROA	H
ATOM	194	HZ2	LYS	10	51.927	50.762	35.076	1.00	0.00	PROA	H
ATOM	195	HZ3	LYS	10	51.975	51.465	36.635	1.00	0.00	PROA	H
ATOM	196	C	LYS	10	48.306	45.355	33.628	1.00	0.00	PROA	C
ATOM	197	O	LYS	10	47.564	45.175	34.536	1.00	0.00	PROA	O
ATOM	198	N	ARG	11	47.899	44.813	32.490	1.00	0.00	PROA	N
ATOM	199	HN	ARG	11	48.537	44.769	31.720	1.00	0.00	PROA	H
ATOM	200	CA	ARG	11	46.483	44.347	32.408	1.00	0.00	PROA	C
ATOM	201	HA	ARG	11	45.772	45.086	32.554	1.00	0.00	PROA	H
ATOM	202	CB	ARG	11	46.189	43.858	31.022	1.00	0.00	PROA	C
ATOM	203	HB1	ARG	11	46.835	42.964	30.909	1.00	0.00	PROA	H
ATOM	204	HB2	ARG	11	46.527	44.637	30.298	1.00	0.00	PROA	H
ATOM	205	CG	ARG	11	44.713	43.516	30.795	1.00	0.00	PROA	C
ATOM	206	HG1	ARG	11	44.095	44.438	30.930	1.00	0.00	PROA	H
ATOM	207	HG2	ARG	11	44.382	42.777	31.558	1.00	0.00	PROA	H
ATOM	208	CD	ARG	11	44.525	42.957	29.394	1.00	0.00	PROA	C
ATOM	209	HD1	ARG	11	45.242	42.117	29.236	1.00	0.00	PROA	H
ATOM	210	HD2	ARG	11	44.722	43.751	28.639	1.00	0.00	PROA	H
ATOM	211	NE	ARG	11	43.130	42.428	29.303	1.00	0.00	PROA	N
ATOM	212	HE	ARG	11	42.914	41.653	29.891	1.00	0.00	PROA	H
ATOM	213	CZ	ARG	11	42.194	42.835	28.454	1.00	0.00	PROA	C
ATOM	214	NH1	ARG	11	42.349	43.810	27.587	1.00	0.00	PROA	N
ATOM	215	HH11	ARG	11	43.215	44.289	27.573	1.00	0.00	PROA	H
ATOM	216	HH12	ARG	11	41.589	44.138	27.027	1.00	0.00	PROA	H
ATOM	217	NH2	ARG	11	41.058	42.181	28.445	1.00	0.00	PROA	N
ATOM	218	HH21	ARG	11	40.903	41.432	29.084	1.00	0.00	PROA	H
ATOM	219	HH22	ARG	11	40.349	42.500	27.827	1.00	0.00	PROA	H
ATOM	220	C	ARG	11	45.886	43.508	33.493	1.00	0.00	PROA	C
ATOM	221	O	ARG	11	44.790	43.730	34.009	1.00	0.00	PROA	O
ATOM	222	N	LEU	12	46.710	42.634	33.972	1.00	0.00	PROA	N
ATOM	223	HN	LEU	12	47.628	42.568	33.578	1.00	0.00	PROA	H
ATOM	224	CA	LEU	12	46.400	41.890	35.097	1.00	0.00	PROA	C
ATOM	225	HA	LEU	12	45.519	41.318	34.900	1.00	0.00	PROA	H
ATOM	226	CB	LEU	12	47.583	41.064	35.284	1.00	0.00	PROA	C
ATOM	227	HB1	LEU	12	48.568	41.573	35.381	1.00	0.00	PROA	H
ATOM	228	HB2	LEU	12	47.663	40.337	34.473	1.00	0.00	PROA	H
ATOM	229	CG	LEU	12	47.332	40.215	36.408	1.00	0.00	PROA	C
ATOM	230	HG	LEU	12	47.259	40.686	37.424	1.00	0.00	PROA	H

ATOM	231	CD1	LEU	12	46.123	39.370	36.167	1.00	0.00	PROA	C
ATOM	232	HD11	LEU	12	46.348	38.785	35.246	1.00	0.00	PROA	H
ATOM	233	HD12	LEU	12	45.131	39.796	36.071	1.00	0.00	PROA	H
ATOM	234	HD13	LEU	12	45.918	38.755	37.047	1.00	0.00	PROA	H
ATOM	235	CD2	LEU	12	48.595	39.509	36.254	1.00	0.00	PROA	C
ATOM	236	HD21	LEU	12	49.513	40.101	36.357	1.00	0.00	PROA	H
ATOM	237	HD22	LEU	12	48.710	38.735	35.493	1.00	0.00	PROA	H
ATOM	238	HD23	LEU	12	48.345	39.156	37.213	1.00	0.00	PROA	H
ATOM	239	C	LEU	12	46.181	42.602	36.368	1.00	0.00	PROA	C
ATOM	240	O	LEU	12	45.220	42.354	37.087	1.00	0.00	PROA	O
ATOM	241	N	LEU	13	47.076	43.510	36.673	1.00	0.00	PROA	N
ATOM	242	HN	LEU	13	47.876	43.729	36.110	1.00	0.00	PROA	H
ATOM	243	CA	LEU	13	46.964	44.299	37.796	1.00	0.00	PROA	C
ATOM	244	HA	LEU	13	46.907	43.729	38.710	1.00	0.00	PROA	H
ATOM	245	CB	LEU	13	48.299	44.960	37.806	1.00	0.00	PROA	C
ATOM	246	HB1	LEU	13	48.508	45.516	36.892	1.00	0.00	PROA	H
ATOM	247	HB2	LEU	13	49.180	44.326	37.847	1.00	0.00	PROA	H
ATOM	248	CG	LEU	13	48.669	45.667	38.977	1.00	0.00	PROA	C
ATOM	249	HG	LEU	13	49.680	46.076	38.681	1.00	0.00	PROA	H
ATOM	250	CD1	LEU	13	48.070	46.685	38.682	1.00	0.00	PROA	C
ATOM	251	HD11	LEU	13	46.982	46.676	39.008	1.00	0.00	PROA	H
ATOM	252	HD12	LEU	13	48.824	47.591	38.613	1.00	0.00	PROA	H
ATOM	253	HD13	LEU	13	48.466	47.735	38.714	1.00	0.00	PROA	H
ATOM	254	CD2	LEU	13	48.120	45.227	40.349	1.00	0.00	PROA	C
ATOM	255	HD21	LEU	13	48.761	44.385	40.587	1.00	0.00	PROA	H
ATOM	256	HD22	LEU	13	47.016	45.134	40.477	1.00	0.00	PROA	H
ATOM	257	HD23	LEU	13	48.263	45.888	41.193	1.00	0.00	PROA	H
ATOM	258	C	LEU	13	45.685	45.074	37.805	1.00	0.00	PROA	C
ATOM	259	O	LEU	13	45.198	45.053	38.872	1.00	0.00	PROA	O
ATOM	260	N	ILE	14	45.099	45.701	36.775	1.00	0.00	PROA	N
ATOM	261	HN	ILE	14	45.653	45.767	35.957	1.00	0.00	PROA	H
ATOM	262	CA	ILE	14	43.913	46.529	36.783	1.00	0.00	PROA	C
ATOM	263	HA	ILE	14	43.870	47.114	37.686	1.00	0.00	PROA	H
ATOM	264	CB	ILE	14	43.790	47.503	35.596	1.00	0.00	PROA	C
ATOM	265	HB	ILE	14	43.042	48.137	35.881	1.00	0.00	PROA	H
ATOM	266	CG2	ILE	14	44.245	49.087	35.812	1.00	0.00	PROA	C
ATOM	267	HG21	ILE	14	45.290	49.336	35.872	1.00	0.00	PROA	H
ATOM	268	HG22	ILE	14	44.566	49.270	36.856	1.00	0.00	PROA	H
ATOM	269	HG23	ILE	14	44.477	49.635	34.869	1.00	0.00	PROA	H
ATOM	270	CG1	ILE	14	43.368	46.760	34.186	1.00	0.00	PROA	C
ATOM	271	HG11	ILE	14	42.480	46.114	34.258	1.00	0.00	PROA	H
ATOM	272	HG12	ILE	14	43.962	45.968	33.707	1.00	0.00	PROA	H
ATOM	273	CD	ILE	14	43.293	47.817	33.078	1.00	0.00	PROA	C
ATOM	274	HD1	ILE	14	44.071	48.507	33.164	1.00	0.00	PROA	H
ATOM	275	HD2	ILE	14	42.668	48.707	33.193	1.00	0.00	PROA	H
ATOM	276	HD3	ILE	14	43.219	47.376	32.061	1.00	0.00	PROA	H
ATOM	277	C	ILE	14	42.773	45.581	36.852	1.00	0.00	PROA	C
ATOM	278	O	ILE	14	41.845	45.790	37.612	1.00	0.00	PROA	O
ATOM	279	N	ILE	15	42.833	44.428	36.170	1.00	0.00	PROA	N
ATOM	280	HN	ILE	15	43.632	44.185	35.621	1.00	0.00	PROA	H
ATOM	281	CA	ILE	15	41.829	43.416	36.302	1.00	0.00	PROA	C
ATOM	282	HA	ILE	15	40.856	43.859	36.113	1.00	0.00	PROA	H
ATOM	283	CB	ILE	15	42.090	42.296	35.337	1.00	0.00	PROA	C
ATOM	284	HB	ILE	15	43.150	41.979	35.424	1.00	0.00	PROA	H
ATOM	285	CG2	ILE	15	41.247	41.111	35.661	1.00	0.00	PROA	C
ATOM	286	HG21	ILE	15	40.247	41.455	35.750	1.00	0.00	PROA	H
ATOM	287	HG22	ILE	15	41.557	40.527	36.566	1.00	0.00	PROA	H
ATOM	288	HG23	ILE	15	41.046	40.507	34.769	1.00	0.00	PROA	H
ATOM	289	CG1	ILE	15	41.809	42.699	33.876	1.00	0.00	PROA	C
ATOM	290	HG11	ILE	15	42.190	41.869	33.241	1.00	0.00	PROA	H
ATOM	291	HG12	ILE	15	42.390	43.613	33.657	1.00	0.00	PROA	H
ATOM	292	CD	ILE	15	40.345	42.946	33.494	1.00	0.00	PROA	C
ATOM	293	HD1	ILE	15	39.900	43.764	34.098	1.00	0.00	PROA	H

ATOM	294	HD2	ILE	15	39.735	42.033	33.611	1.00	0.00	PROA	H
ATOM	295	HD3	ILE	15	40.278	43.213	32.425	1.00	0.00	PROA	H
ATOM	296	C	ILE	15	41.769	42.979	37.727	1.00	0.00	PROA	C
ATOM	297	O	ILE	15	40.734	43.047	38.301	1.00	0.00	PROA	O
ATOM	298	N	VAL	16	42.846	42.759	38.450	1.00	0.00	PROA	N
ATOM	299	HN	VAL	16	43.751	42.786	38.021	1.00	0.00	PROA	H
ATOM	300	CA	VAL	16	42.821	42.594	39.881	1.00	0.00	PROA	C
ATOM	301	HA	VAL	16	42.113	41.884	40.117	1.00	0.00	PROA	H
ATOM	302	CB	VAL	16	44.271	42.281	40.215	1.00	0.00	PROA	C
ATOM	303	HB	VAL	16	44.939	43.068	39.831	1.00	0.00	PROA	H
ATOM	304	CG1	VAL	16	44.390	42.418	41.622	1.00	0.00	PROA	C
ATOM	305	HG11	VAL	16	43.656	41.793	42.183	1.00	0.00	PROA	H
ATOM	306	HG12	VAL	16	44.360	43.469	41.969	1.00	0.00	PROA	H
ATOM	307	HG13	VAL	16	45.412	42.062	41.765	1.00	0.00	PROA	H
ATOM	308	CG2	VAL	16	44.964	40.955	39.758	1.00	0.00	PROA	C
ATOM	309	HG21	VAL	16	44.753	40.745	38.702	1.00	0.00	PROA	H
ATOM	310	HG22	VAL	16	44.711	40.004	40.187	1.00	0.00	PROA	H
ATOM	311	HG23	VAL	16	45.966	40.805	40.197	1.00	0.00	PROA	H
ATOM	312	C	VAL	16	41.929	43.498	40.757	1.00	0.00	PROA	C
ATOM	313	O	VAL	16	41.460	43.178	41.837	1.00	0.00	PROA	O
ATOM	314	N	VAL	17	41.609	44.618	40.095	1.00	0.00	PROA	N
ATOM	315	HN	VAL	17	42.414	44.585	39.520	1.00	0.00	PROA	H
ATOM	316	CA	VAL	17	41.259	46.068	40.427	1.00	0.00	PROA	C
ATOM	317	HA	VAL	17	41.008	45.606	41.403	1.00	0.00	PROA	H
ATOM	318	CB	VAL	17	42.161	47.788	40.156	1.00	0.00	PROA	C
ATOM	319	HB	VAL	17	42.190	47.798	39.071	1.00	0.00	PROA	H
ATOM	320	CG1	VAL	17	41.058	48.829	40.515	1.00	0.00	PROA	C
ATOM	321	HG11	VAL	17	40.153	48.982	41.173	1.00	0.00	PROA	H
ATOM	322	HG12	VAL	17	40.267	48.392	39.913	1.00	0.00	PROA	H
ATOM	323	HG13	VAL	17	41.395	49.880	40.538	1.00	0.00	PROA	H
ATOM	324	CG2	VAL	17	43.861	48.388	40.623	1.00	0.00	PROA	C
ATOM	325	HG21	VAL	17	44.685	47.659	40.937	1.00	0.00	PROA	H
ATOM	326	HG22	VAL	17	44.299	48.126	41.678	1.00	0.00	PROA	H
ATOM	327	HG23	VAL	17	44.266	49.351	40.538	1.00	0.00	PROA	H
ATOM	328	C	VAL	17	40.040	46.244	40.404	1.00	0.00	PROA	C
ATOM	329	O	VAL	17	39.305	46.694	41.263	1.00	0.00	PROA	O
ATOM	330	N	VAL	18	39.694	45.703	39.386	1.00	0.00	PROA	N
ATOM	331	HN	VAL	18	40.386	45.347	38.743	1.00	0.00	PROA	H
ATOM	332	CA	VAL	18	38.414	45.550	39.283	1.00	0.00	PROA	C
ATOM	333	HA	VAL	18	37.976	46.516	39.441	1.00	0.00	PROA	H
ATOM	334	CB	VAL	18	38.413	45.207	37.844	1.00	0.00	PROA	C
ATOM	335	HB	VAL	18	39.205	44.570	37.411	1.00	0.00	PROA	H
ATOM	336	CG1	VAL	18	37.314	44.425	38.141	1.00	0.00	PROA	C
ATOM	337	HG11	VAL	18	36.414	44.699	38.388	1.00	0.00	PROA	H
ATOM	338	HG12	VAL	18	36.547	43.736	37.798	1.00	0.00	PROA	H
ATOM	339	HG13	VAL	18	37.351	43.657	37.434	1.00	0.00	PROA	H
ATOM	340	CG2	VAL	18	38.576	46.490	37.127	1.00	0.00	PROA	C
ATOM	341	HG21	VAL	18	39.613	46.644	37.346	1.00	0.00	PROA	H
ATOM	342	HG22	VAL	18	37.999	47.403	37.374	1.00	0.00	PROA	H
ATOM	343	HG23	VAL	18	38.563	46.691	36.075	1.00	0.00	PROA	H
ATOM	344	C	VAL	18	37.891	44.614	40.433	1.00	0.00	PROA	C
ATOM	345	O	VAL	18	36.765	44.779	40.867	1.00	0.00	PROA	O
ATOM	346	N	ILE	19	38.669	43.510	40.769	1.00	0.00	PROA	N
ATOM	347	HN	ILE	19	39.285	43.284	40.018	1.00	0.00	PROA	H
ATOM	348	CA	ILE	19	37.927	42.329	41.434	1.00	0.00	PROA	C
ATOM	349	HA	ILE	19	36.903	42.642	41.362	1.00	0.00	PROA	H
ATOM	350	CB	ILE	19	38.101	40.679	41.125	1.00	0.00	PROA	C
ATOM	351	HB	ILE	19	37.650	40.074	41.930	1.00	0.00	PROA	H
ATOM	352	CG2	ILE	19	37.331	39.732	39.949	1.00	0.00	PROA	C
ATOM	353	HG21	ILE	19	37.446	39.807	38.856	1.00	0.00	PROA	H
ATOM	354	HG22	ILE	19	36.309	39.967	39.571	1.00	0.00	PROA	H
ATOM	355	HG23	ILE	19	38.027	39.134	39.318	1.00	0.00	PROA	H
ATOM	356	CG1	ILE	19	39.599	40.481	41.270	1.00	0.00	PROA	C

ATOM	357	HG11	ILE	19	39.957	40.769	42.197	1.00	0.00	PROA H
ATOM	358	HG12	ILE	19	40.374	41.097	40.785	1.00	0.00	PROA H
ATOM	359	CD	ILE	19	39.818	39.062	41.064	1.00	0.00	PROA C
ATOM	360	HD1	ILE	19	39.840	38.811	39.970	1.00	0.00	PROA H
ATOM	361	HD2	ILE	19	39.279	38.412	41.744	1.00	0.00	PROA H
ATOM	362	HD3	ILE	19	40.727	38.733	41.516	1.00	0.00	PROA H
ATOM	363	C	ILE	19	37.732	42.654	42.680	1.00	0.00	PROA C
ATOM	364	O	ILE	19	36.779	42.243	43.319	1.00	0.00	PROA O
ATOM	365	N	GLU	20	38.664	43.540	43.019	1.00	0.00	PROA N
ATOM	366	HN	GLU	20	39.400	43.703	42.365	1.00	0.00	PROA H
ATOM	367	CA	GLU	20	38.864	43.979	44.283	1.00	0.00	PROA C
ATOM	368	HA	GLU	20	38.459	43.195	44.853	1.00	0.00	PROA H
ATOM	369	CB	GLU	20	40.462	44.209	44.584	1.00	0.00	PROA C
ATOM	370	HB1	GLU	20	40.820	44.542	43.600	1.00	0.00	PROA H
ATOM	371	HB2	GLU	20	41.183	43.415	44.599	1.00	0.00	PROA H
ATOM	372	CG	GLU	20	41.071	44.949	45.805	1.00	0.00	PROA C
ATOM	373	HG1	GLU	20	40.631	45.839	45.905	1.00	0.00	PROA H
ATOM	374	HG2	GLU	20	42.060	45.340	45.622	1.00	0.00	PROA H
ATOM	375	CD	GLU	20	41.013	44.334	47.141	1.00	0.00	PROA C
ATOM	376	OE1	GLU	20	41.151	44.990	48.205	1.00	0.00	PROA O
ATOM	377	OE2	GLU	20	40.619	43.161	47.093	1.00	0.00	PROA O
ATOM	378	C	GLU	20	37.689	44.820	44.389	1.00	0.00	PROA C
ATOM	379	O	GLU	20	36.941	44.710	45.325	1.00	0.00	PROA O
ATOM	380	N	LEU	21	37.308	45.664	43.387	1.00	0.00	PROA N
ATOM	381	HN	LEU	21	37.898	45.769	42.581	1.00	0.00	PROA H
ATOM	382	CA	LEU	21	36.281	46.632	43.533	1.00	0.00	PROA C
ATOM	383	HA	LEU	21	36.314	47.178	44.445	1.00	0.00	PROA H
ATOM	384	CB	LEU	21	36.373	47.664	42.449	1.00	0.00	PROA C
ATOM	385	HB1	LEU	21	36.349	46.868	41.775	1.00	0.00	PROA H
ATOM	386	HB2	LEU	21	37.420	47.939	42.393	1.00	0.00	PROA H
ATOM	387	CG	LEU	21	35.460	48.889	42.150	1.00	0.00	PROA C
ATOM	388	HG	LEU	21	35.634	49.299	41.158	1.00	0.00	PROA H
ATOM	389	CD1	LEU	21	34.022	48.748	41.792	1.00	0.00	PROA C
ATOM	390	HD11	LEU	21	33.377	48.385	42.468	1.00	0.00	PROA H
ATOM	391	HD12	LEU	21	33.710	48.103	40.963	1.00	0.00	PROA H
ATOM	392	HD13	LEU	21	33.595	49.745	41.661	1.00	0.00	PROA H
ATOM	393	CD2	LEU	21	35.730	50.078	43.052	1.00	0.00	PROA C
ATOM	394	HD21	LEU	21	36.657	50.098	43.654	1.00	0.00	PROA H
ATOM	395	HD22	LEU	21	35.302	49.582	43.768	1.00	0.00	PROA H
ATOM	396	HD23	LEU	21	34.968	50.879	43.143	1.00	0.00	PROA H
ATOM	397	C	LEU	21	35.256	45.620	43.602	1.00	0.00	PROA C
ATOM	398	O	LEU	21	34.801	45.511	44.690	1.00	0.00	PROA O
ATOM	399	N	VAL	22	34.964	44.723	42.709	1.00	0.00	PROA N
ATOM	400	HN	VAL	22	35.520	44.664	41.881	1.00	0.00	PROA H
ATOM	401	CA	VAL	22	33.834	43.889	42.895	1.00	0.00	PROA C
ATOM	402	HA	VAL	22	33.222	44.733	42.815	1.00	0.00	PROA H
ATOM	403	CB	VAL	22	33.689	42.906	41.751	1.00	0.00	PROA C
ATOM	404	HB	VAL	22	34.694	42.440	41.649	1.00	0.00	PROA H
ATOM	405	CG1	VAL	22	32.663	41.791	41.984	1.00	0.00	PROA C
ATOM	406	HG11	VAL	22	31.625	42.140	42.137	1.00	0.00	PROA H
ATOM	407	HG12	VAL	22	32.936	41.132	42.823	1.00	0.00	PROA H
ATOM	408	HG13	VAL	22	32.609	41.208	41.062	1.00	0.00	PROA H
ATOM	409	CG2	VAL	22	33.315	43.611	40.439	1.00	0.00	PROA C
ATOM	410	HG21	VAL	22	34.022	44.416	40.291	1.00	0.00	PROA H
ATOM	411	HG22	VAL	22	32.312	44.080	40.450	1.00	0.00	PROA H
ATOM	412	HG23	VAL	22	33.368	42.898	39.590	1.00	0.00	PROA H
ATOM	413	C	VAL	22	33.559	43.204	44.256	1.00	0.00	PROA C
ATOM	414	O	VAL	22	32.488	43.123	44.853	1.00	0.00	PROA O
ATOM	415	N	VAL	23	34.612	42.770	44.874	1.00	0.00	PROA N
ATOM	416	HN	VAL	23	35.499	42.830	44.402	1.00	0.00	PROA H
ATOM	417	CA	VAL	23	34.570	42.280	46.208	1.00	0.00	PROA C
ATOM	418	HA	VAL	23	33.801	41.542	46.300	1.00	0.00	PROA H
ATOM	419	CB	VAL	23	35.923	41.568	46.313	1.00	0.00	PROA C

ATOM	420	HB	VAL	23	36.722	42.186	45.842	1.00	0.00	PROA	H
ATOM	421	CG1	VAL	23	36.201	40.870	47.629	1.00	0.00	PROA	C
ATOM	422	HG11	VAL	23	35.352	40.942	48.320	1.00	0.00	PROA	H
ATOM	423	HG12	VAL	23	36.627	41.428	48.349	1.00	0.00	PROA	H
ATOM	424	HG13	VAL	23	37.214	40.497	47.837	1.00	0.00	PROA	H
ATOM	425	CG2	VAL	23	36.054	40.348	45.481	1.00	0.00	PROA	C
ATOM	426	HG21	VAL	23	35.890	40.453	44.417	1.00	0.00	PROA	H
ATOM	427	HG22	VAL	23	35.272	39.732	45.821	1.00	0.00	PROA	H
ATOM	428	HG23	VAL	23	37.015	39.858	45.740	1.00	0.00	PROA	H
ATOM	429	C	VAL	23	34.076	43.364	47.217	1.00	0.00	PROA	C
ATOM	430	O	VAL	23	33.494	43.104	48.252	1.00	0.00	PROA	O
ATOM	431	N	LYS	24	34.316	44.659	46.975	1.00	0.00	PROA	N
ATOM	432	HN	LYS	24	34.922	44.912	46.191	1.00	0.00	PROA	H
ATOM	433	CA	LYS	24	34.143	45.696	47.897	1.00	0.00	PROA	C
ATOM	434	HA	LYS	24	34.387	45.404	48.896	1.00	0.00	PROA	H
ATOM	435	CB	LYS	24	35.129	46.762	47.504	1.00	0.00	PROA	C
ATOM	436	HB1	LYS	24	34.698	47.644	47.099	1.00	0.00	PROA	H
ATOM	437	HB2	LYS	24	35.876	46.631	46.724	1.00	0.00	PROA	H
ATOM	438	CG	LYS	24	35.416	47.474	48.659	1.00	0.00	PROA	C
ATOM	439	HG1	LYS	24	34.419	47.868	49.029	1.00	0.00	PROA	H
ATOM	440	HG2	LYS	24	35.515	48.514	48.350	1.00	0.00	PROA	H
ATOM	441	CD	LYS	24	36.382	46.693	49.671	1.00	0.00	PROA	C
ATOM	442	HD1	LYS	24	35.592	46.353	50.397	1.00	0.00	PROA	H
ATOM	443	HD2	LYS	24	37.122	47.338	50.281	1.00	0.00	PROA	H
ATOM	444	CE	LYS	24	37.893	46.643	49.801	1.00	0.00	PROA	C
ATOM	445	HE1	LYS	24	38.692	46.245	50.472	1.00	0.00	PROA	H
ATOM	446	HE2	LYS	24	38.398	47.230	49.036	1.00	0.00	PROA	H
ATOM	447	NZ	LYS	24	37.409	45.562	49.412	1.00	0.00	PROA	N
ATOM	448	HZ1	LYS	24	36.844	45.669	48.496	1.00	0.00	PROA	H
ATOM	449	HZ2	LYS	24	37.094	44.747	49.902	1.00	0.00	PROA	H
ATOM	450	HZ3	LYS	24	38.175	45.114	48.877	1.00	0.00	PROA	H
ATOM	451	C	LYS	24	32.691	46.057	47.891	1.00	0.00	PROA	C
ATOM	452	O	LYS	24	32.134	46.513	48.877	1.00	0.00	PROA	O
ATOM	453	N	VAL	25	32.023	45.783	46.766	1.00	0.00	PROA	N
ATOM	454	HN	VAL	25	32.584	45.689	45.934	1.00	0.00	PROA	H
ATOM	455	CA	VAL	25	30.614	45.996	46.667	1.00	0.00	PROA	C
ATOM	456	HA	VAL	25	30.347	46.878	47.202	1.00	0.00	PROA	H
ATOM	457	CB	VAL	25	30.081	46.358	45.362	1.00	0.00	PROA	C
ATOM	458	HB	VAL	25	30.687	45.797	44.736	1.00	0.00	PROA	H
ATOM	459	CG1	VAL	25	28.687	45.899	44.921	1.00	0.00	PROA	C
ATOM	460	HG11	VAL	25	28.073	46.641	45.454	1.00	0.00	PROA	H
ATOM	461	HG12	VAL	25	28.437	44.848	45.110	1.00	0.00	PROA	H
ATOM	462	HG13	VAL	25	28.476	45.967	43.825	1.00	0.00	PROA	H
ATOM	463	CG2	VAL	25	30.347	47.879	45.280	1.00	0.00	PROA	C
ATOM	464	HG21	VAL	25	31.240	48.382	45.032	1.00	0.00	PROA	H
ATOM	465	HG22	VAL	25	29.653	48.598	44.902	1.00	0.00	PROA	H
ATOM	466	HG23	VAL	25	30.823	47.654	44.387	1.00	0.00	PROA	H
ATOM	467	C	VAL	25	29.936	44.848	47.222	1.00	0.00	PROA	C
ATOM	468	O	VAL	25	28.922	45.047	47.872	1.00	0.00	PROA	O
ATOM	469	N	ILE	26	30.524	43.641	47.077	1.00	0.00	PROA	N
ATOM	470	HN	ILE	26	31.352	43.521	46.516	1.00	0.00	PROA	H
ATOM	471	CA	ILE	26	30.048	42.477	47.789	1.00	0.00	PROA	C
ATOM	472	HA	ILE	26	28.992	42.398	47.586	1.00	0.00	PROA	H
ATOM	473	CB	ILE	26	30.745	41.209	47.293	1.00	0.00	PROA	C
ATOM	474	HB	ILE	26	31.845	41.375	47.312	1.00	0.00	PROA	H
ATOM	475	CG2	ILE	26	30.424	40.000	48.193	1.00	0.00	PROA	C
ATOM	476	HG21	ILE	26	29.330	39.806	48.193	1.00	0.00	PROA	H
ATOM	477	HG22	ILE	26	30.763	40.159	49.237	1.00	0.00	PROA	H
ATOM	478	HG23	ILE	26	30.937	39.094	47.810	1.00	0.00	PROA	H
ATOM	479	CG1	ILE	26	30.339	40.904	45.830	1.00	0.00	PROA	C
ATOM	480	HG11	ILE	26	30.343	41.841	45.233	1.00	0.00	PROA	H
ATOM	481	HG12	ILE	26	29.296	40.515	45.830	1.00	0.00	PROA	H
ATOM	482	CD	ILE	26	31.275	39.900	45.148	1.00	0.00	PROA	C

ATOM	483	HD1	ILE	26	31.257	38.920	45.668	1.00	0.00	PROA H
ATOM	484	HD2	ILE	26	32.315	40.284	45.113	1.00	0.00	PROA H
ATOM	485	HD3	ILE	26	30.985	39.729	44.096	1.00	0.00	PROA H
ATOM	486	C	ILE	26	30.134	42.651	49.298	1.00	0.00	PROA C
ATOM	487	O	ILE	26	29.196	42.360	50.032	1.00	0.00	PROA O
ATOM	488	N	VAL	27	31.236	43.191	49.818	1.00	0.00	PROA N
ATOM	489	HN	VAL	27	32.045	43.360	49.241	1.00	0.00	PROA H
ATOM	490	CA	VAL	27	31.350	43.506	51.224	1.00	0.00	PROA C
ATOM	491	HA	VAL	27	31.069	42.634	51.799	1.00	0.00	PROA H
ATOM	492	CB	VAL	27	32.744	43.746	51.555	1.00	0.00	PROA C
ATOM	493	HB	VAL	27	33.272	44.373	50.785	1.00	0.00	PROA H
ATOM	494	CG1	VAL	27	32.933	44.412	52.947	1.00	0.00	PROA C
ATOM	495	HG11	VAL	27	32.567	43.764	53.767	1.00	0.00	PROA H
ATOM	496	HG12	VAL	27	32.623	45.470	52.980	1.00	0.00	PROA H
ATOM	497	HG13	VAL	27	33.941	44.622	53.232	1.00	0.00	PROA H
ATOM	498	CG2	VAL	27	33.176	42.279	51.492	1.00	0.00	PROA C
ATOM	499	HG21	VAL	27	33.294	41.803	50.492	1.00	0.00	PROA H
ATOM	500	HG22	VAL	27	33.095	41.589	52.339	1.00	0.00	PROA H
ATOM	501	HG23	VAL	27	34.131	42.504	51.748	1.00	0.00	PROA H
ATOM	502	C	VAL	27	30.505	44.614	51.737	1.00	0.00	PROA C
ATOM	503	O	VAL	27	30.001	44.565	52.858	1.00	0.00	PROA O
ATOM	504	N	GLY	28	30.321	45.655	50.914	1.00	0.00	PROA N
ATOM	505	HN	GLY	28	30.805	45.689	50.035	1.00	0.00	PROA H
ATOM	506	CA	GLY	28	29.415	46.740	51.234	1.00	0.00	PROA C
ATOM	507	HA1	GLY	28	29.515	47.480	50.459	1.00	0.00	PROA H
ATOM	508	HA2	GLY	28	29.672	47.117	52.215	1.00	0.00	PROA H
ATOM	509	C	GLY	28	27.975	46.287	51.267	1.00	0.00	PROA C
ATOM	510	O	GLY	28	27.209	46.649	52.153	1.00	0.00	PROA O
ATOM	511	N	ALA	29	27.571	45.427	50.313	1.00	0.00	PROA N
ATOM	512	HN	ALA	29	28.184	45.187	49.553	1.00	0.00	PROA H
ATOM	513	CA	ALA	29	26.278	44.771	50.316	1.00	0.00	PROA C
ATOM	514	HA	ALA	29	25.513	45.534	50.368	1.00	0.00	PROA H
ATOM	515	CB	ALA	29	26.113	43.968	49.010	1.00	0.00	PROA C
ATOM	516	HB1	ALA	29	26.910	43.200	48.914	1.00	0.00	PROA H
ATOM	517	HB2	ALA	29	26.192	44.653	48.137	1.00	0.00	PROA H
ATOM	518	HB3	ALA	29	25.122	43.470	48.971	1.00	0.00	PROA H
ATOM	519	C	ALA	29	26.064	43.883	51.538	1.00	0.00	PROA C
ATOM	520	O	ALA	29	25.015	43.917	52.178	1.00	0.00	PROA O
ATOM	521	N	LEU	30	27.089	43.105	51.936	1.00	0.00	PROA N
ATOM	522	HN	LEU	30	27.920	43.036	51.378	1.00	0.00	PROA H
ATOM	523	CA	LEU	30	27.083	42.321	53.157	1.00	0.00	PROA C
ATOM	524	HA	LEU	30	26.198	41.697	53.143	1.00	0.00	PROA H
ATOM	525	CB	LEU	30	28.343	41.430	53.188	1.00	0.00	PROA C
ATOM	526	HB1	LEU	30	29.244	42.081	53.220	1.00	0.00	PROA H
ATOM	527	HB2	LEU	30	28.380	40.852	52.237	1.00	0.00	PROA H
ATOM	528	CG	LEU	30	28.418	40.430	54.355	1.00	0.00	PROA C
ATOM	529	HG	LEU	30	28.371	40.986	55.320	1.00	0.00	PROA H
ATOM	530	CD1	LEU	30	27.273	39.410	54.330	1.00	0.00	PROA C
ATOM	531	HD11	LEU	30	27.300	38.819	53.390	1.00	0.00	PROA H
ATOM	532	HD12	LEU	30	26.284	39.914	54.393	1.00	0.00	PROA H
ATOM	533	HD13	LEU	30	27.361	38.710	55.189	1.00	0.00	PROA H
ATOM	534	CD2	LEU	30	29.746	39.680	54.294	1.00	0.00	PROA C
ATOM	535	HD21	LEU	30	30.601	40.385	54.356	1.00	0.00	PROA H
ATOM	536	HD22	LEU	30	29.816	39.123	53.334	1.00	0.00	PROA H
ATOM	537	HD23	LEU	30	29.811	38.957	55.133	1.00	0.00	PROA H
ATOM	538	C	LEU	30	26.955	43.170	54.417	1.00	0.00	PROA C
ATOM	539	O	LEU	30	26.261	42.805	55.358	1.00	0.00	PROA O
ATOM	540	N	LEU	31	27.580	44.363	54.444	1.00	0.00	PROA N
ATOM	541	HN	LEU	31	28.219	44.603	53.710	1.00	0.00	PROA H
ATOM	542	CA	LEU	31	27.410	45.365	55.488	1.00	0.00	PROA C
ATOM	543	HA	LEU	31	27.532	44.877	56.445	1.00	0.00	PROA H
ATOM	544	CB	LEU	31	28.510	46.440	55.315	1.00	0.00	PROA C
ATOM	545	HB1	LEU	31	28.409	46.889	54.305	1.00	0.00	PROA H

```
ATOM    546  HB2 LEU    31      29.497  45.926  55.355  1.00  0.00      PROA H
ATOM    547  CG  LEU    31      28.532  47.590  56.339  1.00  0.00      PROA C
ATOM    548  HG  LEU    31      27.553  48.124  56.307  1.00  0.00      PROA H
ATOM    549  CD1 LEU    31      28.769  47.092  57.772  1.00  0.00      PROA C
ATOM    550 HD11 LEU    31      29.752  46.580  57.834  1.00  0.00      PROA H
ATOM    551 HD12 LEU    31      27.969  46.382  58.071  1.00  0.00      PROA H
ATOM    552 HD13 LEU    31      28.771  47.953  58.473  1.00  0.00      PROA H
ATOM    553  CD2 LEU    31      29.637  48.585  55.961  1.00  0.00      PROA C
ATOM    554 HD21 LEU    31      29.463  48.991  54.943  1.00  0.00      PROA H
ATOM    555 HD22 LEU    31      30.620  48.066  55.971  1.00  0.00      PROA H
ATOM    556 HD23 LEU    31      29.669  49.425  56.686  1.00  0.00      PROA H
ATOM    557  C   LEU    31      26.009  45.978  55.536  1.00  0.00      PROA C
ATOM    558  O   LEU    31      25.528  46.378  56.592  1.00  0.00      PROA O
ATOM    559  N   MET    32      25.271  45.990  54.409  1.00  0.00      PROA N
ATOM    560  HN  MET    32      25.700  45.815  53.523  1.00  0.00      PROA H
ATOM    561  CA  MET    32      23.840  46.246  54.401  1.00  0.00      PROA C
ATOM    562  HA  MET    32      23.627  47.044  55.103  1.00  0.00      PROA H
ATOM    563  CB  MET    32      23.373  46.679  53.010  1.00  0.00      PROA C
ATOM    564  HB1 MET    32      22.266  46.854  53.011  1.00  0.00      PROA H
ATOM    565  HB2 MET    32      23.605  45.912  52.244  1.00  0.00      PROA H
ATOM    566  CG  MET    32      24.024  47.968  52.577  1.00  0.00      PROA C
ATOM    567  HG1 MET    32      25.133  47.908  52.517  1.00  0.00      PROA H
ATOM    568  HG2 MET    32      23.772  48.661  53.401  1.00  0.00      PROA H
ATOM    569  SD  MET    32      23.259  48.402  51.010  1.00  0.00      PROA S
ATOM    570  CE  MET    32      24.219  49.841  50.625  1.00  0.00      PROA C
ATOM    571  HE1 MET    32      25.149  49.297  50.591  1.00  0.00      PROA H
ATOM    572  HE2 MET    32      24.571  50.684  51.242  1.00  0.00      PROA H
ATOM    573  HE3 MET    32      23.871  50.387  49.733  1.00  0.00      PROA H
ATOM    574  C   MET    32      22.987  45.063  54.833  1.00  0.00      PROA C
ATOM    575  O   MET    32      21.779  45.194  55.008  1.00  0.00      PROA O
ATOM    576  N   GLY    33      23.590  43.876  55.028  1.00  0.00      PROA N
ATOM    577  HN  GLY    33      24.548  43.757  54.765  1.00  0.00      PROA H
ATOM    578  CA  GLY    33      23.006  42.792  55.811  1.00  0.00      PROA C
ATOM    579  HA1 GLY    33      22.268  43.195  56.490  1.00  0.00      PROA H
ATOM    580  HA2 GLY    33      23.812  42.310  56.345  1.00  0.00      PROA H
ATOM    581  C   GLY    33      22.306  41.722  55.028  1.00  0.00      PROA C
ATOM    582  O   GLY    33      21.654  40.880  55.638  1.00  0.00      PROA O
ATOM    583  N   LEU    34      22.406  41.743  53.687  1.00  0.00      PROA N
ATOM    584  HN  LEU    34      22.943  42.436  53.232  1.00  0.00      PROA H
ATOM    585  CA  LEU    34      21.799  40.760  52.808  1.00  0.00      PROA C
ATOM    586  HA  LEU    34      20.741  40.723  53.023  1.00  0.00      PROA H
ATOM    587  CB  LEU    34      22.000  41.174  51.311  1.00  0.00      PROA C
ATOM    588  HB1 LEU    34      21.749  40.300  50.670  1.00  0.00      PROA H
ATOM    589  HB2 LEU    34      23.079  41.392  51.168  1.00  0.00      PROA H
ATOM    590  CG  LEU    34      21.179  42.384  50.778  1.00  0.00      PROA C
ATOM    591  HG  LEU    34      21.416  42.479  49.689  1.00  0.00      PROA H
ATOM    592  CD1 LEU    34      19.666  42.158  50.910  1.00  0.00      PROA C
ATOM    593 HD11 LEU    34      19.376  42.186  51.983  1.00  0.00      PROA H
ATOM    594 HD12 LEU    34      19.381  41.171  50.490  1.00  0.00      PROA H
ATOM    595 HD13 LEU    34      19.117  42.963  50.381  1.00  0.00      PROA H
ATOM    596  CD2 LEU    34      21.545  43.733  51.411  1.00  0.00      PROA C
ATOM    597 HD21 LEU    34      22.642  43.887  51.376  1.00  0.00      PROA H
ATOM    598 HD22 LEU    34      21.212  43.757  52.471  1.00  0.00      PROA H
ATOM    599 HD23 LEU    34      21.041  44.559  50.864  1.00  0.00      PROA H
ATOM    600  C   LEU    34      22.320  39.307  53.060  1.00  0.00      PROA C
ATOM    601  OT1 LEU    34      23.206  39.079  53.929  1.00  0.00      PROA O
ATOM    602  OT2 LEU    34      21.802  38.389  52.366  1.00  0.00      PROA O
TER
```

Quality of AlphaFold Best Predicted Model

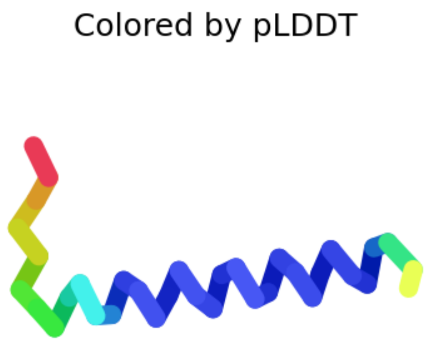

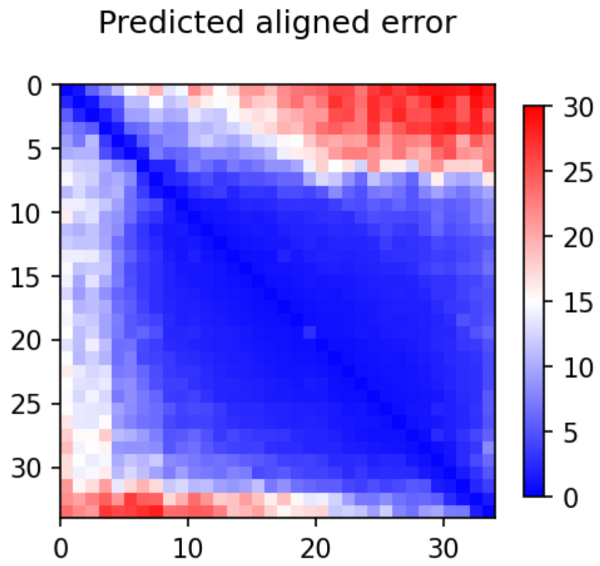

The predicted local distance difference test (pLDDT) score (0-100) is a per-residue confidence score, with values greater than 90 indicating high confidence, and values below 50 indicating low confidence.

High confidence scores Colored in Blue on structure.

Model structural quality of the AlphaFold predicted structure analyzed by PROCHECK (Laskowski et al., 1993, 1996) generated with PDBsum (https://ebi.ac.uk).

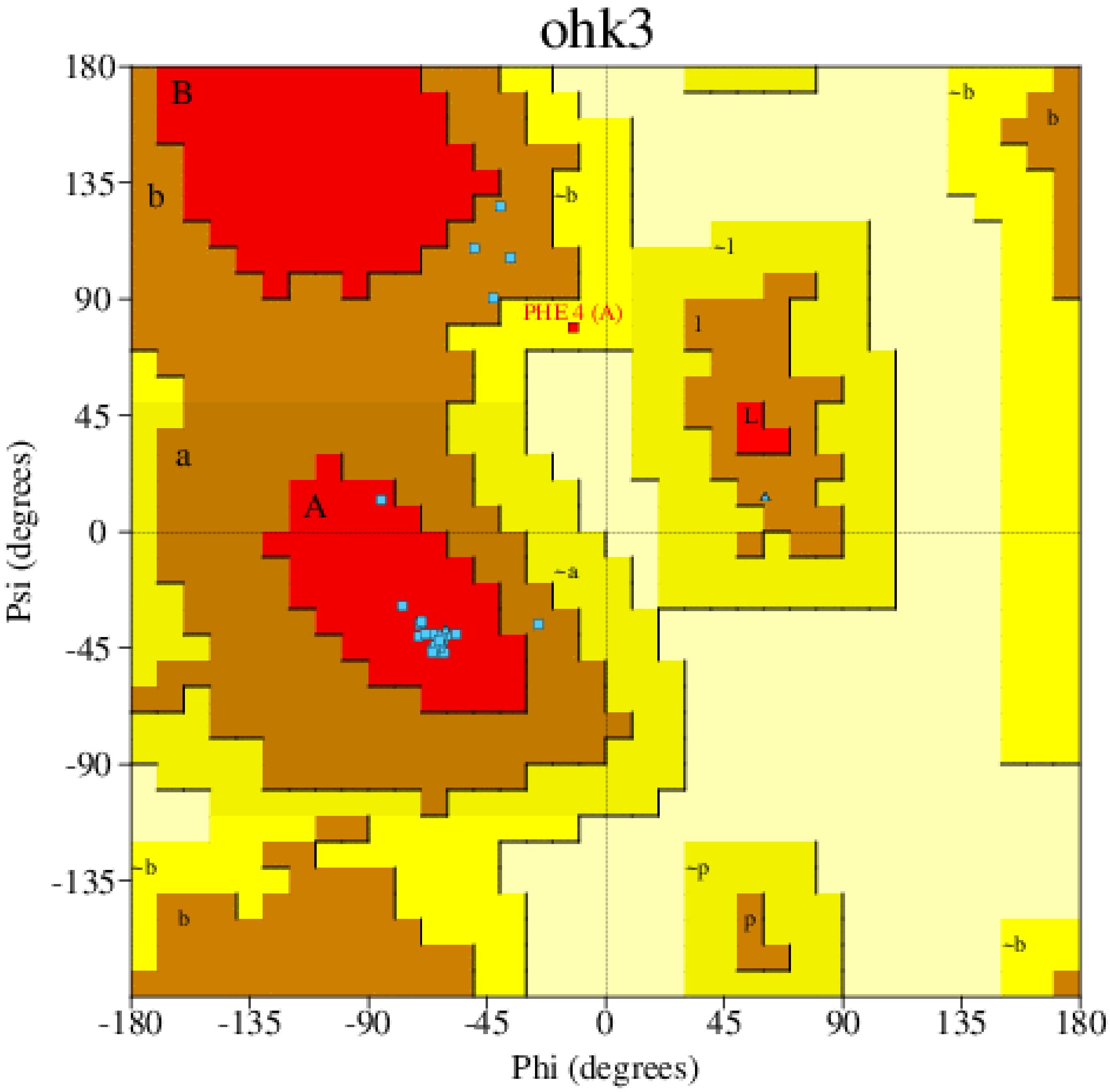

PROCHECK statistics

1. Ramachandran Plot statistics

		No. of residues	%-tage
Most favoured regions	[A,B,L]	24	85.7%*
Additional allowed regions	[a,b,l,p]	3	10.7%
Generously allowed regions	[~a,~b,~l,~p]	1	3.6%
Disallowed regions	[XX]	0	0.0%
Non-glycine and non-proline residues		28	100.0%
End-residues (excl. Gly and Pro)		1	
Glycine residues		3	
Proline residues		2	
Total number of residues		34	

Based on an analysis of **118** structures of resolution of at least **2.0** Angstroms and *R*-factor no greater than **20.0** a good quality model would be expected to have over **90%** in the most favoured regions [A,B,L].

2. G-Factors

Parameter	Score	Average Score
Dihedral angles:-		
Phi-psi distribution	0.03	
Chi1-chi2 distribution	-0.03	
Chi1 only	0.39	
Chi3 & chi4	0.21	
Omega	0.14	
		0.10
Main-chain covalent forces:-		
Main-chain bond lengths	**-2.13****	
Main-chain bond angles	**-1.95****	
		**-2.02****
OVERALL AVERAGE		-0.81*

G-factors provide a measure of how **unusual**, or out-of-the-ordinary, a property is.
Values below -0.5* - unusual
Values below **-1.0**** - highly unusual
Important note: The main-chain bond-lengths and bond angles are compared with the Engh & Huber (1991) ideal values derived from small-molecule data. Therefore, structures refined using different restraints may show apparently large deviations from normality.

Chain A (34 residues)

PROMOTIF summary

PROMOTIF documentation

Secondary structure summary

Strand	Alpha helix	3-10 helix	Other	Total residues
0 (0.0%)	26 (76.5%)	0 (0.0%)	8 (23.5%)	34

1 helix

Start	End	Type	No. resid
Ser7	Met32	H	26

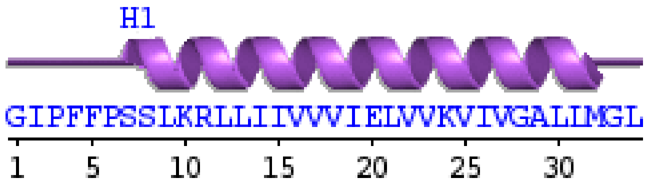
H1
GIPFFPSSLKRLLIIVVVIELVVKVIVGALIMGL
1 5 10 15 20 25 30

Molecular Topography of SP-C dog sequence in bilayer membrane. Red cylinder is the sequence that is in the hydrophobic core of the membrane.

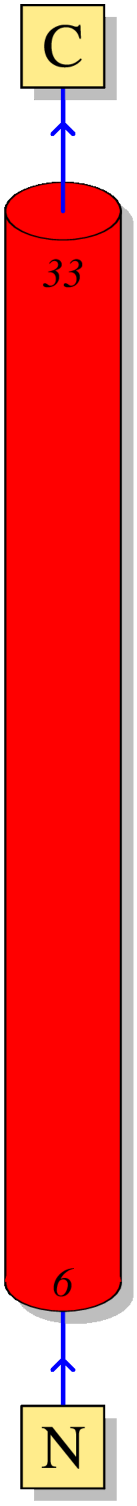

Supplement: Supplementary file 1 [file biomedicines-12-00163-s001.zip › S2.pdf]
