# Supplementary material for: Structure and Function of Canine SP-C Mimic Proteins in Synthetic Surfactant Lipid Dispersions
Source: Biomedicines. 2024 Jan 12;12(1):163. doi: 10.3390/biomedicines12010163 (PMC10813813; doi:10.3390/biomedicines12010163)
Supplement: Supplementary file 1 [file biomedicines-12-00163-s001.zip › S3.pdf]

S3 – Secondary Structure Model of Canine SP-Csf ion-lock protein using AlphaFold prediction program.

Article Title: Structure and Function of Canine SP-C Mimic Proteins in Synthetic Surfactant Lipid Dispersions

Frans J. Walther[1,2,*] & Alan J. Waring[1,3]

[1] Lundquist Institute for Biomedical Innovation at Harbor-UCLA Medical Center
1124 West Carson Street
Torrance, CA, USA

[2] Department of Pediatrics
David Geffen School of Medicine
University of California Los Angeles
405 Hilgard Avenue
Los Angeles, CA, USA

[3] Department of Medicine
David Geffen School of Medicine
University of California Los Angeles
405 Hilgard Avenue
Los Angeles, CA, USA

Modeling Protocol

Canine SP-C amino acid sequence downloaded from: https://www.uniprot.org Deposition file: P22397 · PSPC_CANLF. Secondary structure modeling SP-C canine amino acid sequence monomer (Canis lupus familiars) using primary amino acid sequence with the AI based secondary structure prediction program AlphaFold.

Canine SP-C amino acid sequence downloaded from: https://www.uniprot.org Deposition file: P22397 · PSPC_CANLF. The Cysteine residue at position 4 was mutated to serine to serve as a surrogate for Cys-palmitate in the native sequence. There was also an ion-lock amino acid pair (glutamic acid 20 – lysine 24) placed in the hydrophobic helical sequence to stabilize the helical propensity of the transmembrane domain. The secondary structure of the modified SP-C canine amino acid sequence monomer (Canis lupus familiars) was then modeled using modified amino acid sequence with the AI based secondary structure prediction program AlphaFold. The AlphaFold program was run through the Chimera X (version 1.6.1) molecular modeling environment at https://www.cgl.ucsf.edu/chimera/docs/relnotes.html.

Jumper J, Evans R, Pritzel A, et al. Highly accurate protein structure prediction with AlphaFold. *Nature*. 2021;596(7873):583-589. doi:10.1038/s41586-021-03819-2
Mirdita M, Schütze K, Moriwaki Y, Heo L, Ovchinnikov S, Steinegger M. ColabFold: making protein folding accessible to all. *Nat Methods*. 2022;19(6):679-682. doi:10.1038/s41592-022-01488-1

Input amino acid sequence for Chimera X:

>SPC_ff_ion_lock_dog
GIPSFPSSLKRLLIIVVVIELVVKVIVGALLMGL

Command Sequence for AlphaFold Prediction of SP-C dog amino acid sequence:

ChimeraX > Structure Prediction > AlphaFold > paste amino acid sequence > Predict

AlphaFold output for Canine SP-C Atomic Coordinate Data Predicted for Canine SP-Csf ion-lock Monomer Structure in PDB format:

File: SP-Csf_ion_dog.pdb

```
ATOM      1  N   GLY A   1     -26.977  -4.625  -3.419  1.00 55.34           N
ATOM      2  CA  GLY A   1     -26.637  -4.419  -2.021  1.00 55.34           C
ATOM      3  C   GLY A   1     -25.181  -4.712  -1.712  1.00 55.34           C
ATOM      4  O   GLY A   1     -24.603  -5.655  -2.255  1.00 55.34           O
ATOM      5  N   ILE A   2     -24.183  -3.844  -2.015  1.00 62.84           N
ATOM      6  CA  ILE A   2     -22.778  -4.152  -1.769  1.00 62.84           C
ATOM      7  C   ILE A   2     -22.645  -4.961  -0.480  1.00 62.84           C
ATOM      8  O   ILE A   2     -23.249  -4.620   0.539  1.00 62.84           O
ATOM      9  CB  ILE A   2     -21.924  -2.867  -1.685  1.00 62.84           C
ATOM     10  CG1 ILE A   2     -22.814  -1.625  -1.813  1.00 62.84           C
ATOM     11  CG2 ILE A   2     -20.833  -2.872  -2.759  1.00 62.84           C
ATOM     12  CD1 ILE A   2     -22.189  -0.355  -1.252  1.00 62.84           C
ATOM     13  N   PRO A   3     -22.397  -6.185  -0.533  1.00 59.02           N
ATOM     14  CA  PRO A   3     -22.258  -7.090   0.610  1.00 59.02           C
ATOM     15  C   PRO A   3     -21.448  -6.479   1.751  1.00 59.02           C
ATOM     16  O   PRO A   3     -20.522  -5.700   1.508  1.00 59.02           O
ATOM     17  CB  PRO A   3     -21.536  -8.301   0.016  1.00 59.02           C
ATOM     18  CG  PRO A   3     -21.016  -7.827  -1.303  1.00 59.02           C
ATOM     19  CD  PRO A   3     -21.678  -6.521  -1.634  1.00 59.02           C
ATOM     20  N   SER A   4     -22.088  -5.776   2.710  1.00  0.00           N
ATOM     21  CA  SER A   4     -21.608  -5.552   4.071  1.00  0.00           C
ATOM     22  C   SER A   4     -20.409  -6.421   4.370  1.00  0.00           C
ATOM     23  O   SER A   4     -20.502  -7.652   4.286  1.00  0.00           O
ATOM     24  CB  SER A   4     -22.743  -5.774   5.101  1.00  0.00           C
ATOM     25  OG  SER A   4     -23.212  -7.127   5.129  1.00  0.00           O
ATOM     26  N   PHE A   5     -19.222  -6.139   3.952  1.00 66.07           N
ATOM     27  CA  PHE A   5     -18.008  -6.855   4.325  1.00 66.07           C
ATOM     28  C   PHE A   5     -17.913  -7.007   5.838  1.00 66.07           C
ATOM     29  O   PHE A   5     -18.158  -6.054   6.580  1.00 66.07           O
ATOM     30  CB  PHE A   5     -16.769  -6.129   3.792  1.00 66.07           C
ATOM     31  CG  PHE A   5     -16.636  -6.176   2.294  1.00 66.07           C
ATOM     32  CD1 PHE A   5     -16.116  -7.300   1.663  1.00 66.07           C
ATOM     33  CD2 PHE A   5     -17.030  -5.096   1.515  1.00 66.07           C
ATOM     34  CE1 PHE A   5     -15.992  -7.347   0.276  1.00 66.07           C
ATOM     35  CE2 PHE A   5     -16.909  -5.135   0.129  1.00 66.07           C
ATOM     36  CZ  PHE A   5     -16.389  -6.261  -0.488  1.00 66.07           C
ATOM     37  N   PRO A   6     -18.069  -8.190   6.432  1.00 78.07           N
ATOM     38  CA  PRO A   6     -17.731  -8.384   7.844  1.00 78.07           C
ATOM     39  C   PRO A   6     -16.452  -7.656   8.250  1.00 78.07           C
ATOM     40  O   PRO A   6     -15.622  -7.335   7.395  1.00 78.07           O
ATOM     41  CB  PRO A   6     -17.556  -9.900   7.959  1.00 78.07           C
ATOM     42  CG  PRO A   6     -17.372 -10.372   6.553  1.00 78.07           C
ATOM     43  CD  PRO A   6     -17.900  -9.317   5.623  1.00 78.07           C
ATOM     44  N   SER A   7     -16.446  -6.839   9.236  1.00 81.92           N
ATOM     45  CA  SER A   7     -15.343  -6.102   9.844  1.00 81.92           C
ATOM     46  C   SER A   7     -14.012  -6.811   9.616  1.00 81.92           C
ATOM     47  O   SER A   7     -13.002  -6.168   9.321  1.00 81.92           O
ATOM     48  CB  SER A   7     -15.582  -5.917  11.343  1.00 81.92           C
ATOM     49  OG  SER A   7     -16.787  -5.208  11.576  1.00 81.92           O
ATOM     50  N   SER A   8     -13.903  -8.149   9.612  1.00 84.81           N
ATOM     51  CA  SER A   8     -12.683  -8.930   9.430  1.00 84.81           C
ATOM     52  C   SER A   8     -12.184  -8.848   7.992  1.00 84.81           C
ATOM     53  O   SER A   8     -10.984  -8.702   7.753  1.00 84.81           O
ATOM     54  CB  SER A   8     -12.920 -10.391   9.814  1.00 84.81           C
ATOM     55  OG  SER A   8     -13.343 -10.495  11.163  1.00 84.81           O
```

ATOM	56	N	LEU	A	9	-13.199	-8.912	7.074	1.00	87.43	N
ATOM	57	CA	LEU	A	9	-12.806	-8.849	5.671	1.00	87.43	C
ATOM	58	C	LEU	A	9	-12.312	-7.453	5.307	1.00	87.43	C
ATOM	59	O	LEU	A	9	-11.368	-7.307	4.528	1.00	87.43	O
ATOM	60	CB	LEU	A	9	-13.979	-9.240	4.767	1.00	87.43	C
ATOM	61	CG	LEU	A	9	-14.372	-10.718	4.768	1.00	87.43	C
ATOM	62	CD1	LEU	A	9	-15.637	-10.930	3.944	1.00	87.43	C
ATOM	63	CD2	LEU	A	9	-13.230	-11.576	4.235	1.00	87.43	C
ATOM	64	N	LYS	A	10	-13.041	-6.449	5.991	1.00	84.76	N
ATOM	65	CA	LYS	A	10	-12.620	-5.069	5.767	1.00	84.76	C
ATOM	66	C	LYS	A	10	-11.173	-4.858	6.202	1.00	84.76	C
ATOM	67	O	LYS	A	10	-10.393	-4.219	5.492	1.00	84.76	O
ATOM	68	CB	LYS	A	10	-13.538	-4.100	6.513	1.00	84.76	C
ATOM	69	CG	LYS	A	10	-13.214	-2.632	6.275	1.00	84.76	C
ATOM	70	CD	LYS	A	10	-14.311	-1.723	6.814	1.00	84.76	C
ATOM	71	CE	LYS	A	10	-14.396	-0.422	6.027	1.00	84.76	C
ATOM	72	NZ	LYS	A	10	-14.893	0.705	6.873	1.00	84.76	N1+
ATOM	73	N	ARG	A	11	-10.875	-5.475	7.304	1.00	91.36	N
ATOM	74	CA	ARG	A	11	-9.520	-5.347	7.830	1.00	91.36	C
ATOM	75	C	ARG	A	11	-8.514	-6.058	6.930	1.00	91.36	C
ATOM	76	O	ARG	A	11	-7.435	-5.529	6.658	1.00	91.36	O
ATOM	77	CB	ARG	A	11	-9.440	-5.907	9.251	1.00	91.36	C
ATOM	78	CG	ARG	A	11	-8.085	-5.714	9.914	1.00	91.36	C
ATOM	79	CD	ARG	A	11	-8.096	-6.175	11.365	1.00	91.36	C
ATOM	80	NE	ARG	A	11	-8.708	-5.182	12.242	1.00	91.36	N
ATOM	81	CZ	ARG	A	11	-8.805	-5.294	13.564	1.00	91.36	C
ATOM	82	NH1	ARG	A	11	-8.328	-6.364	14.190	1.00	91.36	N1+
ATOM	83	NH2	ARG	A	11	-9.382	-4.330	14.266	1.00	91.36	N
ATOM	84	N	LEU	A	12	-8.813	-7.099	6.458	1.00	94.39	N
ATOM	85	CA	LEU	A	12	-7.951	-7.866	5.564	1.00	94.39	C
ATOM	86	C	LEU	A	12	-7.693	-7.102	4.270	1.00	94.39	C
ATOM	87	O	LEU	A	12	-6.554	-7.027	3.805	1.00	94.39	O
ATOM	88	CB	LEU	A	12	-8.579	-9.226	5.251	1.00	94.39	C
ATOM	89	CG	LEU	A	12	-7.749	-10.172	4.382	1.00	94.39	C
ATOM	90	CD1	LEU	A	12	-6.467	-10.569	5.106	1.00	94.39	C
ATOM	91	CD2	LEU	A	12	-8.562	-11.407	4.007	1.00	94.39	C
ATOM	92	N	LEU	A	13	-8.638	-6.522	3.720	1.00	94.70	N
ATOM	93	CA	LEU	A	13	-8.537	-5.794	2.459	1.00	94.70	C
ATOM	94	C	LEU	A	13	-7.564	-4.626	2.583	1.00	94.70	C
ATOM	95	O	LEU	A	13	-6.732	-4.410	1.699	1.00	94.70	O
ATOM	96	CB	LEU	A	13	-9.912	-5.285	2.022	1.00	94.70	C
ATOM	97	CG	LEU	A	13	-10.660	-6.143	1.000	1.00	94.70	C
ATOM	98	CD1	LEU	A	13	-12.166	-5.964	1.158	1.00	94.70	C
ATOM	99	CD2	LEU	A	13	-10.220	-5.791	-0.417	1.00	94.70	C
ATOM	100	N	ILE	A	14	-7.678	-3.965	3.673	1.00	96.03	N
ATOM	101	CA	ILE	A	14	-6.836	-2.798	3.910	1.00	96.03	C
ATOM	102	C	ILE	A	14	-5.370	-3.222	3.968	1.00	96.03	C
ATOM	103	O	ILE	A	14	-4.508	-2.582	3.362	1.00	96.03	O
ATOM	104	CB	ILE	A	14	-7.235	-2.069	5.213	1.00	96.03	C
ATOM	105	CG1	ILE	A	14	-8.646	-1.484	5.088	1.00	96.03	C
ATOM	106	CG2	ILE	A	14	-6.217	-0.976	5.554	1.00	96.03	C
ATOM	107	CD1	ILE	A	14	-9.238	-1.006	6.406	1.00	96.03	C
ATOM	108	N	ILE	A	15	-5.163	-4.326	4.639	1.00	97.18	N
ATOM	109	CA	ILE	A	15	-3.804	-4.831	4.801	1.00	97.18	C
ATOM	110	C	ILE	A	15	-3.220	-5.189	3.437	1.00	97.18	C
ATOM	111	O	ILE	A	15	-2.084	-4.823	3.125	1.00	97.18	O
ATOM	112	CB	ILE	A	15	-3.766	-6.058	5.740	1.00	97.18	C
ATOM	113	CG1	ILE	A	15	-4.158	-5.652	7.165	1.00	97.18	C
ATOM	114	CG2	ILE	A	15	-2.381	-6.712	5.718	1.00	97.18	C
ATOM	115	CD1	ILE	A	15	-4.390	-6.830	8.102	1.00	97.18	C
ATOM	116	N	VAL	A	16	-4.015	-5.818	2.688	1.00	96.91	N
ATOM	117	CA	VAL	A	16	-3.575	-6.261	1.369	1.00	96.91	C
ATOM	118	C	VAL	A	16	-3.279	-5.050	0.488	1.00	96.91	C

ATOM	119	O	VAL	A	16	-2.272	-5.022	-0.222	1.00	96.91	O
ATOM	120	CB	VAL	A	16	-4.630	-7.166	0.694	1.00	96.91	C
ATOM	121	CG1	VAL	A	16	-4.315	-7.349	-0.789	1.00	96.91	C
ATOM	122	CG2	VAL	A	16	-4.702	-8.519	1.399	1.00	96.91	C
ATOM	123	N	VAL	A	17	-4.088	-4.097	0.610	1.00	97.15	N
ATOM	124	CA	VAL	A	17	-3.941	-2.885	-0.189	1.00	97.15	C
ATOM	125	C	VAL	A	17	-2.649	-2.167	0.194	1.00	97.15	C
ATOM	126	O	VAL	A	17	-1.888	-1.738	-0.676	1.00	97.15	O
ATOM	127	CB	VAL	A	17	-5.149	-1.938	-0.012	1.00	97.15	C
ATOM	128	CG1	VAL	A	17	-4.834	-0.551	-0.569	1.00	97.15	C
ATOM	129	CG2	VAL	A	17	-6.387	-2.521	-0.691	1.00	97.15	C
ATOM	130	N	VAL	A	18	-2.518	-2.099	1.435	1.00	97.55	N
ATOM	131	CA	VAL	A	18	-1.328	-1.418	1.935	1.00	97.55	C
ATOM	132	C	VAL	A	18	-0.076	-2.165	1.481	1.00	97.55	C
ATOM	133	O	VAL	A	18	0.878	-1.553	0.996	1.00	97.55	O
ATOM	134	CB	VAL	A	18	-1.348	-1.297	3.475	1.00	97.55	C
ATOM	135	CG1	VAL	A	18	-0.020	-0.746	3.991	1.00	97.55	C
ATOM	136	CG2	VAL	A	18	-2.508	-0.411	3.925	1.00	97.55	C
ATOM	137	N	ILE	A	19	-0.093	-3.425	1.649	1.00	98.28	N
ATOM	138	CA	ILE	A	19	1.047	-4.250	1.265	1.00	98.28	C
ATOM	139	C	ILE	A	19	1.294	-4.123	-0.236	1.00	98.28	C
ATOM	140	O	ILE	A	19	2.438	-3.980	-0.673	1.00	98.28	O
ATOM	141	CB	ILE	A	19	0.827	-5.731	1.649	1.00	98.28	C
ATOM	142	CG1	ILE	A	19	0.826	-5.893	3.173	1.00	98.28	C
ATOM	143	CG2	ILE	A	19	1.895	-6.620	1.004	1.00	98.28	C
ATOM	144	CD1	ILE	A	19	0.434	-7.286	3.647	1.00	98.28	C
ATOM	145	N	GLU	A	20	0.220	-4.236	-1.008	1.00	97.65	N
ATOM	146	CA	GLU	A	20	0.358	-4.114	-2.456	1.00	97.65	C
ATOM	147	C	GLU	A	20	0.995	-2.781	-2.840	1.00	97.65	C
ATOM	148	O	GLU	A	20	1.845	-2.727	-3.731	1.00	97.65	O
ATOM	149	CB	GLU	A	20	-1.002	-4.263	-3.141	1.00	97.65	C
ATOM	150	CG	GLU	A	20	-1.378	-5.704	-3.457	1.00	97.65	C
ATOM	151	CD	GLU	A	20	-0.885	-6.168	-4.818	1.00	97.65	C
ATOM	152	OE1	GLU	A	20	0.102	-6.937	-4.876	1.00	97.65	O
ATOM	153	OE2	GLU	A	20	-1.489	-5.759	-5.834	1.00	97.65	O1-
ATOM	154	N	LEU	A	21	0.623	-1.820	-2.188	1.00	98.27	N
ATOM	155	CA	LEU	A	21	1.142	-0.480	-2.441	1.00	98.27	C
ATOM	156	C	LEU	A	21	2.629	-0.403	-2.111	1.00	98.27	C
ATOM	157	O	LEU	A	21	3.414	0.144	-2.889	1.00	98.27	O
ATOM	158	CB	LEU	A	21	0.371	0.557	-1.621	1.00	98.27	C
ATOM	159	CG	LEU	A	21	0.664	2.025	-1.936	1.00	98.27	C
ATOM	160	CD1	LEU	A	21	0.030	2.418	-3.266	1.00	98.27	C
ATOM	161	CD2	LEU	A	21	0.163	2.924	-0.812	1.00	98.27	C
ATOM	162	N	VAL	A	22	2.983	-0.937	-0.969	1.00	97.75	N
ATOM	163	CA	VAL	A	22	4.372	-0.923	-0.523	1.00	97.75	C
ATOM	164	C	VAL	A	22	5.238	-1.713	-1.501	1.00	97.75	C
ATOM	165	O	VAL	A	22	6.312	-1.256	-1.899	1.00	97.75	O
ATOM	166	CB	VAL	A	22	4.516	-1.500	0.903	1.00	97.75	C
ATOM	167	CG1	VAL	A	22	5.989	-1.639	1.283	1.00	97.75	C
ATOM	168	CG2	VAL	A	22	3.782	-0.619	1.912	1.00	97.75	C
ATOM	169	N	VAL	A	23	4.825	-2.784	-1.892	1.00	97.68	N
ATOM	170	CA	VAL	A	23	5.569	-3.653	-2.798	1.00	97.68	C
ATOM	171	C	VAL	A	23	5.770	-2.950	-4.139	1.00	97.68	C
ATOM	172	O	VAL	A	23	6.860	-2.996	-4.714	1.00	97.68	O
ATOM	173	CB	VAL	A	23	4.850	-5.005	-3.007	1.00	97.68	C
ATOM	174	CG1	VAL	A	23	5.531	-5.814	-4.110	1.00	97.68	C
ATOM	175	CG2	VAL	A	23	4.817	-5.797	-1.702	1.00	97.68	C
ATOM	176	N	LYS	A	24	4.710	-2.315	-4.584	1.00	96.92	N
ATOM	177	CA	LYS	A	24	4.799	-1.603	-5.856	1.00	96.92	C
ATOM	178	C	LYS	A	24	5.839	-0.488	-5.790	1.00	96.92	C
ATOM	179	O	LYS	A	24	6.567	-0.254	-6.757	1.00	96.92	O
ATOM	180	CB	LYS	A	24	3.437	-1.027	-6.245	1.00	96.92	C
ATOM	181	CG	LYS	A	24	2.536	-2.010	-6.980	1.00	96.92	C

ATOM	182	CD	LYS	A	24	1.153	-1.422	-7.228	1.00	96.92	C
ATOM	183	CE	LYS	A	24	0.331	-2.300	-8.162	1.00	96.92	C
ATOM	184	NZ	LYS	A	24	-1.109	-1.904	-8.169	1.00	96.92	N1+
ATOM	185	N	VAL	A	25	5.869	0.166	-4.721	1.00	96.75	N
ATOM	186	CA	VAL	A	25	6.843	1.236	-4.529	1.00	96.75	C
ATOM	187	C	VAL	A	25	8.255	0.656	-4.528	1.00	96.75	C
ATOM	188	O	VAL	A	25	9.151	1.185	-5.191	1.00	96.75	O
ATOM	189	CB	VAL	A	25	6.583	2.011	-3.218	1.00	96.75	C
ATOM	190	CG1	VAL	A	25	7.725	2.985	-2.931	1.00	96.75	C
ATOM	191	CG2	VAL	A	25	5.250	2.753	-3.292	1.00	96.75	C
ATOM	192	N	ILE	A	26	8.394	-0.427	-3.829	1.00	97.05	N
ATOM	193	CA	ILE	A	26	9.712	-1.043	-3.714	1.00	97.05	C
ATOM	194	C	ILE	A	26	10.164	-1.553	-5.080	1.00	97.05	C
ATOM	195	O	ILE	A	26	11.306	-1.328	-5.488	1.00	97.05	O
ATOM	196	CB	ILE	A	26	9.709	-2.195	-2.685	1.00	97.05	C
ATOM	197	CG1	ILE	A	26	9.401	-1.658	-1.282	1.00	97.05	C
ATOM	198	CG2	ILE	A	26	11.048	-2.939	-2.704	1.00	97.05	C
ATOM	199	CD1	ILE	A	26	9.268	-2.741	-0.220	1.00	97.05	C
ATOM	200	N	VAL	A	27	9.282	-2.207	-5.695	1.00	96.52	N
ATOM	201	CA	VAL	A	27	9.597	-2.753	-7.011	1.00	96.52	C
ATOM	202	C	VAL	A	27	9.889	-1.615	-7.986	1.00	96.52	C
ATOM	203	O	VAL	A	27	10.829	-1.697	-8.781	1.00	96.52	O
ATOM	204	CB	VAL	A	27	8.447	-3.634	-7.549	1.00	96.52	C
ATOM	205	CG1	VAL	A	27	8.727	-4.066	-8.988	1.00	96.52	C
ATOM	206	CG2	VAL	A	27	8.244	-4.853	-6.651	1.00	96.52	C
ATOM	207	N	GLY	A	28	9.082	-0.614	-7.880	1.00	96.83	N
ATOM	208	CA	GLY	A	28	9.296	0.556	-8.716	1.00	96.83	C
ATOM	209	C	GLY	A	28	10.641	1.217	-8.485	1.00	96.83	C
ATOM	210	O	GLY	A	28	11.312	1.622	-9.437	1.00	96.83	O
ATOM	211	N	ALA	A	29	11.039	1.248	-7.281	1.00	96.13	N
ATOM	212	CA	ALA	A	29	12.322	1.844	-6.917	1.00	96.13	C
ATOM	213	C	ALA	A	29	13.485	0.996	-7.425	1.00	96.13	C
ATOM	214	O	ALA	A	29	14.502	1.531	-7.872	1.00	96.13	O
ATOM	215	CB	ALA	A	29	12.417	2.019	-5.403	1.00	96.13	C
ATOM	216	N	LEU	A	30	13.298	-0.293	-7.412	1.00	94.96	N
ATOM	217	CA	LEU	A	30	14.331	-1.228	-7.845	1.00	94.96	C
ATOM	218	C	LEU	A	30	14.546	-1.141	-9.352	1.00	94.96	C
ATOM	219	O	LEU	A	30	15.682	-1.211	-9.826	1.00	94.96	O
ATOM	220	CB	LEU	A	30	13.956	-2.660	-7.453	1.00	94.96	C
ATOM	221	CG	LEU	A	30	14.104	-3.019	-5.974	1.00	94.96	C
ATOM	222	CD1	LEU	A	30	13.431	-4.356	-5.683	1.00	94.96	C
ATOM	223	CD2	LEU	A	30	15.575	-3.058	-5.577	1.00	94.96	C
ATOM	224	N	LEU	A	31	13.386	-0.878	-10.070	1.00	94.76	N
ATOM	225	CA	LEU	A	31	13.458	-0.825	-11.526	1.00	94.76	C
ATOM	226	C	LEU	A	31	14.066	0.494	-11.992	1.00	94.76	C
ATOM	227	O	LEU	A	31	14.713	0.548	-13.040	1.00	94.76	O
ATOM	228	CB	LEU	A	31	12.066	-1.003	-12.139	1.00	94.76	C
ATOM	229	CG	LEU	A	31	11.430	-2.386	-11.987	1.00	94.76	C
ATOM	230	CD1	LEU	A	31	10.011	-2.381	-12.545	1.00	94.76	C
ATOM	231	CD2	LEU	A	31	12.281	-3.445	-12.681	1.00	94.76	C
ATOM	232	N	MET	A	32	13.833	1.494	-11.140	1.00	93.08	N
ATOM	233	CA	MET	A	32	14.332	2.811	-11.523	1.00	93.08	C
ATOM	234	C	MET	A	32	15.789	2.983	-11.107	1.00	93.08	C
ATOM	235	O	MET	A	32	16.483	3.868	-11.611	1.00	93.08	O
ATOM	236	CB	MET	A	32	13.476	3.914	-10.899	1.00	93.08	C
ATOM	237	CG	MET	A	32	12.920	4.905	-11.909	1.00	93.08	C
ATOM	238	SD	MET	A	32	11.131	4.654	-12.230	1.00	93.08	S
ATOM	239	CE	MET	A	32	10.794	6.080	-13.299	1.00	93.08	C
ATOM	240	N	GLY	A	33	16.538	1.864	-10.684	1.00	85.12	N
ATOM	241	CA	GLY	A	33	17.967	1.922	-10.419	1.00	85.12	C
ATOM	242	C	GLY	A	33	18.331	2.895	-9.314	1.00	85.12	C
ATOM	243	O	GLY	A	33	19.480	3.330	-9.216	1.00	85.12	O
ATOM	244	N	LEU	A	34	17.286	3.355	-8.550	1.00	68.36	N

```
ATOM    245  CA  LEU A  34      17.706   4.114  -7.376  1.00 68.36           C
ATOM    246  C   LEU A  34      18.452   3.221  -6.390  1.00 68.36           C
ATOM    247  O   LEU A  34      19.460   3.633  -5.813  1.00 68.36           O
ATOM    248  CB  LEU A  34      16.495   4.752  -6.690  1.00 68.36           C
ATOM    249  CG  LEU A  34      15.912   5.995  -7.363  1.00 68.36           C
ATOM    250  CD1 LEU A  34      14.503   6.267  -6.845  1.00 68.36           C
ATOM    251  CD2 LEU A  34      16.814   7.203  -7.130  1.00 68.36           C
TER
END
```


Quality of AlphaFold Best Predicted Model

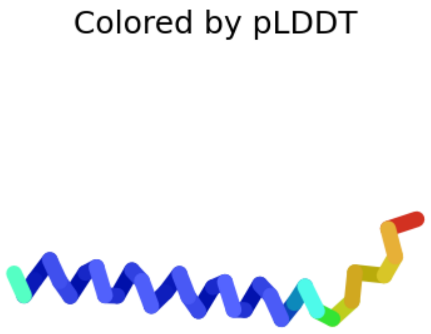

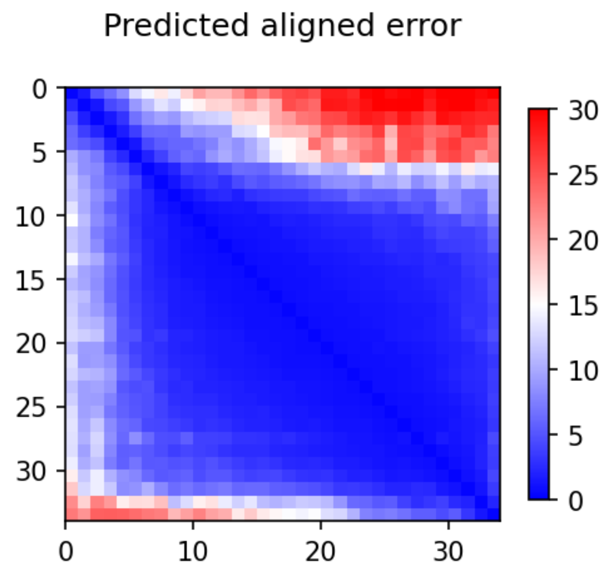

The predicted local distance difference test (pLDDT) score (0-100) is a per-residue confidence score, with values greater than 90 indicating high confidence, and values below 50 indicating low confidence.

High confidence scores Colored in Blue on structure.

Model structural quality of the AlphaFold predicted structure analyzed by PROCHECK (Laskowski et al., 1993, 1996) generated with PDBsum (https://ebi.ac.uk).

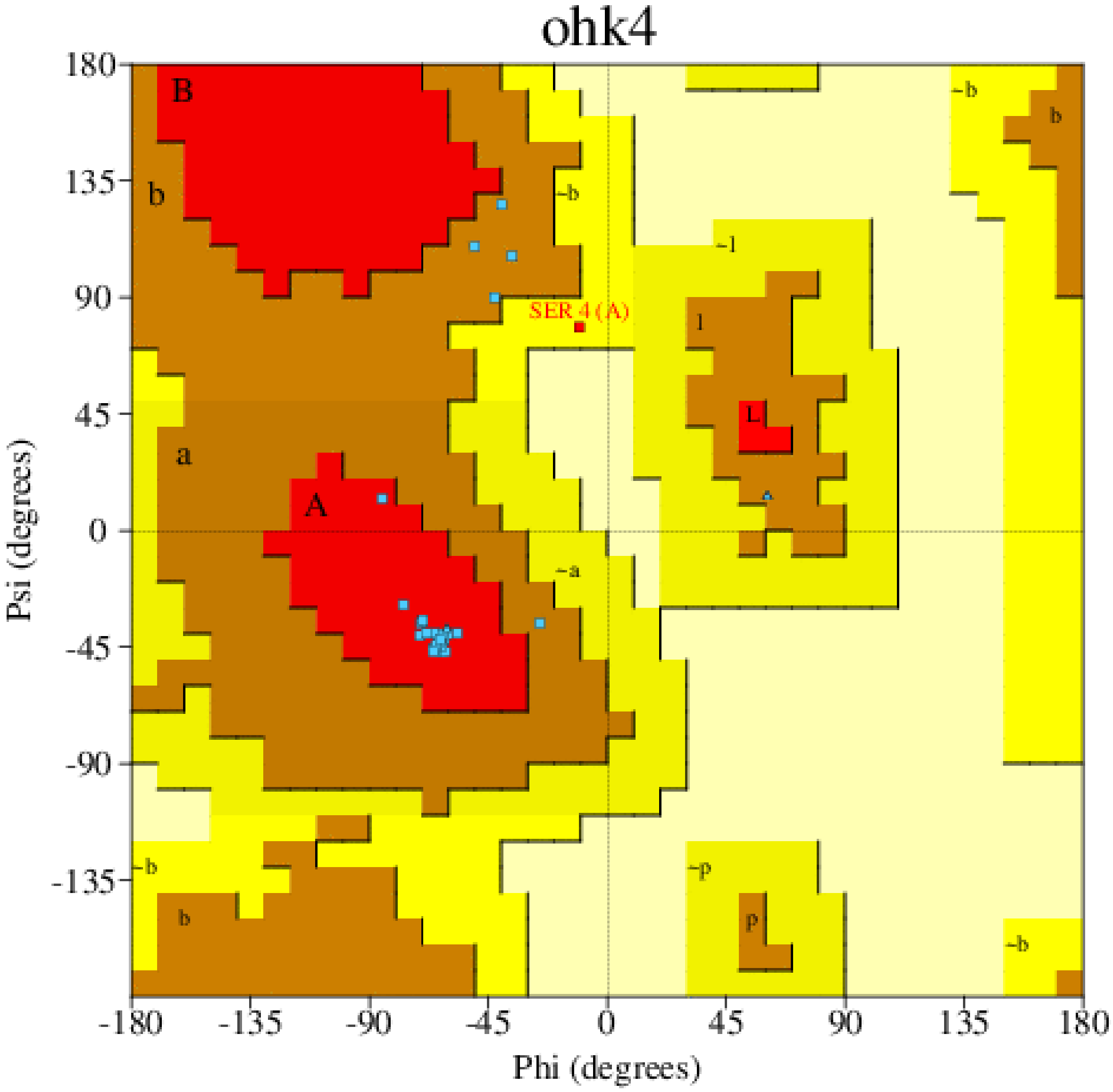

PROCHECK statistics

1. Ramachandran Plot statistics

		No. of residues	%-tage
Most favoured regions	[A,B,L]	24	85.7%*
Additional allowed regions	[a,b,l,p]	3	10.7%
Generously allowed regions	[~a,~b,~l,~p]	1	3.6%
Disallowed regions	[XX]	0	0.0%
Non-glycine and non-proline residues		28	100.0%
End-residues (excl. Gly and Pro)		1	
Glycine residues		3	
Proline residues		2	
Total number of residues		34	

Based on an analysis of **118** structures of resolution of at least **2.0** Angstroms and *R*-factor no greater than **20.0** a good quality model would be expected to have over **90%** in the most favoured regions [A,B,L].

2. G-Factors

Parameter	Score	Average Score
Dihedral angles:-		
Phi-psi distribution	0.03	
Chi1-chi2 distribution	0.03	
Chi1 only	0.45	
Chi3 & chi4	0.21	
Omega	0.14	
		0.12
Main-chain covalent forces:-		
Main-chain bond lengths	**-2.14****	
Main-chain bond angles	**-1.96****	
		**-2.03****
OVERALL AVERAGE		-0.80*

G-factors provide a measure of how **unusual**, or out-of-the-ordinary, a property is.
Values below -0.5* - unusual
Values below **-1.0**** - highly unusual
Important note: The main-chain bond-lengths and bond angles are compared with the Engh & Huber (1991) ideal values derived from small-molecule data. Therefore, structures refined using different restraints may show apparently large deviations from normality.

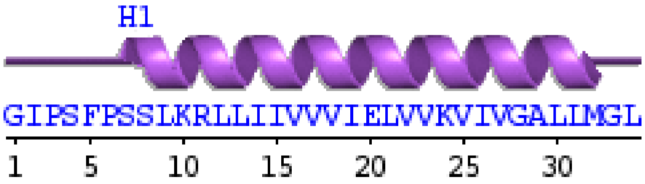

PROMOTIF summary

PROMOTIF documentation

Secondary structure summary

Strand	Alpha helix	3-10 helix	Other	Total residues
0 (0.0%)	26 (76.5%)	0 (0.0%)	8 (23.5%)	34

1 helix

Start	End	Type	No. resid
Ser7	Met32	H	26

Molecular Topography of SP-Csf ion-lock dog sequence in bilayer membrane. Red cylinder is the sequence that is in the hydrophobic core of the membrane.

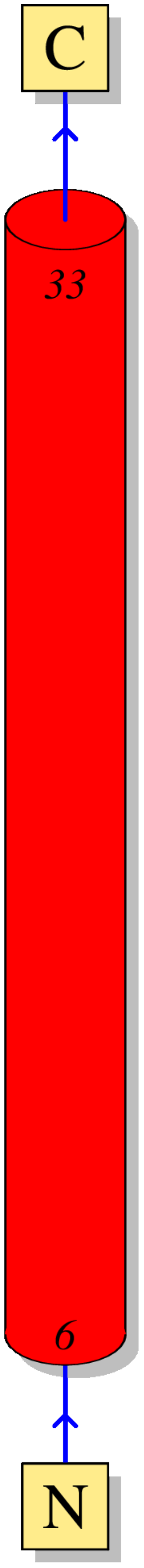
C
33
6
N
