# Supplementary material for: Structure and Function of Canine SP-C Mimic Proteins in Synthetic Surfactant Lipid Dispersions
Source: Biomedicines. 2024 Jan 12;12(1):163. doi: 10.3390/biomedicines12010163 (PMC10813813; doi:10.3390/biomedicines12010163)
Supplement: Supplementary file 1 [file biomedicines-12-00163-s001.zip › S4.pdf]

S4 – FTIR Determination of peptide conformation and phospholipid acyl chain orientation in surfactant lipid multilayers.

Article Title: Structure and Function of Canine SP-C Mimic Proteins in Synthetic Surfactant Lipid Dispersions

Frans J. Walther[1,2,*] & Alan J. Waring[1,3]

[1] Lundquist Institute for Biomedical Innovation at Harbor-UCLA Medical Center
1124 West Carson Street
Torrance, CA, USA

[2] Department of Pediatrics
David Geffen School of Medicine
University of California Los Angeles
405 Hilgard Avenue
Los Angeles, CA, USA

[3] Department of Medicine
David Geffen School of Medicine
University of California Los Angeles
405 Hilgard Avenue
Los Angeles, CA, USA

Determination of Phospholipid Acyl Chain Orientation in Surfactant Lipid Multilayers

The average tilt angle of the acyl chains was estimated by polarized FTIR measurements of surfactant lipid films. These measurements were made by determination of the acyl chain absorption of the symmetric stretching of the antisymmetric CH_2 band at 2918 cm^{-1} and the CH_2 symmetric band at 2850 cm^{-1} with polarized light parallel (*All*) to and perpendicular (*AI*) to the normal of the ATR surface. The dichroic ratio R was then calculated from the absorption in the parallel mode divided by that of the parallel mode (*R=AII/AI*). Using this metric an order parameter (*S*, equation 1) was estimated (1) where *Exyz* are components of the electric field vector of the evanescent wave and a is the angle between the transition dipole moment and the long axis of the acyl chain.

$$(1)\ S = 2(E^2_x - R_{exp}E^2_y + E^2_z) / (3(\cos\alpha)^2 - 1)\ (E^2_x - R_{exp}E^2_y - E^2_z)$$

The order parameter is then used to calculate the average angle β of the long axis of the acyl chain with respect to the surface normal of the ATR sample crystal using equation 2.

$$(2)\ S = 3\ (\cos\beta)^2 - 1 / 2$$

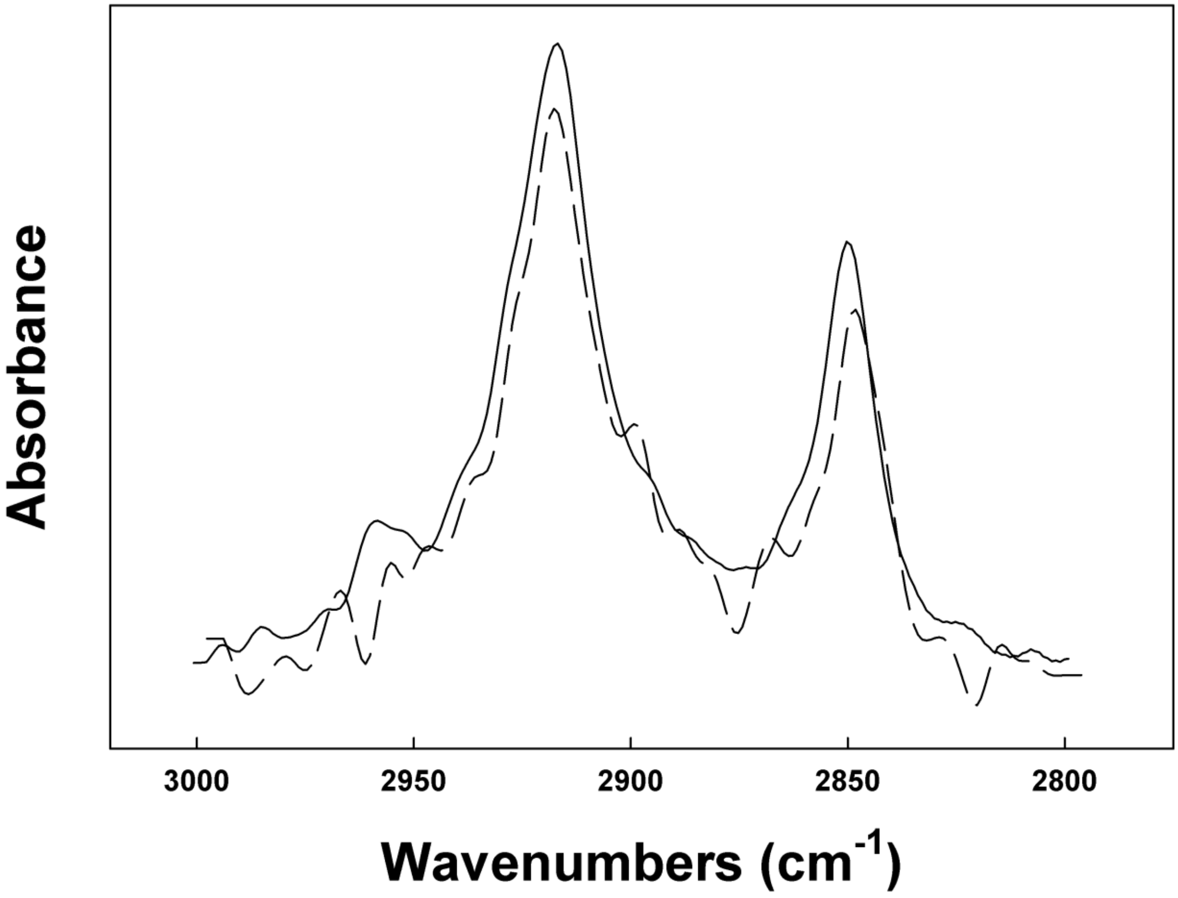

Figure S1. Example of polarized FTIR spectrum of acyl chain stretching modes of surfactant lipid – SP-Cff ion-lock peptide sample.

The absorption peek around 2918 cm^{-1} is the antisymmetric CH_2 band and the peek at 2850 cm^{-1} represents the symmetric CH_2 stretching band. The solid line is the spectrum of the parallel absorption to the ATR surface for the sample while the dashed line represents perpendicular acyl chain absorption spectrum to the ATR surface of the same sample.

Surfactant Lipid Acyl chain mean angle of insertion: 33.1^{o} $\pm$ 2.01^{o}

Reference

Goormaghtigh E, Raussens V, Ruysschaert JM. Attenuated total reflection infrared spectroscopy of proteins and lipids in biological membranes. *Biochim Biophys Acta*. 1999;1422(2):105-185. doi:10.1016/s0304-4157(99)00004-0.

Estimation of the contributions of the amide I peptide bands from the surfactant lipid multilayers with BYL plus SP-Cff ion-lock peptide. Spectral subtraction of each of the peptides alone in surfactant multilayers from the combination of the two peptides in lipid indicates that the overall conformation of each peptide is not altered when the two peptides are included in the same peptide-lipid ensemble (Figure 2).

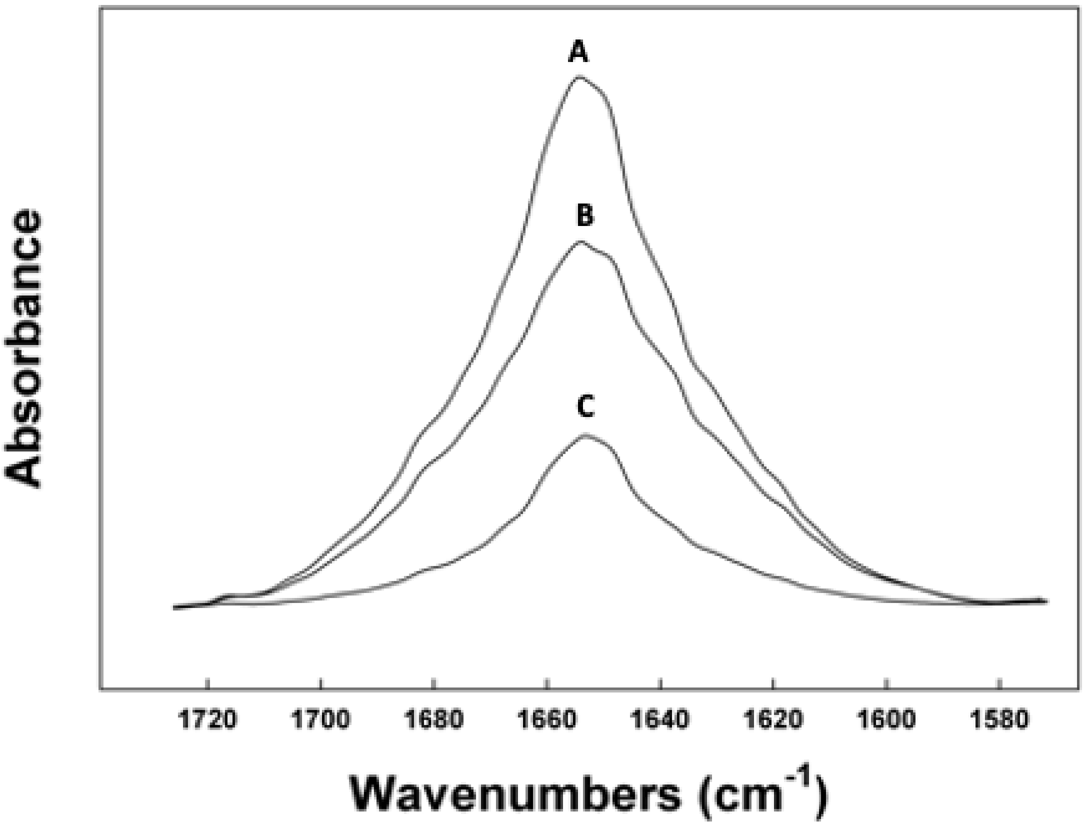

Figure S2. FTIR spectra of amide I conformational bands of BYL+SP-Cff, B-YL and SP-Cff ion-lock peptides in surfactant lipids.

(A) BYL+SP-Cff ion-lock 2:1 (wt:wt) peptides in synthetic surfactant lipids (1:10, mole:mole).
(B) BYL FTIR spectrum derived from spectral subtraction of SP-Cff ion-lock spectrum from the combination ensemble in spectrum A.
(C) SP-Cff ion-lock FTIR spectrum derived from spectral subtraction of BYL spectrum from the combination ensemble in spectrum A.
